# Near‐Theoretical Redox‐Site Utilization and Fast Zn^2+^ Storage in a Polyimide‐CNT Cathode

**DOI:** 10.1002/smll.75112

**Published:** 2026-08-08

**Authors:** Heba H. Farrag, Jônatas Faleiro Berbigier, Ailsa K. Edward, Dwight S. Seferos

**Affiliations:** ^1^ Department of Chemistry Lash Miller Chemical Laboratories University of Toronto Toronto Ontario Canada; ^2^ Department of Chemistry Faculty of Science Cairo University Cairo Egypt; ^3^ Department of Chemical Engineering and Applied Chemistry University of Toronto Toronto Ontario Canada

**Keywords:** aqueous zinc‐ion batteries, carbon‐nanotube hybrids, high‐rate energy storage, organic electrode materials, polyimide cathodes, pyrene‐tetrone

## Abstract

Achieving high utilization efficiency together with fast electrochemical transport remains a central challenge for organic cathodes in aqueous zinc‐ion batteries. Herein, a π‐conjugated imide‐linked polyimide based on pyrene‐4,5,9,10‐tetraone and pyromellitic dianhydride (PMDA–PTO) is reported, where a carbonyl‐rich conjugated polyimide framework is integrated with a conductive carbon nanotube (CNT) network to promote electronic percolation and facilitate efficient utilization of redox‐active sites together with fast and durable Zn‐ion storage. The PMDA–PTO–CNT composite delivers a discharge capacity of 328 mAh g^−1^ at 0.05 A g^−1^, corresponding to ∼98% utilization of its theoretical capacity, and maintains 316 mAh g^−1^ at 20 A g^−1^, while sustaining stable electrochemical operation over 10 000 cycles at 5 A g^−1^. Electrochemical analyses reveal reduced polarization, enhanced pseudocapacitive behavior, and improved electrochemical transport within the CNT‐containing electrode architecture. Ex situ Fourier transform infrared (FTIR), X‐ray photoelectron spectroscopy (XPS), and Grazing‐incidence wide‐angle X‐ray scattering (GIWAXS) measurements support a reversible carbonyl‐centered cation‐storage mechanism within a structurally disordered polymer framework that retains only short‐range organization during cycling, while density functional theory (DFT) calculations corroborate the multistep delocalized redox behavior of the conjugated backbone. Collectively, these findings provide insight into structure–transport relationships in quinone‐based polymer cathodes and highlight how electronic percolation and redox‐site accessibility enable high utilization, fast kinetics, and long‐term stability in aqueous Zn‐ion batteries.

## Graphical abstract

A CNT‐integrated PTO‐based π‐conjugated polyimide cathode achieves near‐theoretical redox‐site utilization, ultrafast Zn^2+^ storage up to 20 A g^−1^, and exceptional cycling stability over 10 000 cycles in aqueous zinc‐ion batteries.

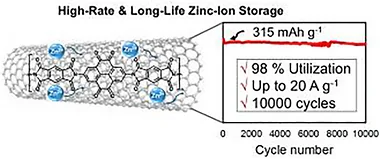

## 1 Introduction

The accelerating depletion of fossil fuel resources, together with their escalating environmental consequences, has intensified global efforts to develop sustainable energy technologies and efficient energy storage systems [1, 2, 3]. While lithium‐ion batteries (LIBs) currently dominate the rechargeable battery market, their large‐scale deployment faces growing challenges, including safety concerns associated with flammable organic electrolytes, limited lithium resources, and environmental impacts of extraction [4, 5, 6]. These limitations have motivated the search for alternative battery technologies.

Rechargeable aqueous zinc‐ion batteries (ZIBs) have emerged as promising candidates due to the intrinsic advantages of metallic zinc anodes, including high theoretical capacity, low redox potential, natural abundance, and compatibility with safe aqueous electrolytes [7, 8, 9, 10]. Despite these advantages, the development of high‐performance cathode materials remains a key bottleneck limiting the overall performance of ZIB systems [6, 11].

Organic electrode materials composed of earth‐abundant elements have attracted increasing attention as sustainable alternatives to inorganic cathodes [12, 13, 14]. Quinone‐based compounds, in particular, enable reversible Zn^2+^ storage through carbonyl redox chemistry and offer structural tunability [15, 16, 17, 18, 19, 20, 21, 22]. However, small molecule quinones typically suffer from dissolution in aqueous electrolytes, leading to rapid capacity fading and poor cycling stability [23, 24, 25]. Polymerization strategies have been widely explored to suppress dissolution and improve structural stability [26, 27], yet polymer‐based quinone cathodes often exhibit sluggish charge transport and incomplete utilization of redox‐active sites due to disordered molecular packing and limited electronic conductivity.

Recent advances have demonstrated that high‐capacity and high‐voltage cathode materials for aqueous zinc‐ion batteries can be achieved through both inorganic frameworks and advanced organic systems, enabling improved energy density and electrochemical performance [28, 29, 30, 31, 32, 33, 34]. However, these systems often rely on well‐defined crystalline structures or complex material architectures, where high performance is achieved at the expense of synthetic complexity or limited structural flexibility. In contrast, organic polymer cathodes, while offering tunability and sustainability, still suffer from incomplete utilization of redox‐active sites and limited rate capability, particularly in structurally disordered systems [35, 36]. Although recent advances demonstrate the potential of high‐energy aqueous Zn‐ion batteries, the factors governing high utilization and fast electrochemical transport in structurally disordered organic polymer cathodes remain poorly understood. The present work provides insight into how enhanced electronic percolation can contribute to achieving high utilization and fast Zn^2+^ storage without long‐range structural order.

While conductive carbon frameworks are commonly introduced to enhance charge transport in organic electrodes [37, 38, 39], structurally disordered polymer cathodes often continue to suffer from incomplete utilization of redox‐active sites and limited rate capability due to poor electronic connectivity, highlighting the challenge of achieving near‐complete utilization and fast Zn^2^^+^ storage without relying on long‐range structural order.

Herein, we report a π‐conjugated polyimide cathode based on pyrene‐4,5,9,10‐tetraone (PTO) and pyromellitic dianhydride (PMDA). PTO was selected as a redox‐active building block because its four carbonyl groups provide a high theoretical capacity, while incorporation into a polyimide framework is expected to suppress dissolution and improve structural stability during cycling. In addition to serving as a linker for polymer formation, PMDA contributes additional redox‐active carbonyl groups, increasing the density of electrochemically active sites within the polymer backbone and enhancing the overall theoretical capacity of the material. By incorporating a conductive carbon nanotube (CNT) framework, electronic percolation within the electrode is enhanced, improving access to redox‐active sites that may otherwise be transport‐limited within the polymer matrix. The resulting PMDA–PTO–CNT electrode exhibits high reversible capacity, excellent rate capability, and long‐term cycling stability in aqueous zinc‐ion batteries. These characteristics are maintained across a range of electrode compositions, including higher active‐material fractions and increased mass loadings. Combined electrochemical and structural analyses reveal that near‐complete utilization of redox‐active sites can be achieved even in a structurally disordered polymer framework when electronic transport and redox accessibility are effectively coupled. These findings provide insight into the key factors governing utilization and kinetics in organic cathodes and offer guidance for the design of high‐performance aqueous energy storage materials.

## 2 Results and Discussion

### 2.1 Synthesis and Characterization

Imide‐linked PMDA–PTO and its small molecule analogue PhA–PTO were synthesized through a one‐step condensation reaction between 2,7‐diaminopyrene‐4,5,9,10‐tetrone (DAPTO) and either pyromellitic dianhydride (PMDA) or phthalic anhydride (PhA) (Figure 1).

**FIGURE 1:**
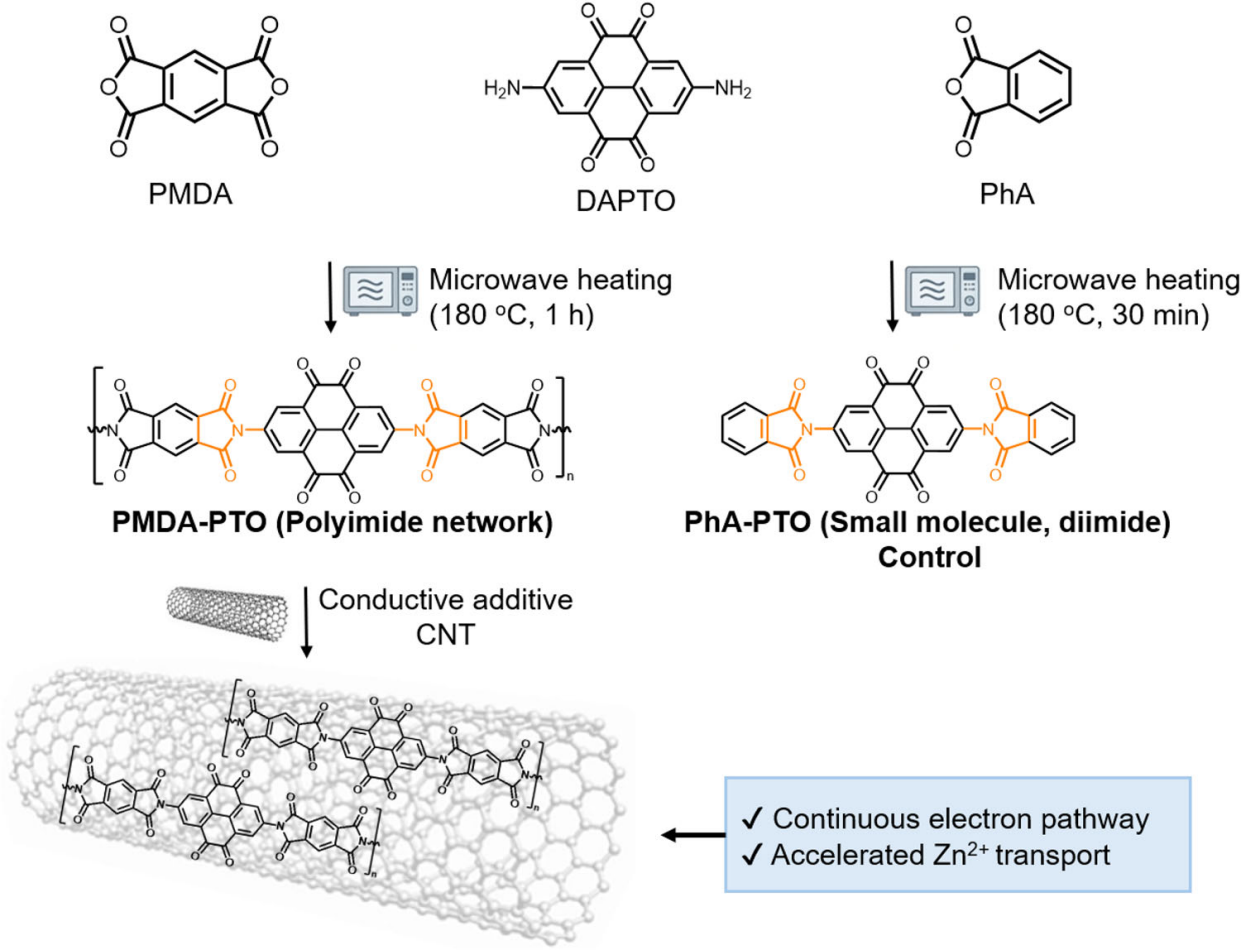
Structures of monomeric units (top). Synthetic routes to imide‐linked PMDA‐PTO, PhA‐PTO, and PMDA‐PTO‐CNT composites (middle, bottom).

Under microwave heating at 180°C, PMDA undergoes imidization with DAPTO forming a rigid π‐conjugated polyimide backbone. Reaction with PhA yields a discrete diimide model compound (PhA‐PTO) with a symmetric nonpolymeric structure. This molecular analogue serves as a control to evaluate the influence of extended conjugation and polymer connectivity on Zn^2+^ storage behavior. A PMDA–PTO–CNT composite was subsequently prepared by incorporating CNT (50 wt.% relative to the isolated PMDA–PTO yield) directly into the polymerization mixture to introduce a conductive network within the electrode architecture. Full synthetic procedures and additional characterization are provided in the Supporting Information (Figures S1–S6).

The formation of imide linkages in PMDA–PTO was confirmed by solid‐state ^13^C cross‐polarization magic‐angle spinning (CP‐MAS) nuclear magnetic resonance (NMR) and Fourier transform infrared (FTIR) spectroscopy. The ^13^C CP‐MAS NMR spectrum exhibits a distinct resonance at ∼176.9 ppm corresponding to the carbonyl (C═O) groups of the PTO core, together with a second signal at ∼167 ppm assigned to the imide carbonyls (Figure 2a) [40]. Notably, a characteristic resonance at ∼129.9 ppm is assigned to the C─N carbon within the imide ring, providing direct spectroscopic evidence for imide formation [41]. Additional resonances between 77 and 150 ppm arise from aromatic carbons of the pyrene core and the anhydride‐derived phenyl groups [42], consistent with formation of the targeted imide‐linked polymer framework. Similarly, the ^13^C CP‐MAS NMR spectrum of PhA–PTO exhibits carbonyl and aromatic resonances comparable to those of PMDA– PTO, supporting successful imide formation and generation of the expected diimide structure (Figure S7).

**FIGURE 2:**
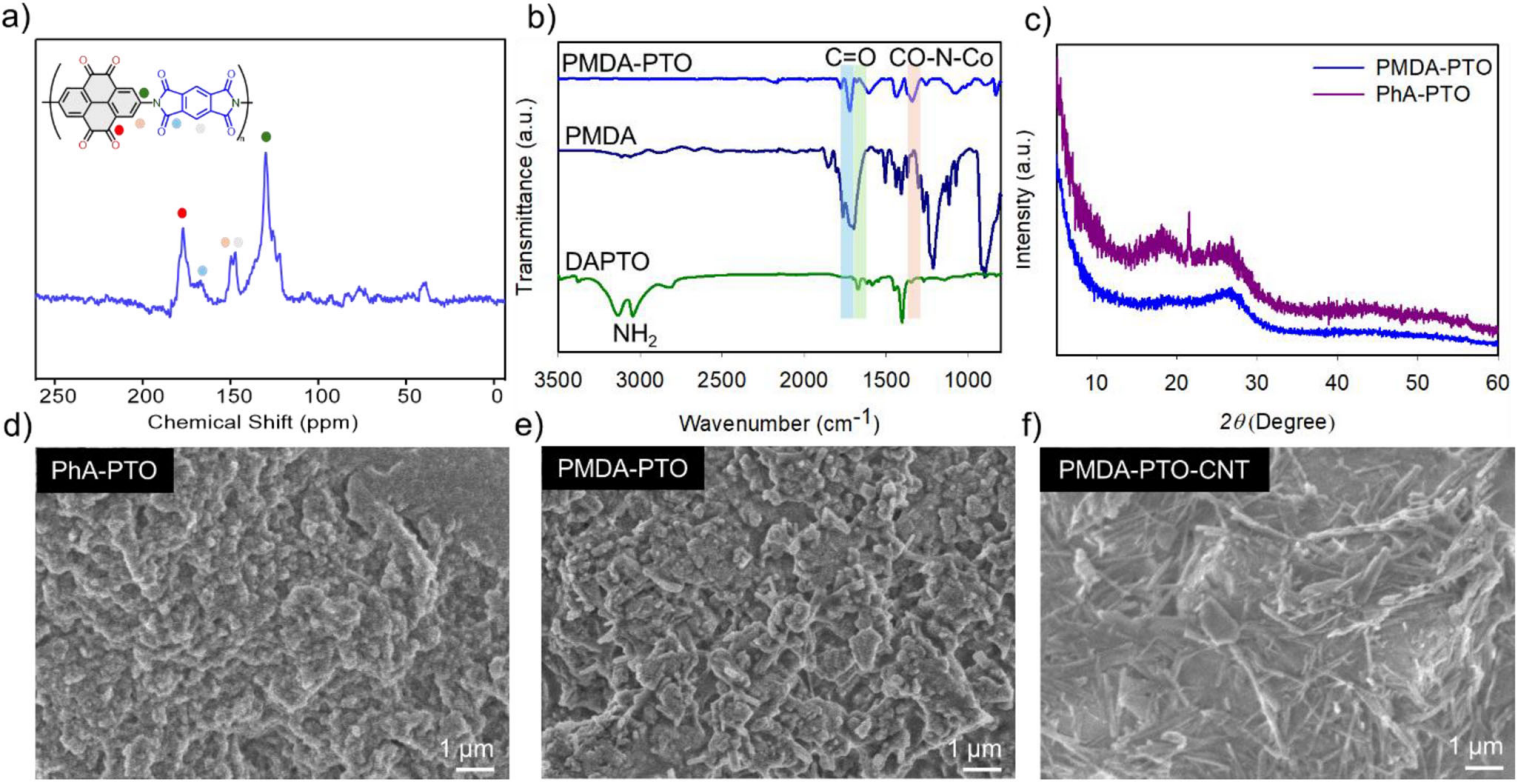
(a) Solid‐state ^13^C NMR spectrum of PMDA–PTO, (b) FTIR spectra of DAPTO, PMDA, and PMDA–PTO, (c) PXRD patterns of PhA–PTO and PMDA–PTO, and (d–f) SEM images of PhA–PTO, PMDA–PTO, and PMDA–PTO–CNT.

The FTIR spectrum of PMDA–PTO displays two strong carbonyl stretching bands at 1716 and 1672 cm^−1^, corresponding to the C═O vibrations of the imide and PTO units, respectively (Figure 2b) [43, 44]. Successful formation of the imide linkages is further confirmed by the appearance of a C─N stretching band at 1345 cm^−1^, a characteristic feature of imide‐containing structures [44, 45]. Comparison with the precursor monomers confirms complete conversion, as the N─H stretching bands of DAPTO (3145 and 3050 cm^−1^) and the anhydride C─O stretching band of PMDA (1216 cm^−1^) are absent in the spectrum of the PMDA‐PTO polymer. A similar spectral profile is observed for the small molecule analogue, PhA–PTO, which exhibits two distinct carbonyl bands together with a C─N stretching band at positions comparable to those of PMDA–PTO, accompanied by reduction of the characteristic functional groups of the starting materials (Figure S8). These results confirm successful coupling and imide formation in both products.

The powder x‐ray diffraction (PXRD) patterns of PhA–PTO and PMDA–PTO (Figure 2c) display two broad features centered at 2θ = 18.2° and 26.8°. The reflection at 26.8° is characteristic of *π*–*π* stacking interactions and corresponds to an interlayer spacing of approximately 3.3 Å, observed in both polymeric PMDA–PTO and its small molecule analogue PhA–PTO. This value is consistent with reported *π*–*π* stacking distances in related imide‐ and quinone‐containing aromatic systems [46, 47]. Although the broad diffraction profiles indicate predominantly low crystallinity, PhA–PTO exhibits an additional sharper reflection at 2θ = 21.5°, suggesting a greater degree of short‐range structural ordering in the small molecule analogue compared to the polymeric framework. In contrast, the PXRD patterns of the precursor monomers display multiple sharp reflections characteristic of highly crystalline small organic molecules (Figure S9), highlighting the substantial structural reorganization accompanying imide formation and polymerization.

CNTs were then incorporated directly into the polymerization mixture to prepare a PMDA–PTO–CNT composite containing 50 wt.% CNT. This CNT content is comparable to values commonly employed in high‐performance organic electrode composites to enhance electronic conductivity and alleviate the intrinsically low conductivity of polymeric cathodes [48, 49, 50]. The resulting composite was characterized by Raman spectroscopy to probe structural features and interactions between the polymer framework and CNTs (Figure S10). PMDA–PTO exhibits two primary Raman bands, a D' band at ∼1393 cm^−1^ and a G′ band at ∼1593 cm^−1^, corresponding to defect‐related vibrations and stretching of sp^2^‐hybridized carbon domains within the conjugated PTO–imide framework, respectively [27, 51]. Upon incorporation of CNTs, the composite displays two additional characteristic CNT bands, namely the D band at ∼1340 cm^−1^ and the G band at ∼1578 cm^−1^, associated with disordered and graphitic sp^2^ carbon networks characteristic of carbon nanotube materials [49]. The CNT reference spectrum exhibits the same D and G features, and their presence in the composite suggests incorporation of CNTs within the polymer matrix.

To further probe CNT incorporation, thermal stability, surface area and morphology were investigated to establish the interaction between the polymer and CNT components. Thermogravimetric analysis (TGA) was conducted to evaluate the thermal stability of PhA–PTO, PMDA–PTO, and the PMDA–PTO–CNT (Figure S11). All samples exhibit excellent thermal stability up to ∼250°C, with only minimal mass loss attributed to adsorbed solvent. PhA–PTO shows an earlier onset of thermal decomposition near 300°C, consistent with the lower structural rigidity of the small molecule diimide. In contrast, PMDA–PTO exhibits a more gradual mass loss beginning around 350°C, reflecting the enhanced thermal stability of the extended polyimide framework. Incorporation of CNTs preserves this high decomposition onset temperature (∼350°C) while increasing the residual mass at elevated temperatures, consistent with the intrinsic thermal robustness of CNTs [48]. These results demonstrate that polymerization significantly improves the thermal stability of PTO, while CNT incorporation further enhances the overall thermal robustness of the material.

N_2_ adsorption–desorption measurements were performed to evaluate the surface area of PMDA–PTO and the PMDA–PTO–CNT composite. PMDA–PTO exhibits negligible porosity with an extremely low Brunauer–Emmett–Teller (BET) surface area of 0.21 m^2^ g^−1^, consistent with the dense and nonporous nature of the polyimide framework. In contrast, PMDA–PTO–CNT (Figure S12) displays a significantly higher surface area of 75.0 m^2^ g^−1^, arising primarily from the intrinsically high surface area of CNTs and the presence of interparticle voids within the composite material. The increased surface area is expected to improve electrolyte access to the electrode and may facilitate utilization of redox‐active sites during electrochemical cycling.

The morphology of PhA–PTO, PMDA–PTO, and the PMDA–PTO–CNT composite was examined by scanning electron microscopy (SEM) (Figure 2d–f). PhA–PTO displays an aggregated particulate morphology composed of densely packed micron‐sized clusters, consistent with the formation of a small molecule diimide lacking extended polymer connectivity. In contrast, PMDA–PTO exhibits a sheet‐like morphology with rough and wrinkled surfaces, reflecting formation of the polyimide framework. Incorporation of CNT results in a distinct morphological transformation, with CNT features distributed throughout the polymer matrix, producing a more continuous electrode morphology. The observed morphological differences are expected to influence the electrochemical behavior of the electrodes, as discussed below.

### 2.2 Electrochemical Performance

The electrochemical performance of PhA–PTO, PMDA–PTO, and PMDA–PTO–CNT was evaluated as cathode materials for aqueous Zn‐ion storage. PMDA–PTO possesses a theoretical capacity of 315 mAh g^−1^, exceeding that of its small molecule analogue PhA–PTO (291 mAh g^−1^) due to the presence of additional redox‐active carbonyl functionalities contributed by the PMDA unit (Scheme S1). For comparison, the theoretical capacities of the PMDA (245 mAh g^−1^) and DAPTO (367 mAh g^−1^) monomers bracket that of the polymer. Although polymerization results in a modest decrease in theoretical capacity relative to DAPTO, PMDA–PTO retains a high capacity owing to the contribution of redox‐active carbonyl groups from both monomeric building blocks.

Working electrodes were prepared using active material, conductive carbon (Super P), and poly(vinylidene fluoride) (PVDF) binder in a mass ratio of 40:50:10, corresponding to active‐material loadings of 0.4–0.6 mg cm^−2^. In the case of PMDA–PTO–CNT, the active material refers to the preformed polymer–CNT composite, in which CNTs are incorporated throughout the polymer phase to enhance electronic conductivity. To assess the influence of electrode composition under more practically relevant conditions, additional electrodes containing higher active‐material fractions (70 wt.%) and increased active‐material loadings of approximately 1.0–1.3 mg cm^−2^ were also evaluated (vide infra).

Electrochemical measurements were carried out in CR2032 coin‐type cells using Zn metal as both the counter and reference electrode and 2 M ZnSO_4_ aqueous electrolyte. Galvanostatic charge–discharge and cyclic voltammetry measurements were conducted within a voltage window of 0.3–1.5 V vs. Zn/Zn^2+.^ Unless otherwise stated, specific capacities were calculated based on the mass of the active material [48, 49, 50].

Cyclic voltammetry (CV) of PMDA–PTO and PMDA–PTO–CNT was performed at 0.1 mV s^−1^ within a potential window of 0.3–1.5 V vs. Zn/Zn^2+^. The CV profile of PMDA–PTO (Figure 3a) displays two pairs of well‐defined redox peaks located at 0.65/0.97 V and 0.88/1.10 V. The lower‐potential redox couple is attributed to Zn^2+^ insertion/extraction associated with the carbonyl groups of the PMDA‐derived imide units [51, 52], whereas the higher‐potential couple corresponds to the zincation/dezincation of the carbonyl groups within the PTO core [22]. These two distinct redox processes indicate that both the imide‐ and PTO‐derived carbonyl groups participate in reversible Zn^2+^ storage.

**FIGURE 3:**
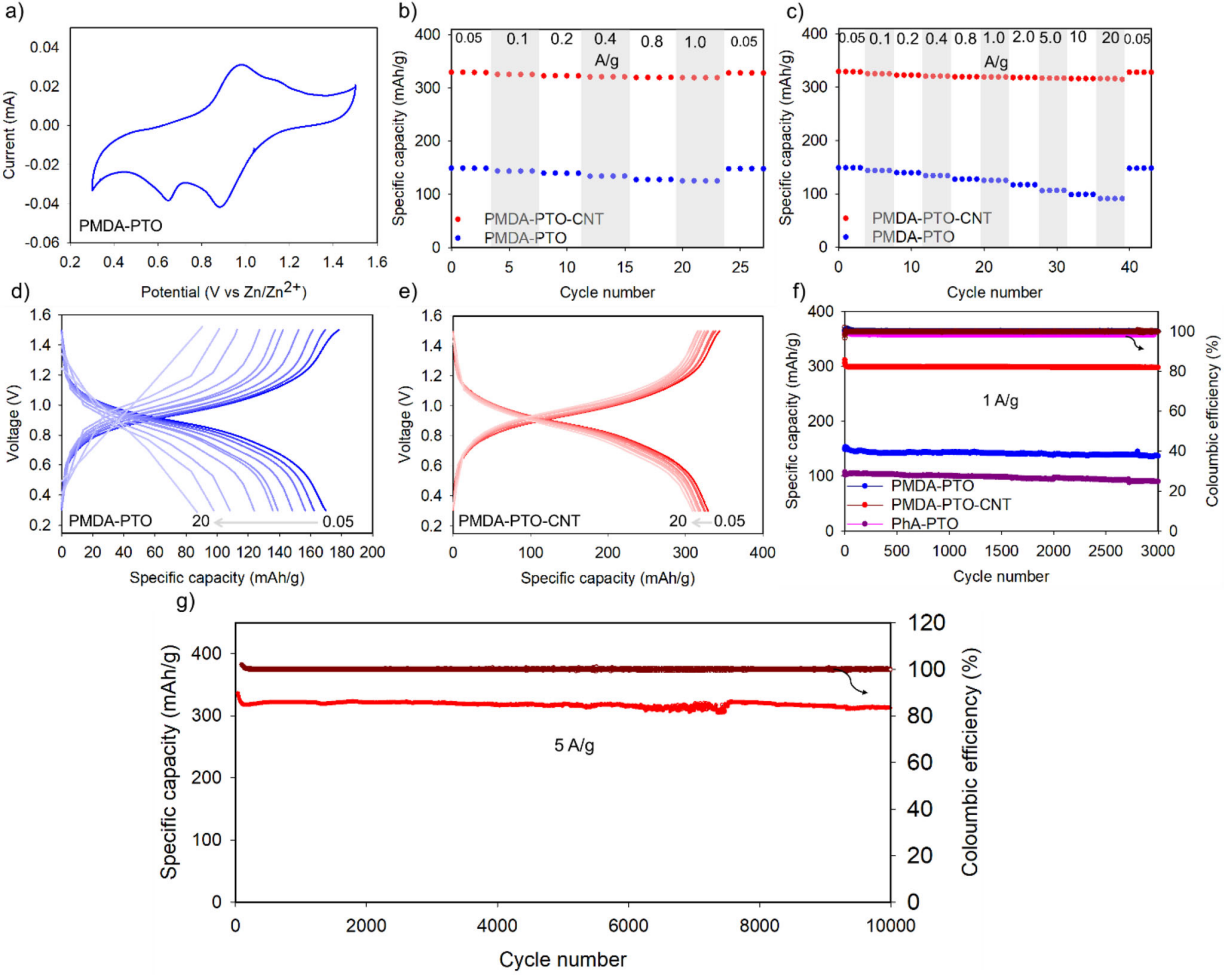
Electrochemical performance metrics of the CR2032 coin‐type ZIB half‐cells. (a) Cyclic voltammogram (CV) of PMDA–PTO at 0.1 mV s^−1^. (b,c) Rate capability of PMDA–PTO and PMDA–PTO–CNT. (d,e) Galvanostatic charge–discharge profiles of PMDA–PTO and PMDA–PTO–CNT at different current densities. (f) Cycling stability at 1 A g^−1^. (g) Long‐term cycling of PMDA–PTO–CNT at 5 A g^−1^.

The CV profile of PMDA–PTO–CNT (Figure S13) exhibits a similar redox pattern, with reduction peaks appearing at nearly identical potentials to those observed for pristine PMDA–PTO, indicating that incorporation of CNTs does not alter the fundamental redox chemistry of the polymer framework. In contrast, the oxidation region evolves into a broader anodic feature centered near ∼1.10 V, while the weaker oxidation signal near 0.88 V becomes less distinguishable due to partial overlap with the dominant oxidation process [53, 54, 55].

The rate capability of PMDA–PTO was evaluated and compared with that of the small molecule analogue PhA–PTO (Table S1 and Figure S14). At 0.05 A g^−1^, PMDA–PTO delivered a discharge capacity of 150.6 mAh g^−1^, lower than that of PhA–PTO (181.3 mAh g^−1^). The slightly higher capacity of PhA–PTO at low current density likely reflects the greater accessibility of redox‐active sites within the discrete molecular structure, whereas polymerization into PMDA–PTO improves structural integrity and is expected to reduce dissolution, though this is accompanied by some reduction in low‐rate capacity. Upon increasing the current density, however, PMDA–PTO retained a capacity of 125.7 mAh g^−1^ at 1.0 A g^−1^, whereas PhA–PTO exhibited a more pronounced capacity decay, decreasing to 77.2 mAh g^−1^ at the same current density. These results indicate that formation of the polymer framework significantly improves rate capability retention relative to the small molecule analogue. Both materials exhibited excellent reversibility, with capacities recovering close to their initial values when the current density was returned to 0.05 A g^−1^, indicating stable Zn^2+^ insertion/extraction behavior and good structural stability during cycling.

The galvanostatic charge–discharge (GCD) profiles of PhA–PTO and PMDA–PTO (Figure S15a,b) display characteristic sloping voltage plateaus associated with reversible Zn^2+^ coordination at the carbonyl groups. PhA–PTO exhibits larger discharge capacities at low current densities, consistent with the rate‐capability results discussed above, but becomes increasingly polarized at higher rates. In contrast, PMDA–PTO maintains comparatively stable and symmetric charge–discharge profiles across the investigated current range, indicating lower polarization during cycling.

Despite its stable electrochemical response, pristine PMDA–PTO exhibits relatively limited utilization of its theoretical capacity. Based on a theoretical capacity of 315 mAh g^−1^, the measured discharge capacity at 0.05 A g^−1^ corresponds to a utilization efficiency of 47.6%, indicating that a substantial fraction of the available redox‐active sites is not effectively utilized under these conditions. This observation suggests that electrochemical performance in the pristine polymer electrode is limited not by the intrinsic redox capacity of the polymer framework, but by factors related to charge transport and utilization of redox‐active sites.

Next, the electrochemical performance of the PMDA–PTO–CNT composite containing 50 wt.% CNT was evaluated. The rate capability measurements show that PMDA–PTO–CNT delivers a discharge capacity of 328 mAh g^−1^ at 0.05 A g^−1^ (Figure 3b). After accounting for the capacitive contribution of the CNT framework (Figure S16), the capacity associated with the PMDA–PTO component was estimated to be 308 mAh g^−1^ at 0.05 A g^−1^, corresponding to an estimated 97.7% utilization relative to the theoretical capacity. This substantial increase in utilization compared to pristine PMDA–PTO indicates that CNT incorporation dramatically improves electrochemical utilization of the polymer redox sites, consistent with reduced transport limitations within the electrode. In addition, the galvanostatic charge–discharge (GCD) profiles of PMDA–PTO–CNT (Figure S15c) exhibit reduced polarization and more stable discharge plateaus across a broad range of current densities relative to PMDA–PTO.

PMDA–PTO–CNT also exhibits excellent high‐rate electrochemical performance (Figure 3c), maintaining a reversible capacity of 316 mAh g^−1^ even at an ultrahigh current density of 20 A g^−1^, corresponding to charge/discharge times of approximately 1 min. When the current density is returned to 0.05 A g^−1^, the discharge capacity recovers to its initial value, indicating good reversibility and structural stability during high‐rate cycling. The ability to maintain high utilization together with stable operation over a wide current range is consistent with reduced transport limitations in the CNT‐containing electrode. In contrast, pristine PMDA–PTO exhibits more pronounced capacity decay at elevated current densities, consistent with limitations arising from the lower electronic conductivity of the polymer framework.

These transport limitations are further reflected in the GCD profiles, where increasing polarization is observed at higher current densities, manifested by a widening separation between the charge and discharge curves (Figure 3d). In comparison, polarization is substantially reduced in PMDA–PTO–CNT (Figure 3e), with the charge–discharge profiles remaining comparatively symmetric even at high rates. Together, these results indicate that CNT incorporation effectively mitigates polarization and reduces transport limitations within the polymer electrode while preserving the intrinsic redox chemistry of the polyimide.

The long‐term cycling performance of PhA–PTO, PMDA–PTO, and PMDA–PTO–CNT was evaluated at a current density of 1 A g^−1^ to assess electrochemical durability during extended cycling (Figure 3f). PhA–PTO delivers an initial discharge capacity of 107 mAh g^−1^ and retains approximately 75% of its capacity after 3000 cycles, with an average Coulombic efficiency of 98.5%. In comparison, PMDA–PTO exhibits improved cycling stability, maintaining a higher discharge capacity of 149 mAh g^−1^ with 84% capacity retention and an average Coulombic efficiency close to 99% over the same cycling period. The improved stability of PMDA–PTO relative to PhA–PTO is consistent with the enhanced structural integrity and reduced propensity for dissolution afforded by polymerization.

Most notably, PMDA–PTO–CNT maintains a reversible capacity of approximately 300 mAh g^−1^ over 3000 cycles at 1 A g^−1^ (Figure 3f), corresponding to a capacity retention of 97.5% and a Coulombic efficiency approaching 100%. These results demonstrate the excellent reversibility and durability of the electrode. Notably, this cycling test was conducted continuously over more than two months, underscoring the ability of the composite electrode to sustain high‐capacity Zn^2+^ storage over extended operation. To further evaluate the structural and electrochemical stability of the composite, extended cycling measurements were performed at a higher current density of 5 A g^−1^ (Figure 3g). Under these conditions, PMDA–PTO–CNT sustains continuous operation over 10 000 cycles while retaining a reversible capacity above 300 mAh g^−1^ with minimal capacity decay and nearly quantitative Coulombic efficiency throughout the measurement. These results demonstrate the outstanding electrochemical durability of the PMDA–PTO–CNT composite under prolonged high‐rate cycling conditions.

To further evaluate the influence of electrode composition on electrochemical behavior, PMDA–PTO–CNT electrodes containing a higher active‐material fraction (70 wt.%, active‐material loading of 1.0–1.3 mg cm^−2^) were additionally investigated (Figure 4). The rate capability measurements (Figure 4a) show that the electrode delivers a discharge capacity of approximately 270 mAh g^−1^ at 0.05 A g^−1^ and retains 242 mAh g^−1^ at 20 A g^−1^, corresponding to nearly 90% capacity retention across a 400‐fold increase in current density. The corresponding galvanostatic charge–discharge (GCD) profiles (Figure 4b) exhibit relatively symmetric charge/discharge behavior with moderate polarization even at elevated current densities, indicating preserved electrochemical reversibility under high‐rate operation. Long‐term cycling measurements performed at 5 A g^−1^ (Figure 4c) further demonstrate stable electrochemical operation, retaining 95% of the initial capacity after 1600 cycles with Coulombic efficiencies approaching 100%. Although the capacities are moderately lower than those obtained using the 40:50:10 electrode formulation, the preserved high‐rate performance and cycling stability demonstrate that the PMDA–PTO framework remains highly electrochemically active at increased active‐material fractions and loadings. These results further indicate that the conductive CNT network continues to effectively support charge transport within the electrode under more practically relevant conditions. Compared with previously reported polymer‐based organic cathodes for aqueous Zn‐ion batteries, PMDA–PTO–CNT demonstrates competitive rate capability and long‐term cycling stability, particularly under increased active‐material loading and ultrahigh‐rate operation (Table S2) [19, 21, 22, 25, 27, 52, 56, 57, 58, 59].

**FIGURE 4:**
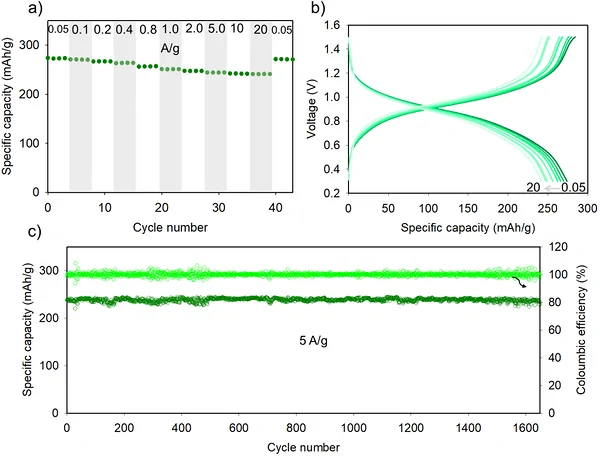
Electrochemical performance of PMDA–PTO–CNT electrodes containing a higher active‐material fraction (70 wt.%, active‐material loading: 1.0–1.3 mg cm^−2^). (a) Rate capability at different current densities. (b) Galvanostatic charge–discharge (GCD) profiles collected at various current densities. (c) Long‐term cycling performance and Coulombic efficiency measured at 5 A g^−1^.

The rate capability of a PMDA–PTO–CNT electrode prepared without additional Super P conductive carbon was evaluated to further examine the transport characteristics of the composite electrode (Figure S17). The electrode delivers a reversible capacity of approximately 125 mAh g^−1^ at 0.05 A g^−1^, indicating significantly reduced utilization relative to the optimized electrode formulation. Despite the lower capacity, the observed electrochemical activity confirms that the CNT‐containing polymer framework remains capable of reversible Zn^2+^ storage in the absence of added conductive carbon. However, the capacity decreases substantially with increasing current density, reaching ∼25 mAh g^−1^ at 1.0 A g^−1^, indicating pronounced transport limitations under high‐rate conditions. Notably, when the current density is returned to 0.05 A g^−1^, the capacity largely recovers, suggesting that the reduced high‐rate performance primarily arises from reversible polarization rather than irreversible structural degradation. Although the PMDA–PTO–CNT composite contains 50 wt.% CNT, the CNT and Super P serve complementary rather than redundant functions in constructing the conductive network. The CNT establish a continuous conductive framework within the polymer composite, whereas Super P enhances electrical contact between composite particles throughout the electrode and reduces interparticle resistance. These results demonstrate that the high specific capacity and excellent rate performance of PMDA–PTO–CNT arise from efficient electrochemical utilization of the redox‐active polymer enabled by the complementary roles of the CNT framework and Super P, rather than from either conductive component acting independently.

### 2.3 Charge Storage Mechanism

Ex situ FTIR spectroscopy was employed to investigate the structural evolution of PhA–PTO, PMDA–PTO, and PMDA–PTO–CNT during electrochemical cycling (Figure S18). For all three electrodes, the pristine spectra display characteristic C═O stretching vibrations in the 1650–1750 cm^−1^ region, corresponding to the carbonyl groups of the PTO and imide moieties. Upon discharge, noticeable attenuation of these bands is observed, consistent with coordination of charge‐compensating cations to the carbonyl functionalities during the reduction process. Subsequent charging restores the C═O stretching features to their original positions and intensities, indicating reversible ion insertion/extraction without permanent chemical transformation of the electrode materials. These results support a reversible carbonyl‐centered redox mechanism in all systems and further indicate that incorporation of CNTs does not alter the fundamental electrochemical reaction chemistry of the polymer framework.

In line with the ex situ FTIR analysis, ex situ x‐ray photoelectron spectroscopy (XPS) was employed to further probe the chemical environment and zinc‐coordination behavior of PMDA–PTO–CNT during electrochemical cycling. The XPS C 1s spectra collected at the charged and discharged states (Figure 5a) can be deconvoluted into four components located at 284.6 eV (C─C/C═C), 286.2 eV (C─N), 287.4 eV (C─O), and 288.6 eV corresponding to carbonyl carbon (C═O), consistent with the imide‐linked PTO framework [60]. In addition, the signal at ∼291 eV is assigned to a *π*–*π* ^*^ shake‐up satellite characteristic of extended π‐conjugated aromatic systems [60]. Similarly, the N 1s spectra (Figure S19) exhibit two dominant peaks centered at ∼400.2 and ∼399.5 eV, attributed to conjugated C─N and imide ─OC─N─CO─ environments, respectively [61], confirming preservation of the imide backbone during electrochemical cycling. No significant shifts in the C 1s or N 1s binding energies are observed between charged and discharged states, indicating that the polymer framework remains chemically stable throughout the Zn^2+^ insertion/extraction process.

**FIGURE 5:**
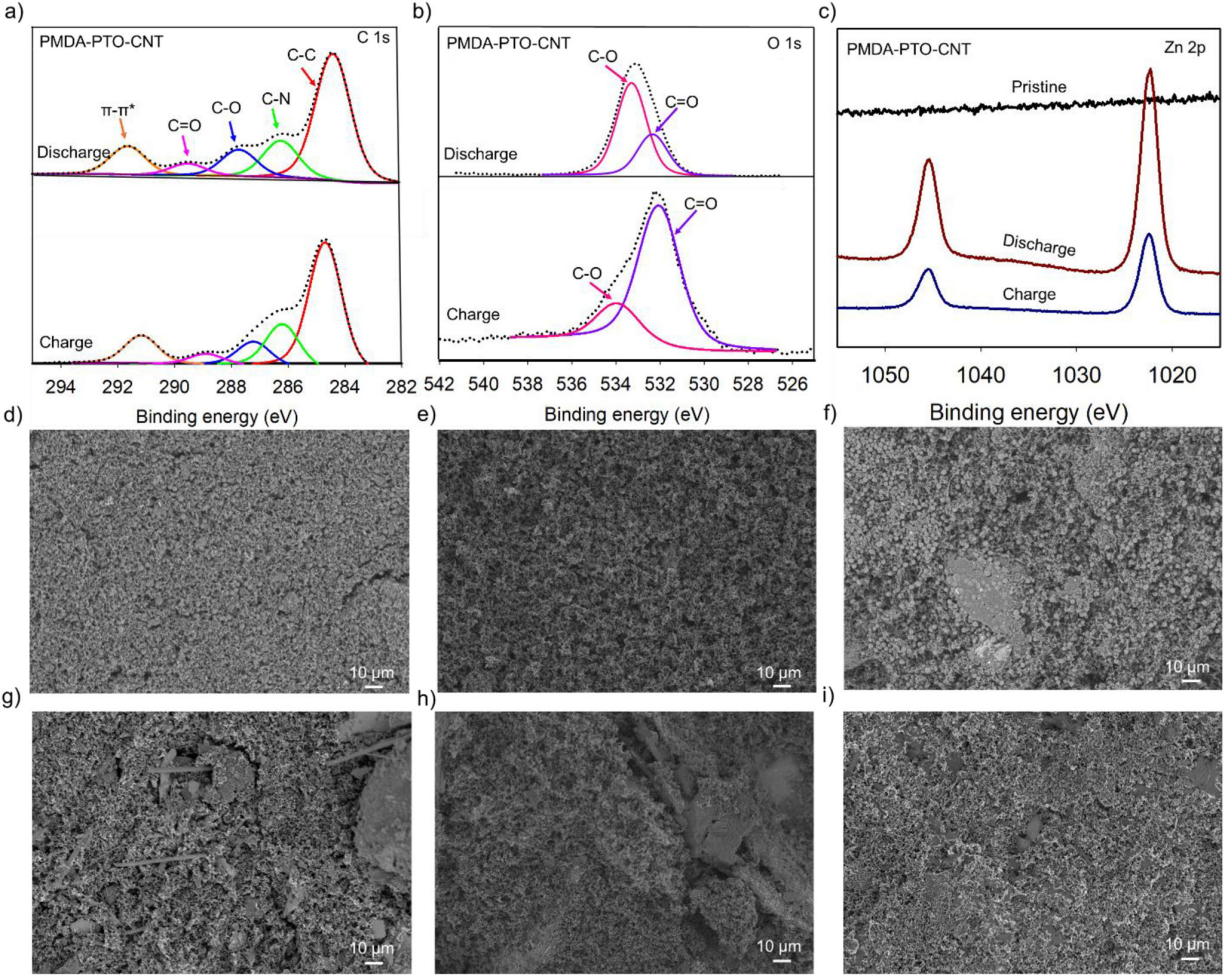
Materials characterization of the CNT composites. Ex situ XPS spectra of PMDA–PTO–CNT at different electrochemical states: (a) C 1s, (b) O 1s, and (c) Zn 2p. SEM images of electrodes in the pristine state and after 3000 cycles for PhA–PTO (d,g), PMDA–PTO (e,h), and PMDA–PTO–CNT (f,i).

In contrast, the O 1s spectra (Figure 5b) exhibit clear spectral evolution during cycling. Upon discharge, the relative intensity of the carbonyl‐related component (∼532.0 eV) decreases, accompanied by an increased contribution from higher‐binding‐energy C─O species (∼533–534 eV). This behavior is consistent with electron‐density redistribution associated with reversible zinc coordination at oxygen‐containing groups rather than irreversible chemical transformation. Upon recharging to 1.5 V, the O 1s spectral profile largely recovers to its initial state, supporting the reversibility of the coordination process.

Consistently, the Zn 2p spectra (Figure 5c) display pronounced signals in the discharged electrode that become substantially attenuated after charging, confirming reversible Zn^2+^ insertion/extraction during cycling. A weak residual Zn signal remains after charging, suggesting minor Zn retention within the electrode, a phenomenon commonly observed in aqueous Zn‐ion systems [52]. Comparable XPS behavior is observed for PMDA–PTO and PhA–PTO (Figures S20 and S21), supporting a common carbonyl‐centered Zn^2+^ storage mechanism across all materials. Collectively, the FTIR and XPS analyses support a predominantly carbonyl‐centered Zn^2+^ storage mechanism involving reversible cation coordination at the oxygen sites, although minor proton‐assisted contributions under aqueous operating conditions cannot be completely excluded.

SEM was employed to examine the morphological evolution of PhA–PTO, PMDA–PTO, and PMDA–PTO–CNT electrodes before cycling and after prolonged electrochemical operation (Figure 5d–i). In the pristine state, all electrodes exhibit relatively uniform morphologies, indicating good film formation on the current collector (Figure 5d–f). After 3000 cycles, the PhA–PTO electrode (Figure 5g) exhibits pronounced morphological changes, including particle agglomeration, surface cracking, and fragmentation. In comparison, PMDA–PTO (Figure 5h) retains a more intact morphology, although localized cracking and surface deterioration are still observed. The improved morphological retention of PMDA–PTO is consistent with the enhanced structural stability imparted by polymerization. Among the three materials, PMDA–PTO–CNT exhibits the smallest morphological changes after cycling (Figure 5i), with no obvious large‐scale pulverization observed. Higher‐magnification SEM images collected before and after cycling are provided in Figure S22 and further illustrate the comparatively preserved morphology of PMDA–PTO–CNT relative to pristine PMDA–PTO.

SEM images collected after high‐rate capability measurements up to 20 A g^−^^1^ are shown in Figure S23. Following high‐rate cycling, PMDA–PTO exhibits localized agglomeration and morphological deterioration. In contrast, PMDA–PTO–CNT displays comparatively smaller morphological changes after cycling, indicating improved structural retention under high‐rate operating conditions. These observations are consistent with the enhanced electrochemical durability of PMDA–PTO–CNT.

The Zn^2+^ storage kinetics were further investigated by cyclic voltammetry performed at different scan rates for PMDA–PTO and PMDA–PTO–CNT (Figure 6a,b). The relationship between peak current (i) and scan rate (ν) follows the power‐law equation *i*  =  *a*ν^*b*^, where the exponent b provides insight into the dominant charge‐storage mechanism. A b value approaching 0.5 indicates diffusion‐controlled behavior, whereas a value approaching 1.0 corresponds to surface‐controlled (pseudocapacitive) charge storage [62]. Log–log plots of peak current vs. scan rate (Figure S24) reveal that PMDA–PTO exhibits b values of 0.65 and 0.75 for the two cathodic peaks and 0.84 for the anodic peak, indicating mixed diffusion‐ and capacitive‐controlled charge‐storage behavior. In contrast, PMDA–PTO–CNT displays higher b values of 0.93 and 0.90 for the corresponding cathodic peaks and 0.88 for the anodic peak, suggesting a substantially greater pseudocapacitive contribution in the CNT‐integrated composite. These results indicate that CNT incorporation facilitates faster electrochemical kinetics and improves accessibility of redox‐active sites within the polymer framework.

**FIGURE 6:**
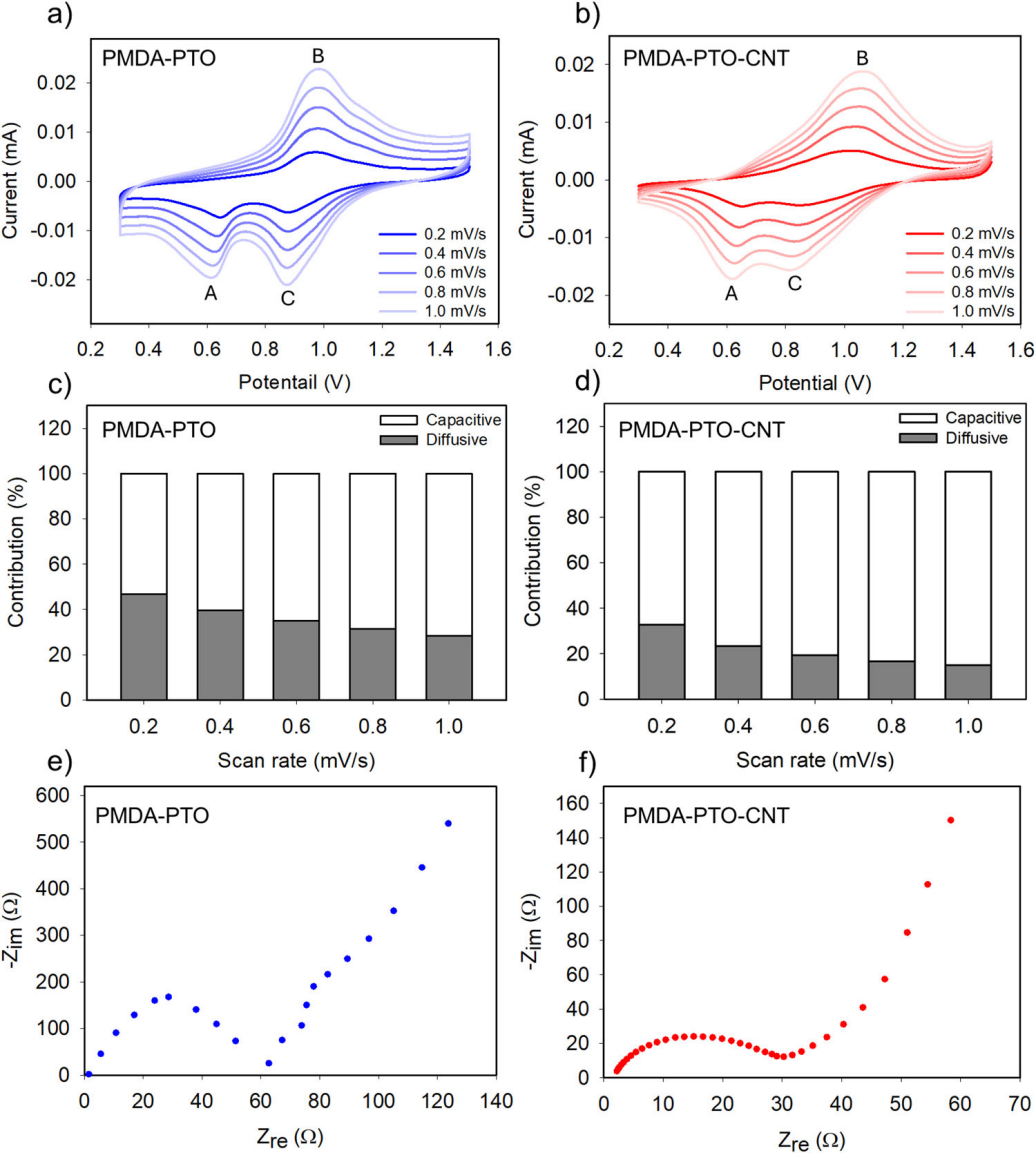
Mechanistic insights into Zn^2+^ storage kinetics in PMDA–PTO and PMDA–PTO–CNT electrodes. Cyclic voltammograms of (a) PMDA–PTO and (b) PMDA–PTO–CNT recorded at different scan rates. Quantitative analysis of capacitive and diffusion‐controlled contributions for the selected reduction peak of (c) PMDA–PTO and (d) PMDA–PTO–CNT. Nyquist plots of (e) PMDA–PTO and (f) PMDA–PTO–CNT obtained by electrochemical impedance spectroscopy measured at open‐circuit voltage (OCV) after 10 cycles.

To further distinguish capacitive and diffusion‐controlled contributions, Dunn's method was applied by analyzing the current response at fixed potentials over different scan rates. Linear fitting was used to extract the k_1_ and k_2_ parameters corresponding to capacitive and diffusion‐controlled processes, respectively, allowing quantification of the capacitive contribution at the cathodic peak (Figure 6c,d). For both electrodes, the capacitive contribution increases with scan rate, consistent with the progressively reduced contribution from long‐range ion diffusion at higher sweep rates. Specifically, the capacitive contribution of PMDA–PTO increases from 53% at 0.2 mV s^−1^ to 72% at 1.0 mV s^−1^. In comparison, PMDA–PTO–CNT exhibits consistently higher capacitive contributions, increasing from 67% to 85% over the same scan‐rate range. The enhanced pseudocapacitive response of PMDA–PTO–CNT suggests improved electrochemical kinetics within the composite electrode, consistent with the superior rate performance observed during galvanostatic cycling [63, 64].

Electrochemical impedance spectroscopy (EIS) was employed to further investigate the charge‐transfer and Zn^2+^ transport characteristics of PMDA–PTO and PMDA–PTO–CNT electrodes (Figure 6e,f). The Nyquist plots reveal a substantially smaller semicircle for PMDA–PTO–CNT compared with pristine PMDA–PTO, indicating reduced charge‐transfer resistance at the electrode/electrolyte interface. In addition, the overall impedance response is significantly decreased across the measured frequency range. These observations are consistent with improved electrochemical kinetics and more efficient charge transport in the CNT‐containing electrode. In the low‐frequency region, the linear dependence of Z′ on ω^−1/2^ reflects Warburg diffusion associated with ion transport within the electrode (Figure S25). PMDA–PTO–CNT exhibits a significantly smaller Warburg coefficient (σ ≈ 10 Ω s^1/2^) compared to pristine PMDA–PTO (σ ≈ 33 Ω s^1/2^), corresponding to calculated Zn^2+^ diffusion coefficients of ∼5.4 × 10^−14^ and ∼4.9 × 10^−15^ cm^2^ s^−1^, respectively (Table S3). These values are consistent with Zn^2+^ diffusion coefficients previously reported for related organic polymer cathodes [21, 25, 65]. The substantially reduced transport resistance observed for PMDA–PTO–CNT indicates improved charge‐transfer characteristics within the composite electrode. This observation is consistent with the enhanced rate capability, reduced polarization, and increased pseudocapacitive contribution observed for the CNT‐containing electrode.

GIWAXS was employed to investigate the structural origins of the fast Zn^2+^ storage behavior and enhanced electrochemical transport in the composite electrodes (Figure 7; Figure S26) [66, 67, 68, 69]. Across all samples, the GIWAXS patterns are dominated by broad scattering features rather than sharp Bragg reflections, indicating the absence of long‐range crystallinity. These observations suggest that the electrochemical performance does not arise from highly ordered polymer domains, but instead from short‐range molecular organization and electrode‐level structural correlations.

**FIGURE 7:**
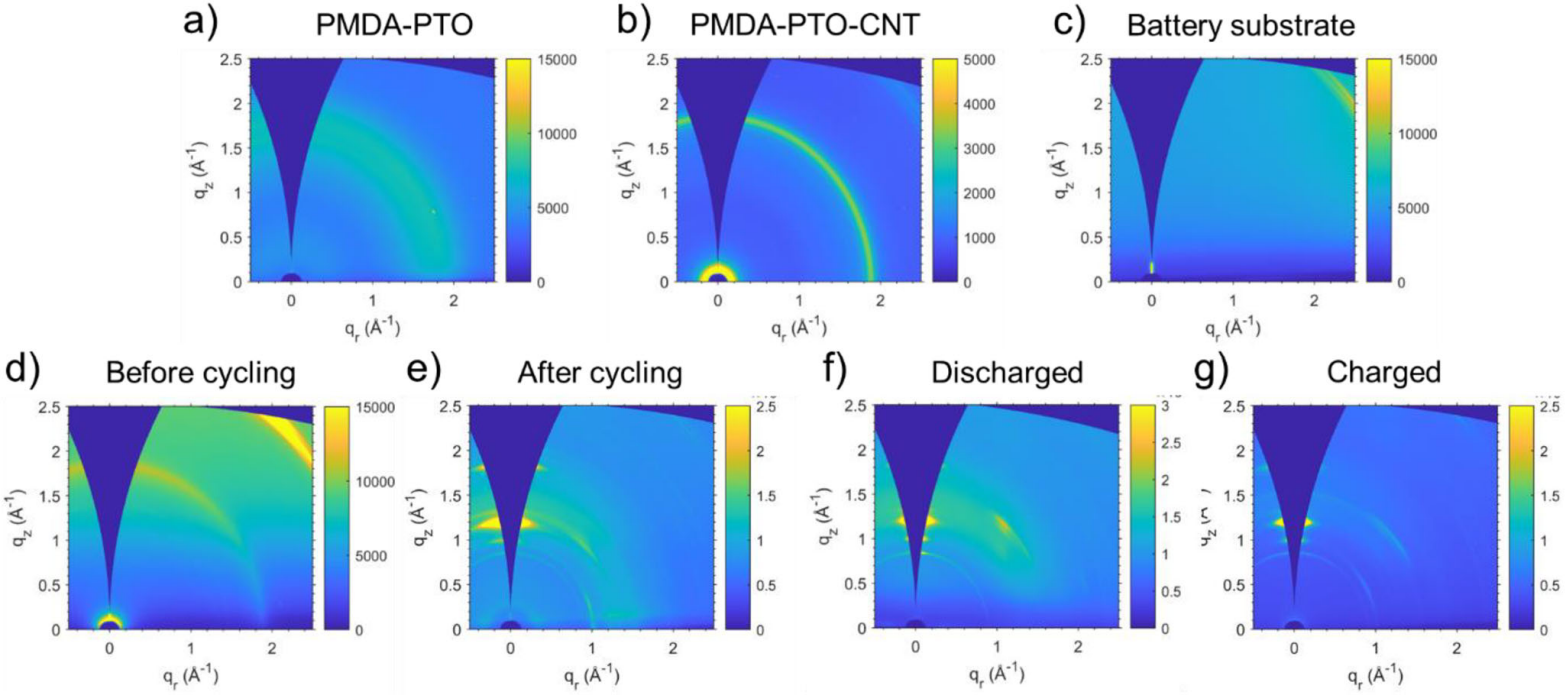
Two‐dimensional GIWAXS patterns of the (a) PMDA‐PTO, (b) PMDA‐PTO‐CNT, (c) bare substrate (stainless steel foil), and assembled electrodes measured (d) before cycling, (e) after extended electrochemical cycling, (f) in the discharged, and (g) charged states.

The pristine PMDA–PTO electrode exhibits a broad scattering feature centered at q ≈ 1.50 Å^−1^ (Figure 7a), corresponding to a real‐space correlation length of ∼4.2 Å. The broad feature and absence of higher‐order reflections indicate limited coherence lengths associated with local *π*–*π*‐like correlations rather than extended crystalline stacking. Furthermore, the nearly isotropic intensity distribution observed in the 2D pattern suggests the absence of strong preferential orientation within the polymer electrode [70].

Incorporation of CNTs significantly modifies the scattering response. The PMDA–PTO–CNT composite exhibits enhanced scattering intensity together with partial anisotropy within a similar q range, with arc‐like features indicative of textured structural organization (Figure 7b). Importantly, the dominant scattering feature remains centered near q ≈ 1.50 Å^−1^, indicating that CNT incorporation does not induce polymer crystallization or substantially alter the intrinsic packing length scale of the polymer framework. Instead, the observed anisotropy likely originates from the oriented CNT network, which may facilitate electronic transport throughout the composite electrode [49, 71]. This interpretation is consistent with the reduced polarization and enhanced electrochemical kinetics observed electrochemically.

The bare substrate exhibits no discernible scattering features within the relevant q range (Figure 7c), confirming that the observed GIWAXS signals originate from the active electrode components. Electrode fabrication prior to cycling results in increased scattering intensity and weak anisotropy relative to the pristine polymer, suggesting mesoscale structural reorganization during slurry casting and electrode densification (Figure 7d). More pronounced structural evolution occurs during electrochemical cycling (Figure 7e). In the discharged state, a new broad scattering feature emerges at q ≈ 1.20–1.22 Å^−1^, corresponding to a spacing of ∼5.1–5.3 Å (Figure 7f). This feature is weak or absent in the pristine and before‐cycling electrodes, indicating the development of an additional short‐range structural correlation upon electrochemical insertion of charge‐compensating cations.

The associated scattering appears as localized arcs rather than a continuous ring, suggesting partial directional organization within the cycled electrode. Although the precise structural origin of the ∼5.2 Å feature cannot be assigned unambiguously from the present data, its reversible evolution with electrochemical state suggests that it is associated with local structural reorganization during Zn^2+^ insertion and extraction. Possible contributions from electrolyte‐associated species or interfacial ordering effects cannot be completely excluded. Importantly, the feature remains broad and does not evolve into sharp Bragg reflections after extended cycling (Figure 7g), indicating the absence of long‐range crystallization or irreversible phase transformation.

The GIWAXS results reveal a clear decoupling between long‐range structural order and electrochemical performance. Rapid Zn^2+^ storage is achieved within a structurally disordered polymer framework that retains short‐range organization throughout cycling, while the CNT‐integrated architecture promotes efficient electrode‐level transport pathways. Together, these observations indicate that the PMDA–PTO–CNT composite maintains short‐range structural organization during repeated Zn^2+^ insertion and extraction, which may contribute to its fast and durable Zn^2+^ storage performance [17, 72, 73].

It should be noted that proton participation has been reported to accompany Zn^2+^ storage in aqueous ZnSO_4_ electrolytes for certain organic cathodes [74, 75]. The electrochemical, spectroscopic, and structural analyses presented here are consistent with reversible cation storage involving the carbonyl functionalities of the PMDA–PTO framework. However, the present measurements do not explicitly distinguish between Zn^2+^ and H^+^ participation. Therefore, while the results are consistent with dominant Zn^2+^ storage, a minor contribution from proton‐assisted redox processes cannot be completely excluded under the present aqueous operating conditions.

To gain molecular‐level insight into the redox behavior of PMDA–PTO, density functional theory (DFT) calculations were performed using a representative oligomeric model of the polymer backbone (see Computational Details, Section S7). The calculations were designed to probe the intrinsic electronic structure and reduction behavior of the conjugated PMDA–PTO framework, enabling evaluation of charge delocalization and accessible redox states within the polymer backbone. While the Zn^2+^ storage mechanism was experimentally investigated through ex situ FTIR and XPS analyses, the DFT calculations provide complementary insight into the electronic evolution of the carbonyl‐containing conjugated framework upon reduction.

The calculations predict two thermodynamically accessible reduction steps per repeat unit, consistent with the two cathodic peaks observed experimentally in the cyclic voltammograms. The first reduction corresponds to formation of a delocalized radical anion, whereas the second generates a doubly reduced state stabilized in a triplet configuration (Table S4), enabled by extensive π‐conjugation across the diimide–tetraketone backbone. The calculated free‐energy change associated with the second electron insertion is slightly more favorable than that of the first (Table S5), rationalizing the close spacing of the experimentally observed reduction waves and supporting efficient multielectron storage within the conjugated framework.

In contrast, the oxidation processes are calculated to occur at higher and closely spaced energies, consistent with the experimentally observed broad anodic response rather than two well‐resolved oxidation peaks. Frontier molecular orbital analysis further reveals that the LUMO is delocalized across both the imide and PTO units of the polymer backbone (Figure S27), supporting carbonyl‐centered electron uptake and extensive charge delocalization upon reduction. At the same time, the relatively large calculated HOMO–LUMO gap suggests intrinsically limited electronic conductivity in the pristine polymer, consistent with the experimentally observed transport limitations of PMDA–PTO. The enhanced electrochemical performance observed in PMDA–PTO–CNT therefore likely arises from improved electronic percolation and reduced transport limitations within the composite architecture rather than changes in the intrinsic redox chemistry of the polymer backbone. The DFT results provide molecular‐level insight into the multistep electrochemical behavior of PMDA–PTO and support the experimentally observed carbonyl‐centered redox activity and extensive charge delocalization within the conjugated polymer framework.

## 3 Conclusions

In summary, a π‐conjugated imide‐linked polyimide cathode based on pyrene‐4,5,9,10‐tetraone and pyromellitic dianhydride (PMDA–PTO) was developed through a straightforward microwave‐assisted synthesis. Integration of the polymer framework with CNTs produces a composite electrode capable of achieving high utilization efficiency together with excellent rate capability and long‐term cycling stability in aqueous Zn‐ion batteries. The PMDA–PTO–CNT electrode delivers a discharge capacity of 328 mAh g^−1^ at 0.05 A g^−1^, corresponding to ∼98% utilization of the theoretical capacity, while maintaining stable electrochemical operation at current densities up to 20 A g^−1^ and over 10 000 cycles.

Comprehensive electrochemical and structural analyses reveal that the enhanced electrochemical behavior arises from improved electronic percolation and more efficient utilization of redox‐active sites within the CNT‐integrated architecture. Kinetic analyses demonstrate predominantly pseudocapacitive charge‐storage behavior in the composite electrode, accompanied by reduced polarization and improved electrochemical transport kinetics. Ex situ FTIR and XPS measurements support a reversible carbonyl‐centered cation‐storage mechanism, while GIWAXS measurements reveal that fast electrochemical transport can be achieved within a structurally disordered polymer framework that retains only short‐range molecular organization. DFT calculations further support the experimentally observed multistep redox behavior and extensive charge delocalization within the conjugated backbone.

These findings provide insight into the relationship between structural disorder, electronic percolation, and redox‐site utilization in quinone‐based polymer cathodes. More broadly, this work highlights the importance of coupling redox‐active polymer frameworks with conductive transport networks to achieve high utilization, fast kinetics, and long‐term stability in aqueous Zn‐ion batteries and provides molecular and electrode‐design principles for the development of high‐performance organic cathodes for aqueous and multivalent battery systems.

## 4 Experimental Section

### 4.1 Materials

All chemicals were purchased from commercial suppliers and used as received unless otherwise noted. Multi‐walled carbon nanotubes (MWCNT, outer diameter 30–50 nm, length 10–20 µm) were obtained from Cheap Tubes Inc. Full reagent details are provided in the Supporting Information.

### 4.2 Characterization

Structural and compositional characterization was performed using solution and solid‐state NMR, FTIR, Raman spectroscopy, PXRD, XPS, SEM, TGA, and nitrogen sorption measurements. GIWAXS experiments were conducted at the Canadian Light Source. Detailed instrumental parameters and data analysis procedures are provided in the Supporting Information.

### 4.3 Synthesis of PMDA–PTO

PMDA–PTO was synthesized via a microwave‐assisted polycondensation between diaminopyrene‐4,5,9,10‐tetraone (DAPTO) and pyromellitic dianhydride (PMDA) in anhydrous DMF at 180°C for 1 h. The resulting polymer was precipitated in methanol, washed thoroughly, and dried under vacuum to afford a black solid (80% yield). Detailed synthetic procedures are provided in the Supporting Information.

### 4.4 Synthesis of PMDA–PTO–CNT

PMDA–PTO–CNT composites were prepared using a similar microwave protocol, with CNTs introduced during polymerization to achieve a target loading of 50 wt.%. The composite was isolated by precipitation, washing, and vacuum drying. Full experimental details are described in the Supporting Information.

### 4.5 Electrode Preparation and Electrochemical Measurements

Working electrodes were fabricated by casting a slurry of active material, Super P, and PVDF binder (40:50:10 wt.%) onto stainless‐steel foil and drying overnight. Electrochemical performance was evaluated in CR2032 coin cells using Zn foil as the anode and 2 m ZnSO_4_ aqueous electrolyte. Galvanostatic charge–discharge, cyclic voltammetry, and electrochemical impedance spectroscopy were performed within a voltage window of 0.3–1.5 V (vs. Zn/Zn^2+^). Additional experimental parameters are provided in the Supporting Information.

### 4.6 Computational Details

Density functional theory (DFT) calculations were carried out using Gaussian 16 to investigate the redox behavior of PMDA–PTO. Geometry optimizations and frequency calculations were performed using the ωB97X‐D functional with the def2‐TZVP basis set and implicit solvation (SMD, water). Full computational details are described in the Supporting Information.

## Author Contributions

**Heba H. Farrag**: conceptualization, methodology, investigation, formal analysis, writing – original draft, writing – review and editing, validation, visualization, data curation. **Jônatas Faleiro Berbigier**: methodology, formal analysis, visualization, writing – review and editing. **Ailsa K. Edward**: investigation, Writing – review and editing. **Dwight S. Seferos**: supervision, funding acquisition, writing – review and editing, resources, project administration.

## Conflicts of Interest

The authors declare no conflicts of interest.

## Supporting information




**Supporting File**: smll75112‐sup‐0001‐SuppMat.pdf.

## Data Availability

The data that support the findings of this study are available in the text and supplementary materials of this article.
