# Supplementary material for: Near‐Theoretical Redox‐Site Utilization and Fast Zn2+ Storage in a Polyimide‐CNT Cathode
Source: Small. 2026 Aug 8;22(57):e75112. doi: 10.1002/smll.75112 (PMC13659785; doi:10.1002/smll.75112)
Supplement: Supplementary file 1 [file SMLL-22-e75112-s001.pdf]

Supporting Information

Near-Theoretical Redox-Site Utilization and Fast Zn^{2+} Storage in a Polyimide–CNT Cathode

Heba H. Farrag[1,2*], Jônatas Faleiro Berbigier[1], Ailsa K. Edward[1], and Dwight S. Seferos[1,3]

[1]Department of Chemistry, University of Toronto, Lash Miller Chemical Laboratories, 80 St. George Street, Toronto, Ontario, M5S 3H6, Canada

[2]Department of Chemistry, Faculty of Science, Cairo University, Cairo 12613, Egypt

[3]Department of Chemical Engineering and Applied Chemistry, University of Toronto, 200 College Street, Toronto, Ontario, M5S 3E5, Canada

**Corresponding authors. Email address:* heba.farrag@mail.utoronto.ca

Contents

1. Materials and methods S3

2. Synthesis and characterization S4

3. Calculation of theoretical capacity S17

4. Electrochemical measurements S18

5. Post-cycling characterization S27

6. Mechanistic studies S33

7. Computational details S38

8. References S41

1. Materials and methods

Pyrene (≥99%), sodium periodate (≥99.8%), ruthenium(III) chloride hydrate (≥99.9%), dichloromethane (DCM), sodium bicarbonate (≥99.7%), sodium hydrosulfite, sodium hydroxide (≥97%), ammonium chloride (≥99.5%), 2,3-dichloro-5,6-dicyano-p-benzoquinone (DDQ), methanol (anhydrous, ≥99.8%), pyromellitic dianhydride (99.9%), phthalic anhydride (≥99%), dimethyl sulfoxide (DMSO, anhydrous, ≥99.9%), dimethylformamide (DMF), poly(vinylidene fluoride) (PVDF), and zinc sulfate heptahydrate (99.5%) were purchased from Sigma-Aldrich and used as received. Fuming nitric acid (98%) and sulfuric acid (reagent grade) were obtained from Fisher Scientific. All other solvents were purchased from Sigma-Aldrich and used without further purification. Multi-walled carbon nanotubes (MWCNT, ≥95% purity; outer diameter 30–50 nm; length 10–20 μm) were purchased from Cheap Tubes Inc.

Solution NMR spectra were recorded at room temperature using either a 400 MHz Bruker Avance III or a 600 MHz Agilent DD2 spectrometer, with chemical shifts referenced to the residual DMSO signal.

Solid-state ^{13}C cross-polarization magic-angle spinning (CP-MAS) NMR spectra were acquired on a 700 MHz Agilent DD2 spectrometer, using adamantane as an external reference.

Fourier transform infrared (FTIR) spectra were collected on a Thermo Scientific iS50 spectrometer.

Raman spectra were obtained using a Bruker SENTERRA dispersive Raman microscope.

High-resolution mass spectra were recorded on a JEOL AccuTOF Plus 5G (JMS-T1000LP) mass spectrometer equipped with a direct analysis in real time (DART) ion source.

Gas adsorption and desorption data were acquired using a Micromeritics Tristar II Plus at 77 K with nitrogen gas. Prior to measurement, a sample was activated under a vacuum at 100 ° C for 20 h on a Micromeritic Smart VacPrep instrument equipped with a turbo pump. Specific surface areas were calculated using the Brunauer–Emmett–Teller (BET) method in the relative pressure range of $0.05 < P/P_0 < 0.30$.

Thermogravimetric analysis (TGA) was performed on a Netzsch STA 449 F3 Jupiter instrument under an argon atmosphere (50 mL min^{-1}) with a heating rate of 10 °C min^{-1}.

Powder X-ray diffraction (PXRD) patterns were collected using a Rigaku MiniFlex 600 benchtop diffractometer with Cu Kα radiation (λ = 1.5406 Å).

X-ray photoelectron spectroscopy (XPS) measurements were conducted on a Thermo Scientific K-Alpha spectrometer equipped with a monochromatic Al Kα source.

Scanning electron microscopy (SEM) images were acquired using a Hitachi SU7000 SEM operating at an accelerating voltage of 1.5 kV.

Grazing-Incidence Wide-Angle X-ray Scattering (GIWAXS) measurements were performed at the BXDS sector of the Canadian Light Source using monochromatic X-rays (9.8713 keV, λ = 1.25602 Å) selected by a Si(111) double-crystal monochromator. Data were collected in reflection geometry under a helium atmosphere at an incident angle of 0.5° with 5 s exposure time. Scattering patterns were recorded on a 4096 × 4096 pixel area detector at a sample-to-detector distance of 251.95 mm. Data processing and reciprocal-space mapping were conducted using the GIXSGUI MATLAB toolbox.[S1]

2. Synthesis and characterization

Synthesis of pyrene-4,5,9,10-tetraone (PTO)

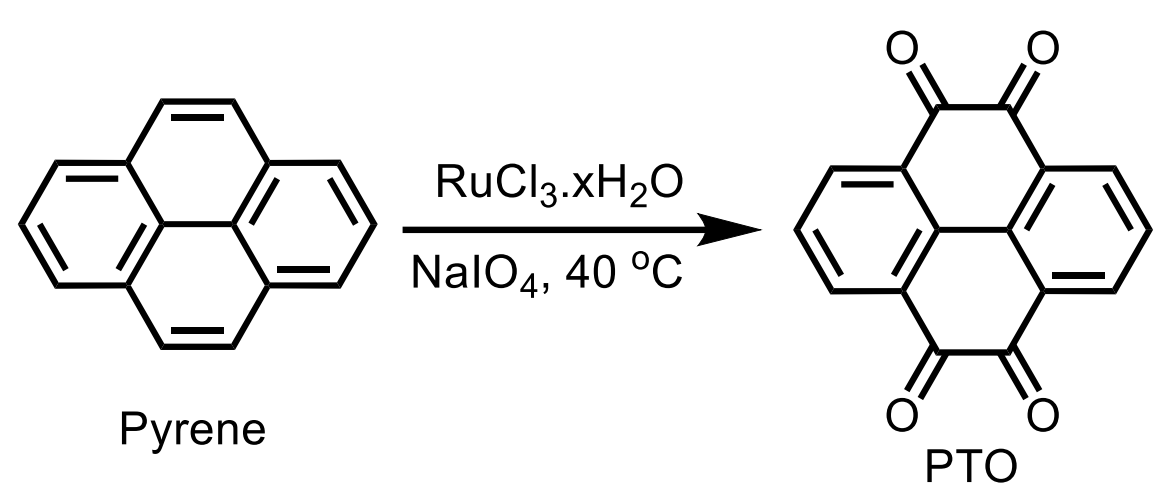

Pyrene-4,5,9,10-tetrone (PTO) was synthesized following reported procedures with minor modifications.[S2,S3] Pyrene (4 g, 20 mmol) was dissolved in a mixture of dichloromethane (80 mL) and acetonitrile (80 mL). Sodium periodate (35.0 g, 164 mmol), deionized water (100 mL), and ruthenium(III) chloride hydrate (0.5 g, 0.42 mmol) were then added, and the reaction mixture was stirred at 40 °C overnight. The resulting suspension was filtered under vacuum, and the solid was washed thoroughly with dichloromethane until the filtrate became light orange. Evaporation of the combined organic washes afforded PTO as an orange solid (1.3 g, 25% yield) without further purification.

^{1}H NMR (400 MHz, DMSO-d_6) δ 8.33 (d, J = 7.7 Hz, 4H), 7.74 (t, J = 7.7 Hz, 2H).

^{13}C NMR (125 MHz, DMSO-d_6) δ 177.5, 134.5, 134.1, 131.9, 130.6.

HRMS (DART-TOF) m/z: calcd for $[C_{16}H_6O_4 + NH_4]^+$: 280.22; found: 280.06.

Synthesis of 2,7-dinitropyrene-4,5,9,10-tetraone (DNPTO)

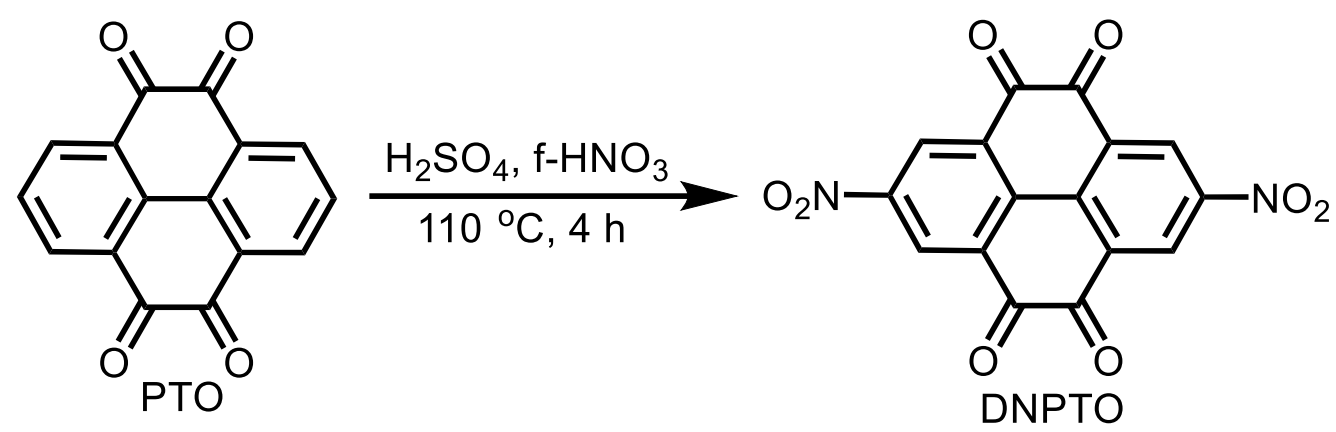

DNPTO was prepared using a modified literature procedure.[S4,S5] PTO (524 mg, 2 mmol) was added to a mixture of concentrated sulfuric acid and fuming nitric acid (20 mL, 4:1 v/v) and heated at 110 °C for 4 h. After cooling to room temperature, the reaction mixture was poured into ice water (200 mL), generating a yellow precipitate. The solid was collected by vacuum filtration, washed thoroughly with water, and dried under vacuum at 70 °C for 24 h to afford DNPTO as a yellow powder (453.9 mg, 64% yield).

^{1}H NMR (400 MHz, DMSO-d_6) δ 8.90 (s, 4H).

^{13}C NMR (125 MHz, DMSO-d_6) δ 174.61, 149.09, 136.23, 134.46, 126.36.

HRMS (DART-TOF) m/z: calcd for $[C_{16}H_4N_2O_8 + Li]^+$: 355.21; found: 355.00.

Synthesis of 2,7-diaminopyrene-4,5,9,10-tetraone (DAPTO)

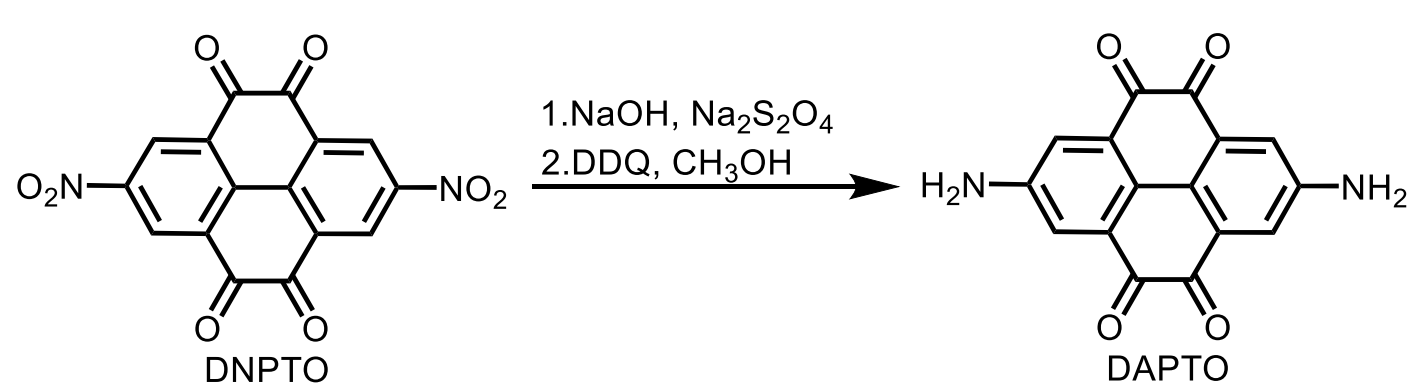

DAPTO was synthesized following a modified literature procedure.[S6,S7] Sodium hydroxide (4.45 g, 112 mmol), sodium hydrosulfite (2.2 g, 12.75 mmol), and DNPTO (500 mg, 1.4 mmol) were added to a two-neck round-bottom flask, which was cycled under vacuum and argon three times. Deionized water (75 mL) was then introduced, and the mixture was stirred at 50 °C for 30 min under argon. The reaction was quenched by pouring the mixture into saturated ammonium chloride solution (250 mL), affording a dark suspension. The solid was collected by vacuum filtration, washed thoroughly with water, and dried under vacuum at room temperature to give the crude reduced intermediate. The crude product (242.5 mg) was subsequently oxidized by stirring with 2,3-dichloro-5,6-dicyano-p-benzoquinone (DDQ, 796 mg, 3.5 mmol) in anhydrous methanol (16

mL) at 35 °C overnight under argon. The reaction mixture was diluted with ethyl acetate (30 mL), and the resulting suspension was isolated by vacuum filtration and washed repeatedly with ethyl acetate. After drying, DAPTO was obtained as a black solid (165 mg, 50% yield).

^{1}H NMR (400 MHz, DMSO-d$_6$) δ 7.36 (s, 4H), 5.95 (s, 4H).

^{13}C NMR (125 MHz, DMSO-d$_6$) δ 178.84, 148.93, 131.23, 124.83, 119.29.

HRMS (DART-TOF) m/z: calcd for $[C_{16}H_{10}N_2O_4]^+$: 294.27; found: 293.10.

Synthesis of PhA-PTO

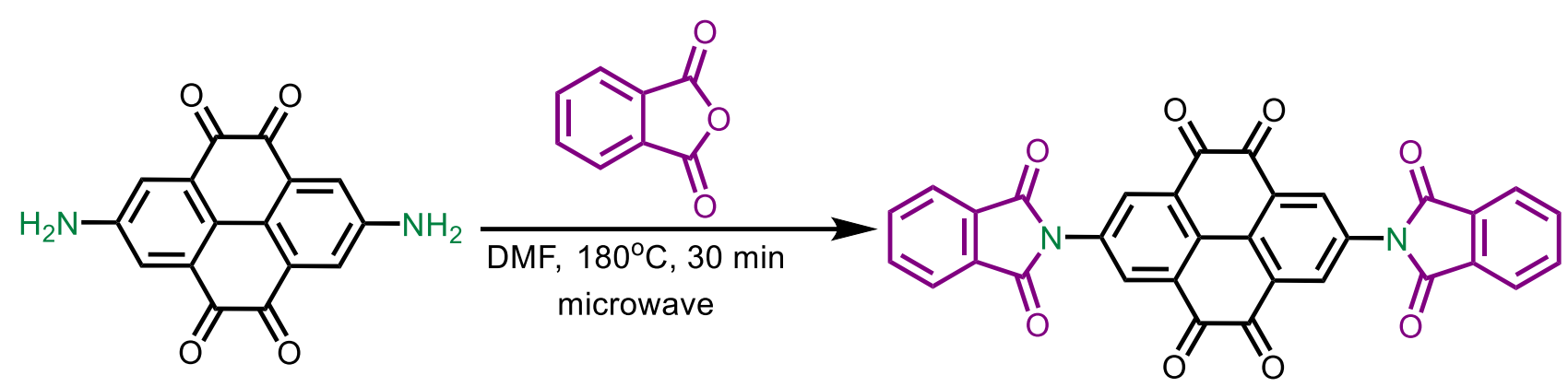

PhA–PTO was synthesized by combining phthalic anhydride (52 mg, 0.345 mmol), DAPTO (50 mg, 0.172 mmol), and anhydrous DMF (5 mL) in a microwave vial, followed by sonication for 5 min to ensure complete dispersion. The vial was then sealed and heated under microwave irradiation at 180 °C for 30 min. After cooling to room temperature, the reaction mixture was poured into methanol (30 mL) to precipitate the product. The resulting suspension was collected by vacuum filtration and washed sequentially with methanol, water, and acetone. The solid was dried under vacuum at 70 °C overnight to afford PhA–PTO as a black powder (38.2mg, 40% yield).

Synthesis of PMDA-PTO

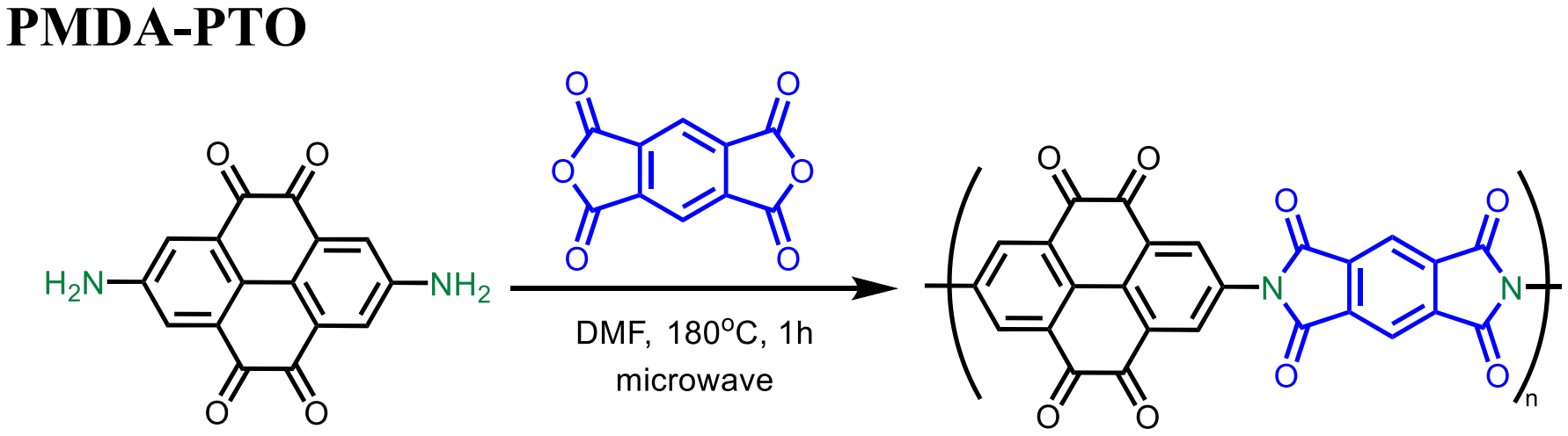

PMDA–PTO was synthesized following the same procedure as PhA–PTO, with adjusted stoichiometry. DAPTO (150 mg, 0.517 mmol), pyromellitic dianhydride (PMDA, 112.7 mg, 0.517 mmol), and anhydrous DMF (8 mL) were combined in a microwave vial and sonicated for 5 min to afford a homogeneous suspension. The vial was then sealed and heated under microwave irradiation at 180 °C for 1 h. After cooling to room temperature, the reaction mixture was poured into methanol to precipitate the product. The resulting suspension was collected by vacuum

filtration, washed sequentially with methanol, DMF, water, and acetone, and dried under vacuum at 70 °C overnight to afford PMDA–PTO as a black solid (196 mg, 80% yield).

Synthesis of PMDA-PTO-CNT

The PMDA–PTO–CNT composite was synthesized following the same procedure used for PMDA–PTO, with the addition of carbon nanotubes (CNT). Based on the consistent isolated yield of PMDA–PTO (196 mg), a 50 wt% CNT loading was targeted. Accordingly, CNT (196 mg) were added directly to the reaction mixture containing DAPTO, PMDA, and anhydrous DMF prior to microwave irradiation. The mixture was sonicated for 5 min to ensure uniform dispersion of CNT, then sealed and heated at 180 °C for 1 h under microwave conditions. After cooling to room temperature, the reaction mixture was poured into methanol to precipitate the composite. The solid was collected by vacuum filtration, washed sequentially with methanol, DMF, water, and acetone, and dried under vacuum at 70 °C overnight to afford PMDA–PTO–CNT as a black composite powder.

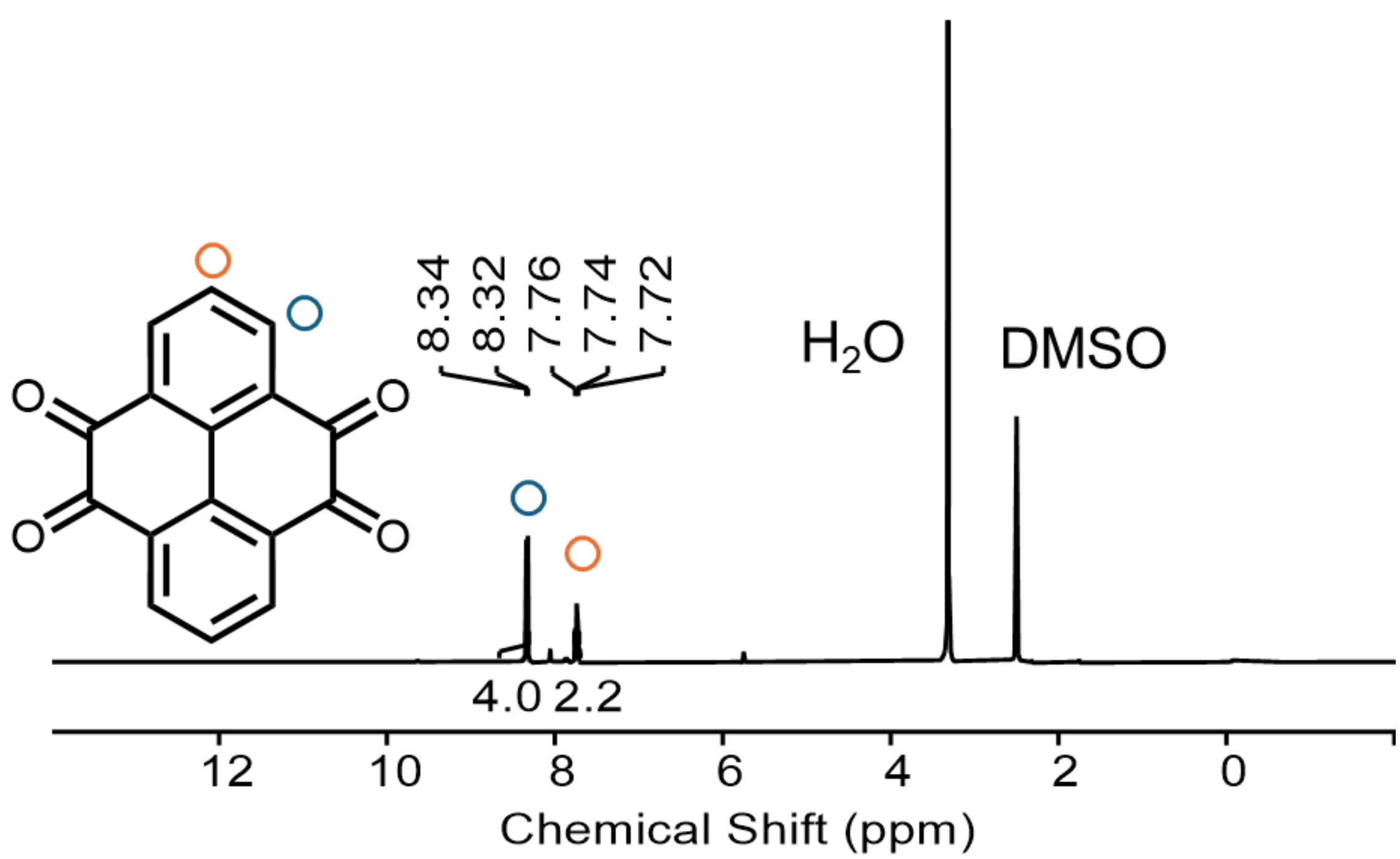

Figure S1. ^{1}H NMR (400 MHz, DMSO-d6) spectrum of PTO.

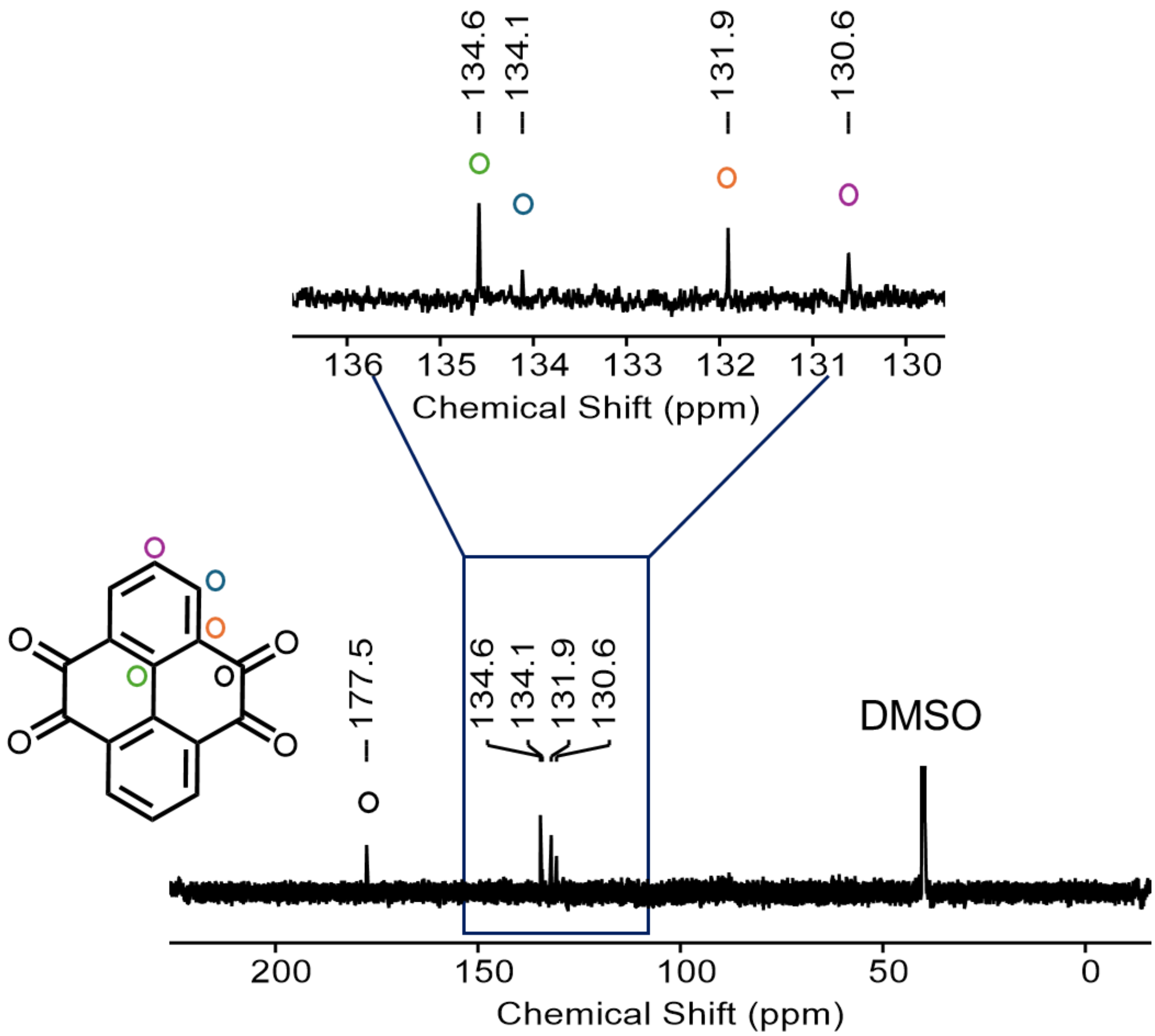

Figure S2. ^{13}C NMR (125 MHz, DMSO-d6) spectrum of PTO.

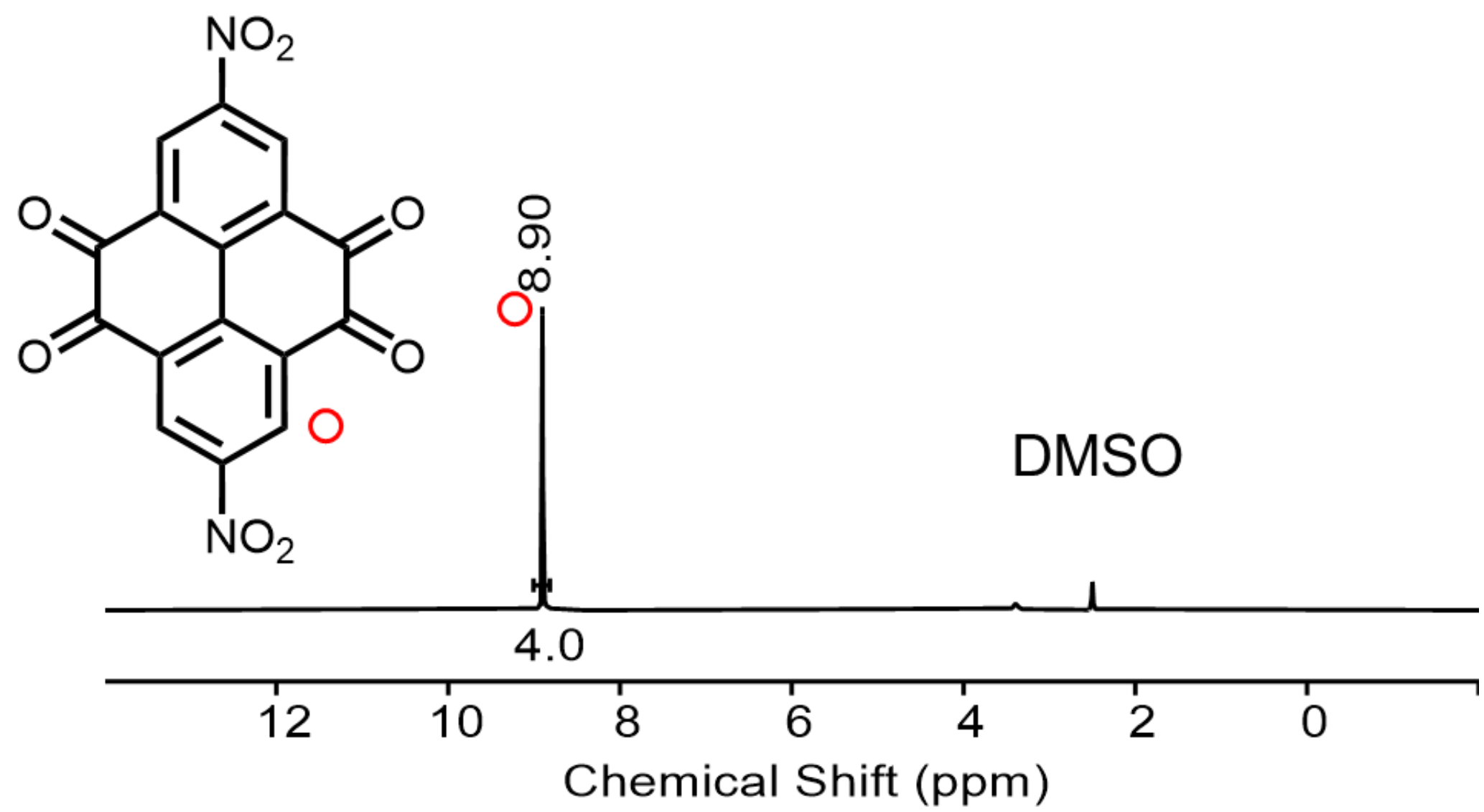

Figure S3. ^{1}H NMR (400 MHz, DMSO-d6) spectrum of DNPTO.

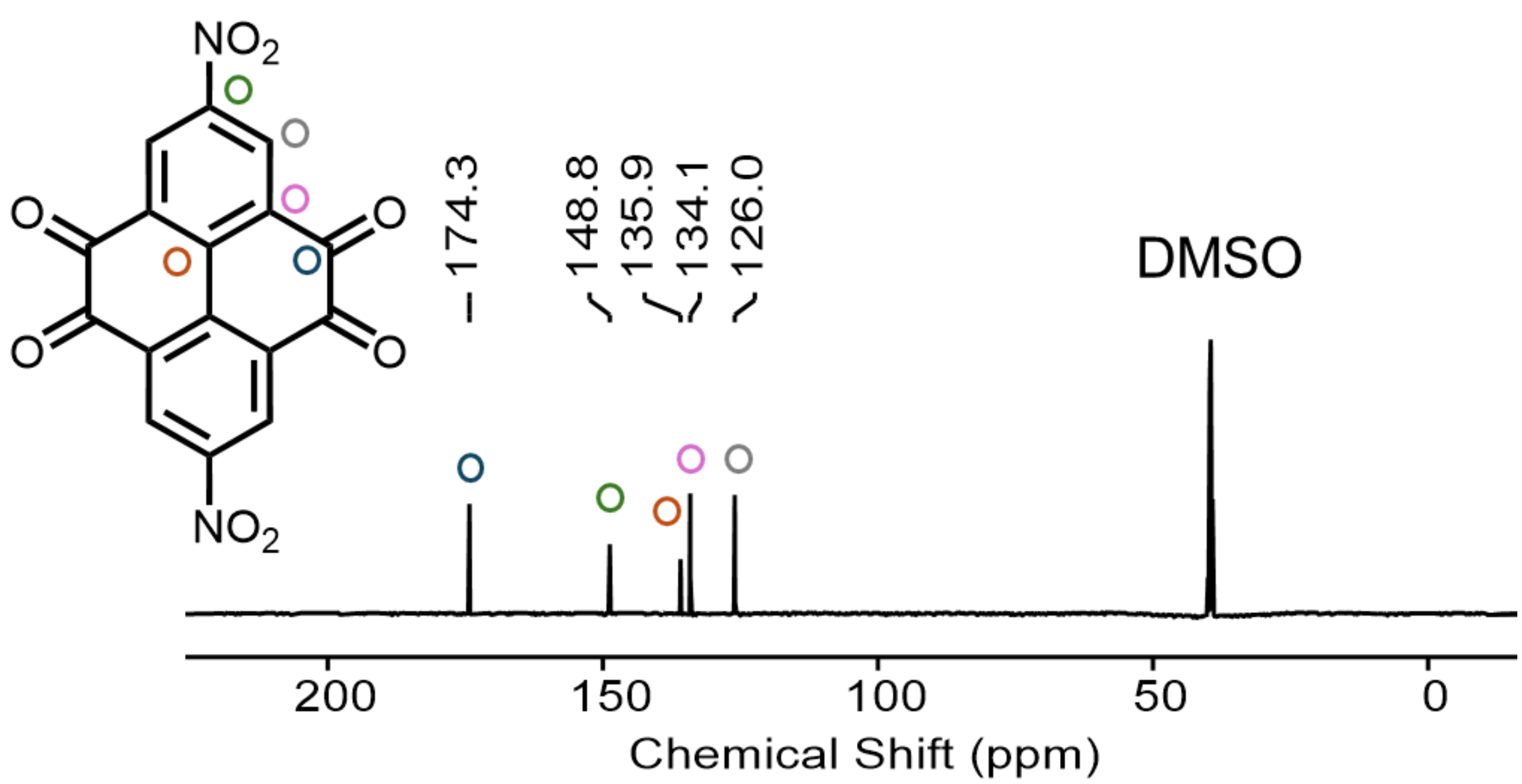

Figure S4. ^{13}C NMR (125 MHz, DMSO-d6) spectrum of DNPTO.

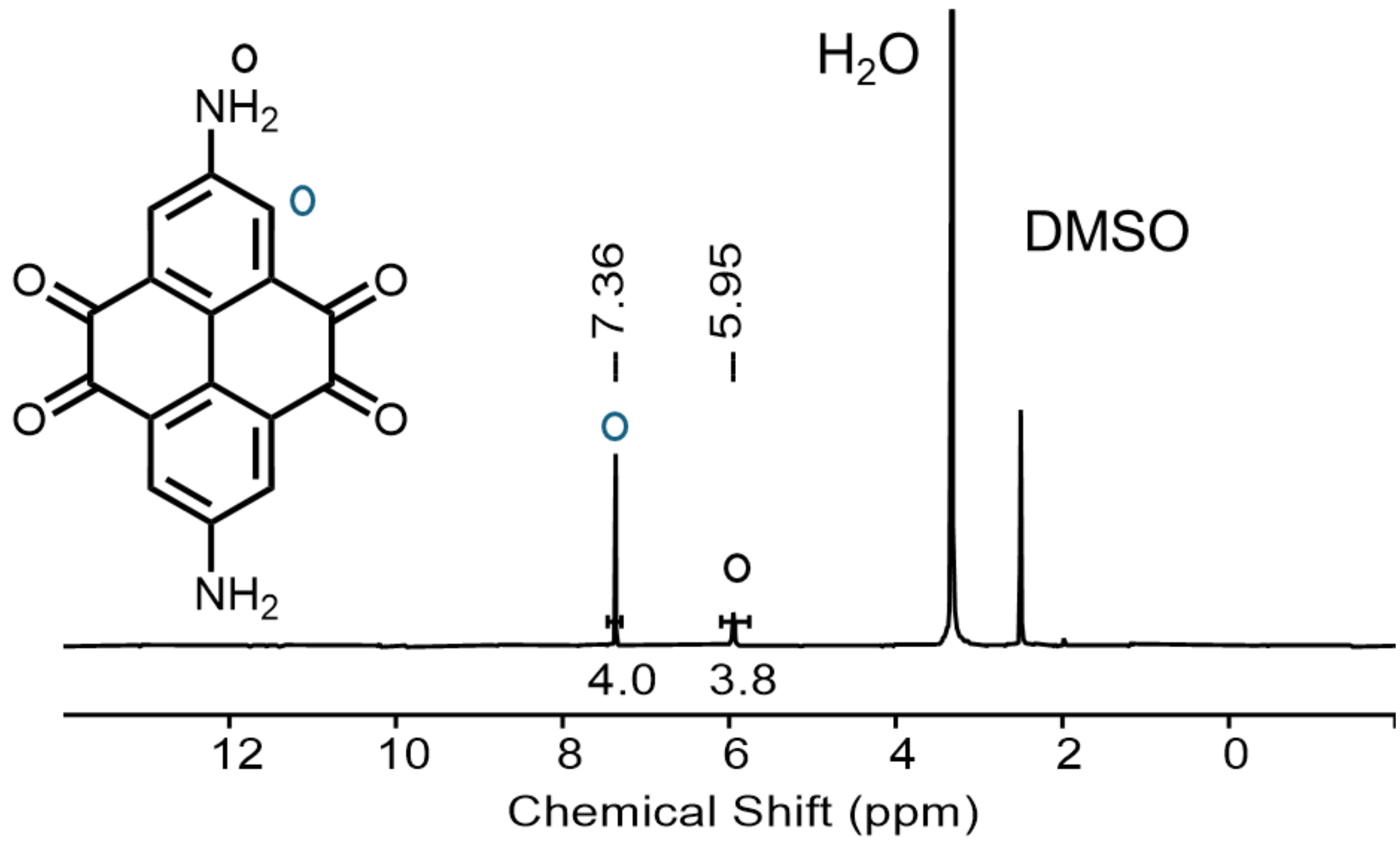

Figure S5. ^{1}H NMR (400 MHz, DMSO-d6) spectrum of DAPTO.

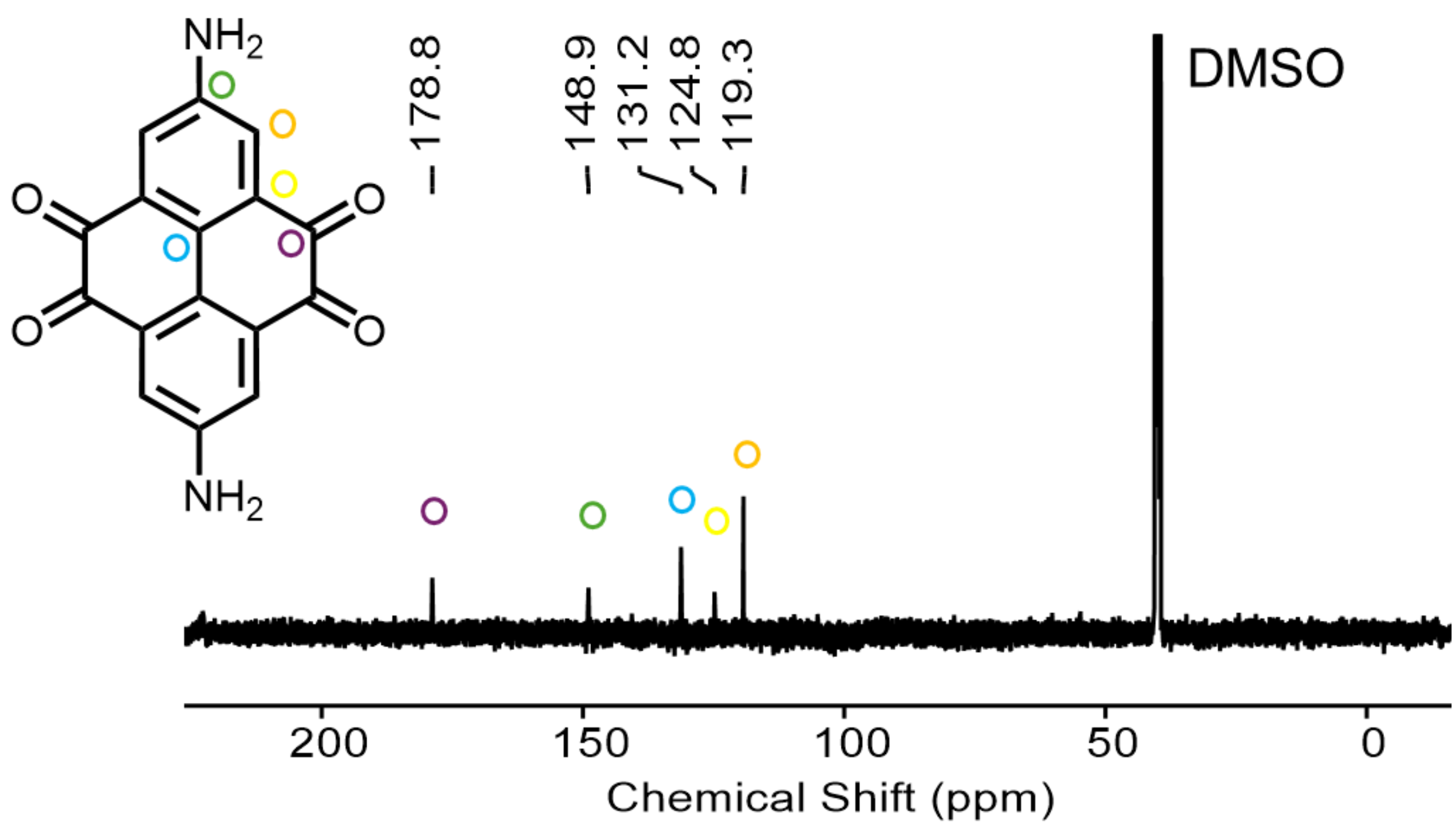

Figure S6. ^{13}C NMR (125 MHz, DMSO-d6) spectrum of DAPTO.

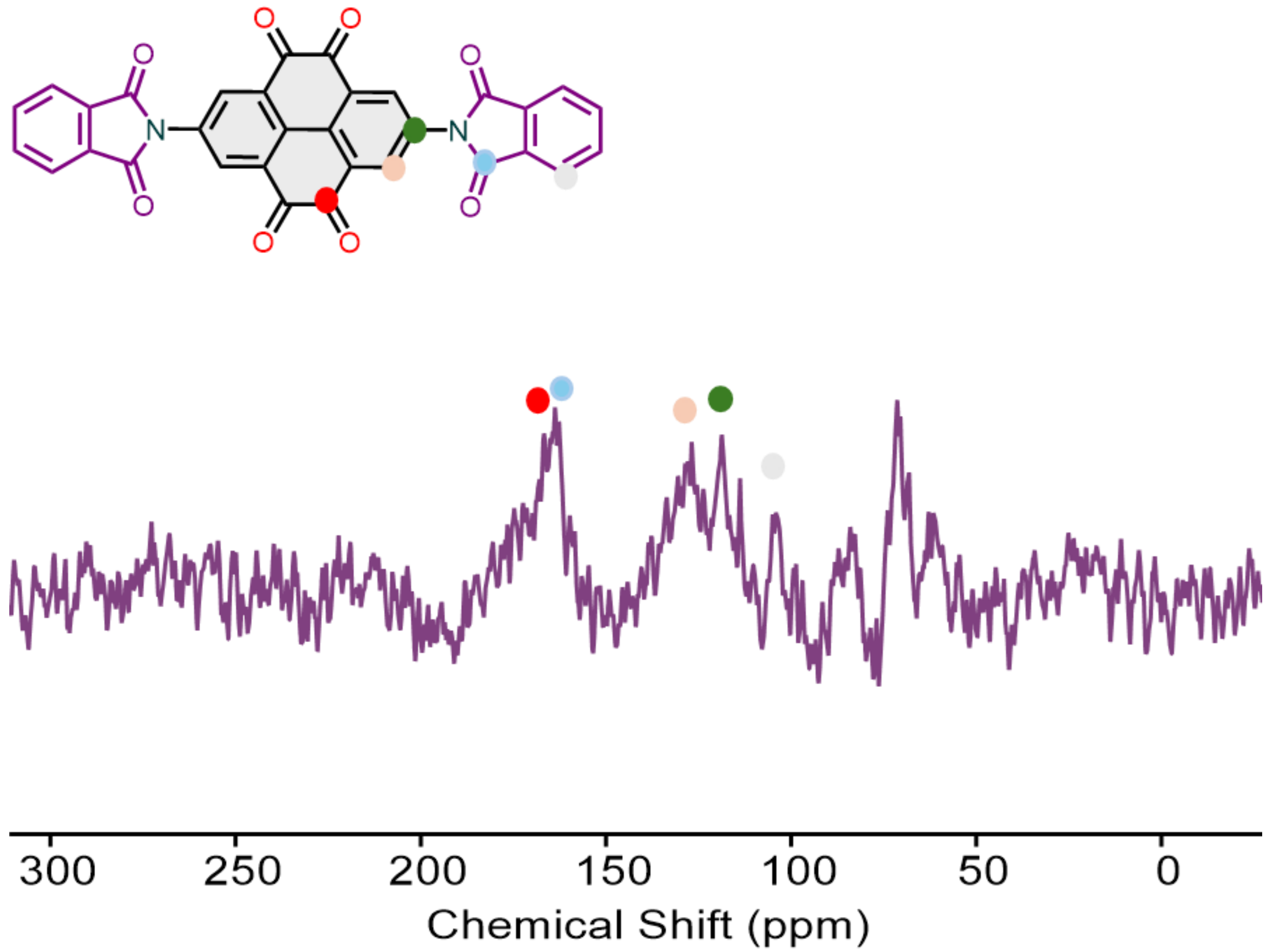

Figure S7. Solid-state ^{13}C CP-MAS NMR spectrum of PhA-PTO. Weak and broad intensity observed in the ~60–80 ppm region does not correspond to genuine chemical resonances, as the structure contains no aliphatic or heteroatom-bound sp^3 carbons. These features are attributed to spinning sidebands arising from aromatic carbons exhibiting large chemical shift anisotropy (CSA). Such CSA-induced sidebands are commonly observed in rigid, π-conjugated aromatic solids under MAS conditions and reflect the highly anisotropic local environments of the aromatic core.[S8–S10]

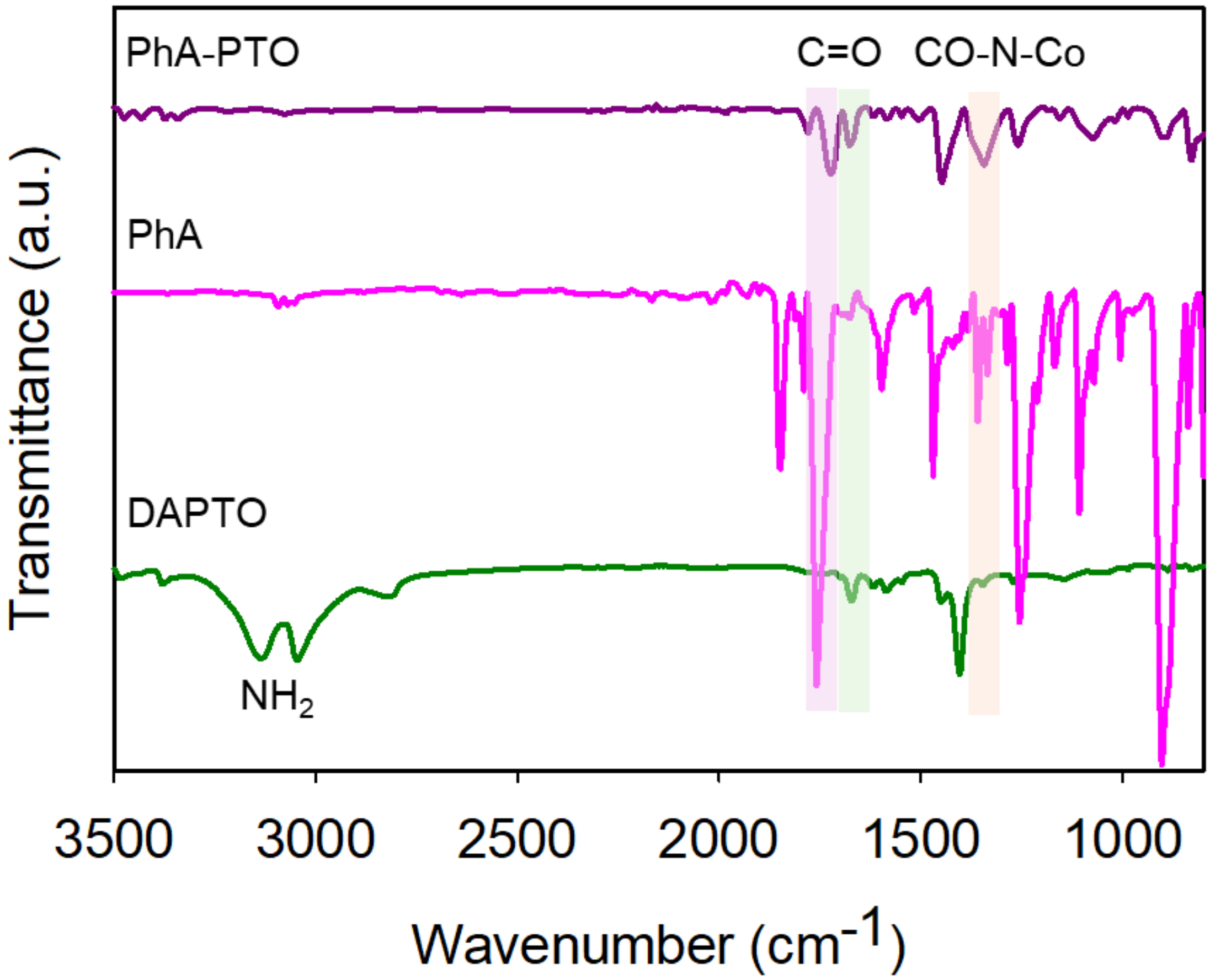

Figure S8. FTIR spectra of DAPTO, PhA, and PhA-PTO.

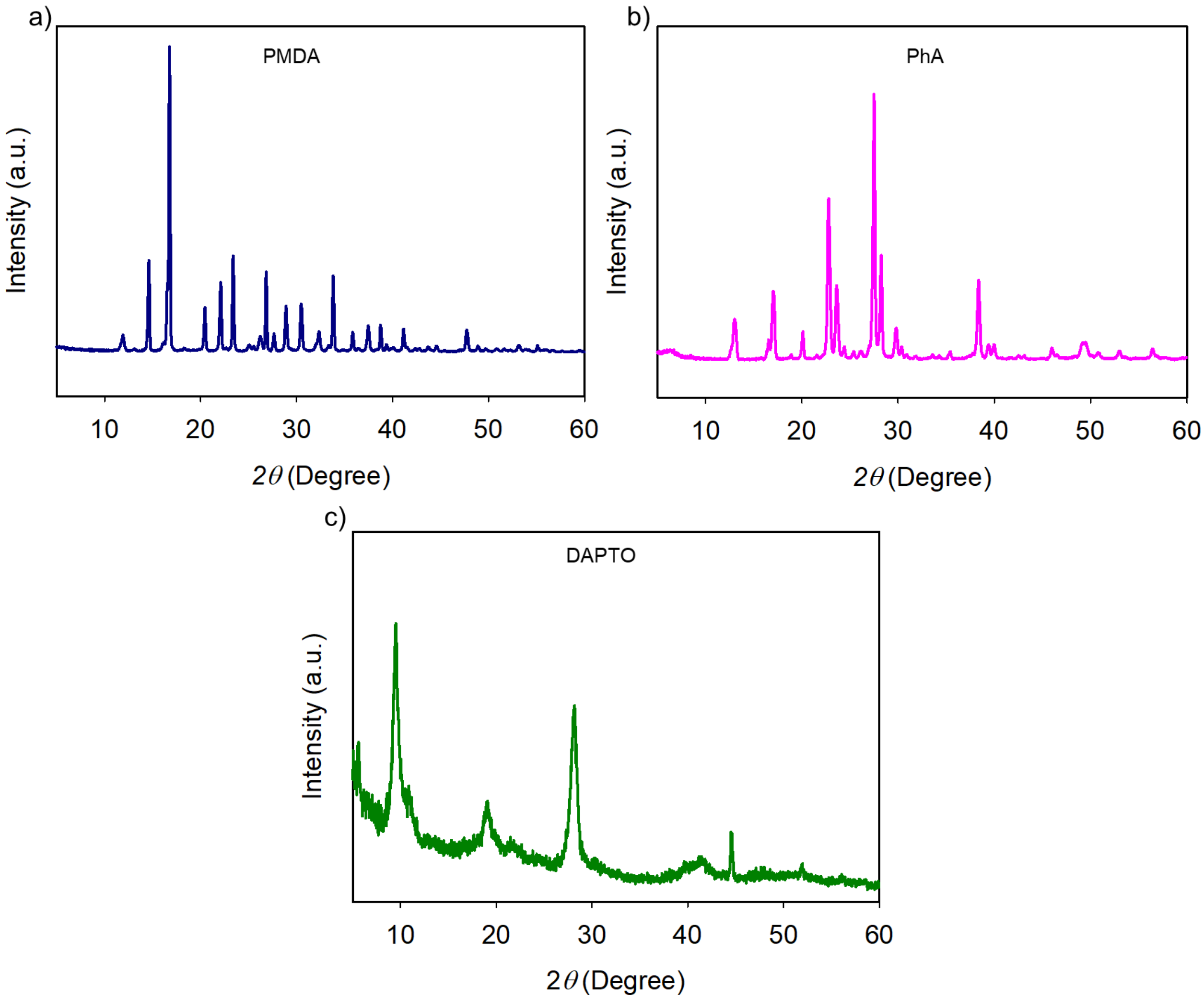

Figure S9. PXRD patterns of PMDA, PhA, and DAPTO.

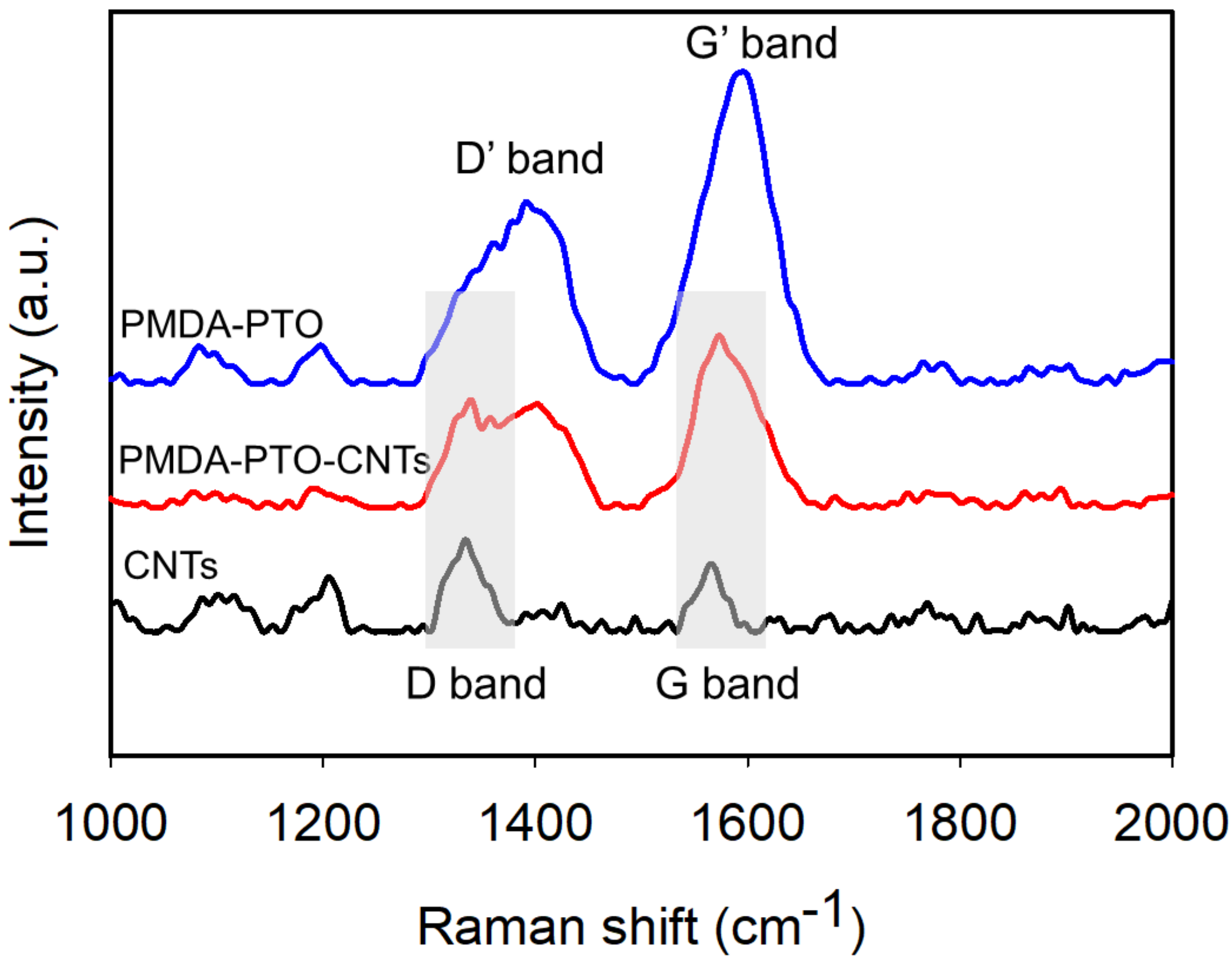

Figure S10. Raman spectra of PMDA–PTO, PMDA–PTO–CNT, and CNT.

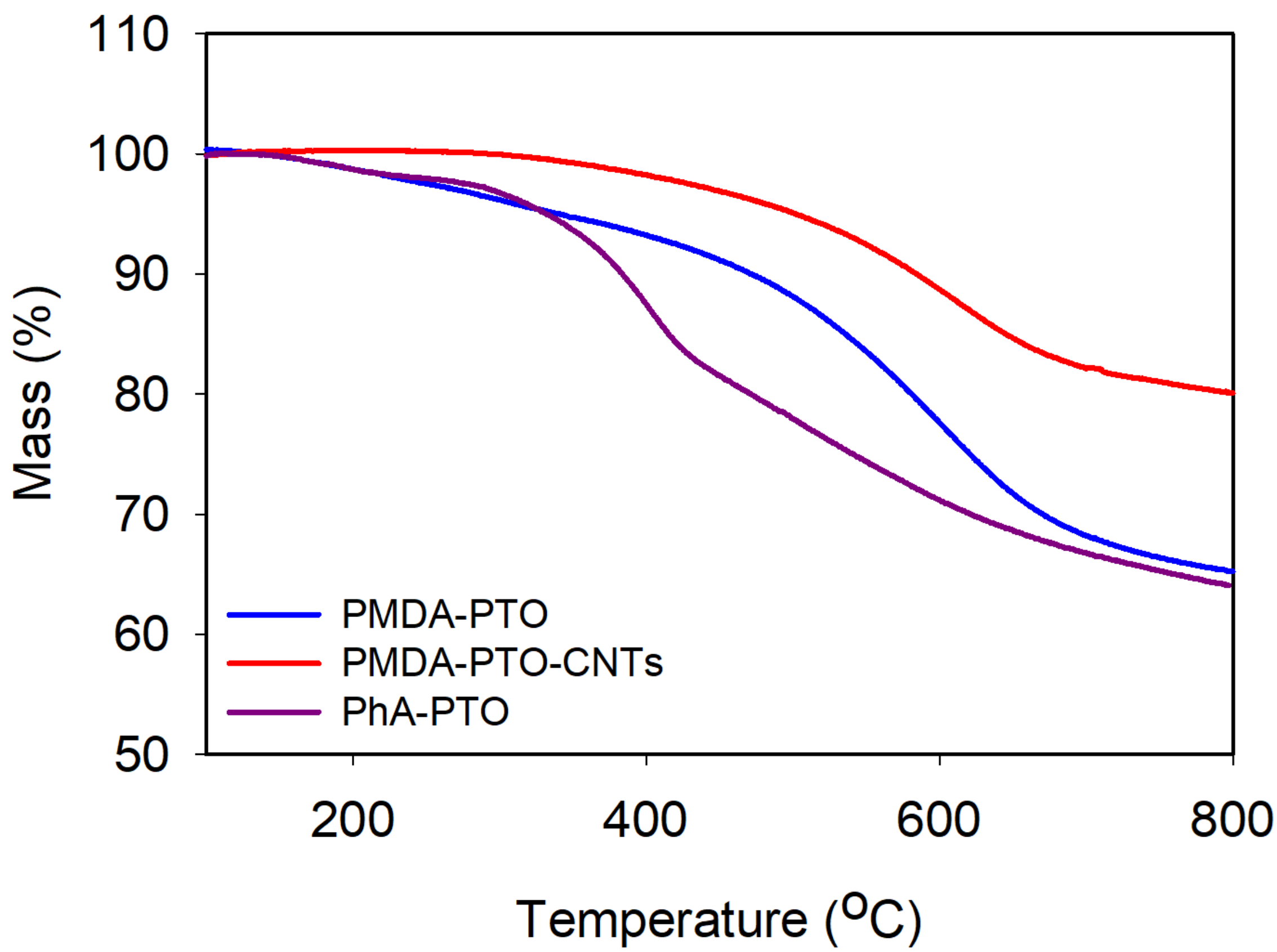

Figure S11. Thermogravimetric analysis (TGA) for PhA-PTO, PMDA-PTO, and PMDA–PTO–CNT.

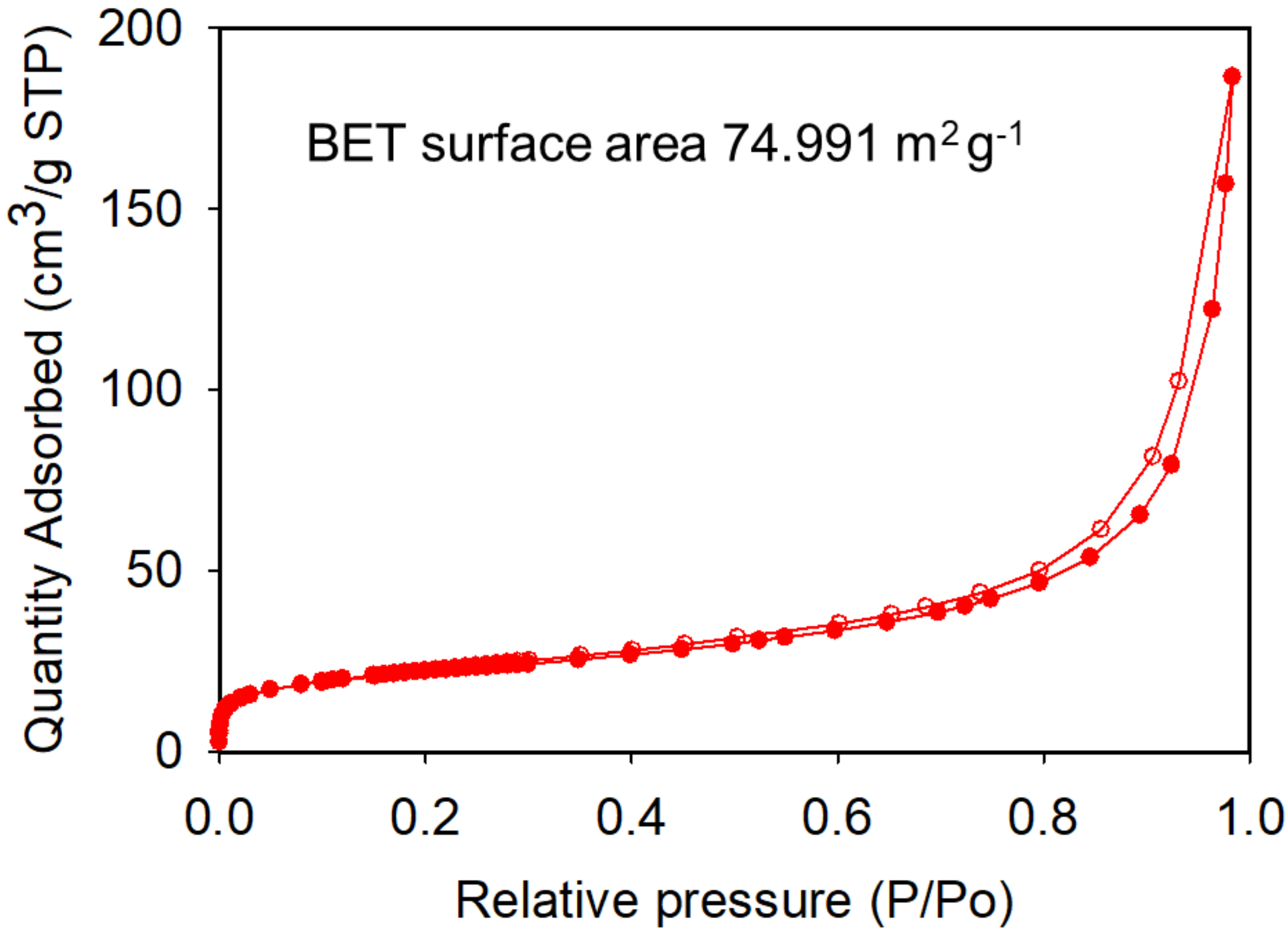

Figure S12. N_2 sorption isotherm of PMDA-PTO-CNT.

3. Calculation of theoretical capacity of PMDA-PTO and PhA-PTO

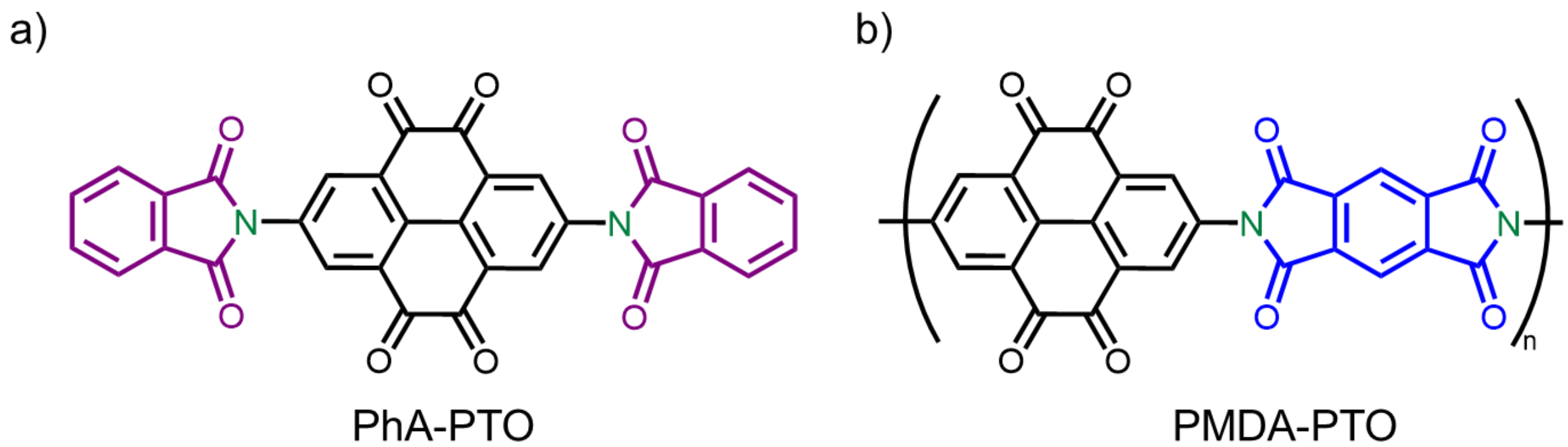

Scheme 1: Chemical structure of (a) PhA-PTO, and (b) PMDA-PTO.

The theoretical gravimetric capacity (C_t) of the organic cathode materials was calculated according to the following equation:

$$C_t = \frac{nF}{M_w} \quad S1$$

where n is the number of electrons transferred per redox unit, F is the Faraday constant (96485 C mol^{-1}), and M_w is the molecular weight of the electrochemically active repeat unit.

As shown in Scheme S1, PhA–PTO consists of one PTO unit and two phthalic anhydride–derived imide units, giving a molecular weight of 552.45 g mol^{-1}. In contrast, PMDA–PTO is composed of a PTO repeat unit and a PMDA-derived imide unit, resulting in a lower repeat-unit molecular weight of 510.37 g mol^{-1}. The number of electrons involved in the redox process (n) was estimated based on the carbonyl redox activity: four electrons from the PTO unit, together with two electrons from the two phthalic units in PhA–PTO or two electrons from the PMDA unit in PMDA–PTO. Accordingly, the theoretical capacities were calculated to be 291 mAh g^{-1} for PhA–PTO and 315 mAh g^{-1} for PMDA–PTO.

4. Electrochemical measurements

All electrochemical measurements were carried out using CR2032-type coin cells assembled under ambient conditions. Zinc metal foil was used as both the counter and reference electrode, while PhA–PTO, PMDA–PTO, or PMDA–PTO–CNT served as the cathode active materials.

Cathode preparation. Poly(vinylidene fluoride) (PVDF) was first dissolved in isopropanol in a small vial under magnetic stirring. After complete dissolution, the active material (PhA–PTO, PMDA–PTO, or PMDA–PTO–CNT) was added and stirred for 15 min, followed by the addition of Super P conductive carbon and further stirring for 15 min. The resulting slurry (70 mg mL^{-1}) was subjected to five cycles of sonication (1 h) and brief stirring (1 min) to ensure homogeneous dispersion. The mass ratio of active material: Super P: PVDF was maintained at 40:50:10. The homogeneous slurry was then cast onto stainless-steel foil current collectors (4000 × 100 × 0.01 mm) using a doctor blade with a wet thickness of 200 μm and dried on a hot plate at 80 °C overnight.

Cell assembly. The dried electrode films were punched into 15 mm diameter discs. Zinc foil (0.1 mm thickness, 100 mm diameter) was used as the anode, and a thick (Whatman GF/B, 19 mm diameter) served as the separator. The coin cells (CR2032, MTI Corporation) were assembled with 80 μL of 2 M $ZnSO_4 \cdot 7H_2O$ aqueous electrolyte. A stainless-steel spacer (0.5 mm thickness) and a spring were included to ensure uniform pressure, and the cells were sealed using a digital pressure-controlled electric crimper (MTI Corporation). The active material loading was controlled within the range of 0.4–0.6 mg cm^{-2}. To ensure sufficient electrolyte infiltration and electrode wetting, all assembled cells were rested for 12–18 h prior to electrochemical testing.

Electrochemical testing. Galvanostatic charge–discharge (GCD) tests and rate capability measurements were performed using a Neware BTS-4000 battery testing system at 25 °C. Specific capacities and current densities were calculated based on the mass of the active material in the cathode. Cyclic voltammetry (CV) measurements were conducted using a Bio-Logic BCS-805 battery tester equipped with a frequency response analyzer. Electrochemical impedance spectroscopy (EIS) measurements were carried out over a frequency range from 10 kHz to 100 mHz at open-circuit voltage, using a sinusoidal voltage perturbation of 10 mV. Prior to EIS measurements, the cells were cycled for ten cycles at a current density of 50 mA g^{-1} and subsequently rested for 10 h to reach equilibrium.

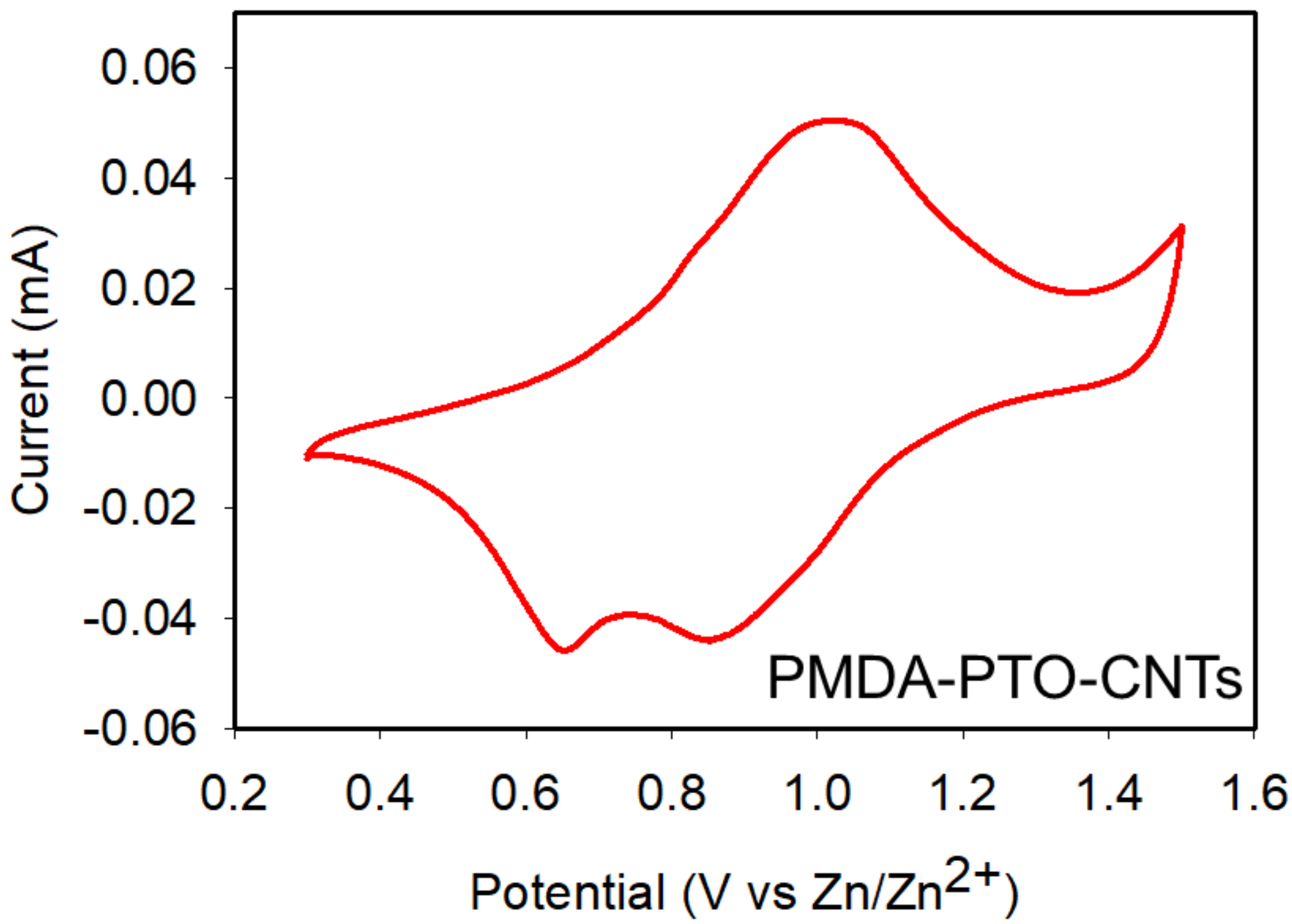

Figure S13. CV curve of PMDA–PTO–CNT at 0.1 mV s^{-1}.

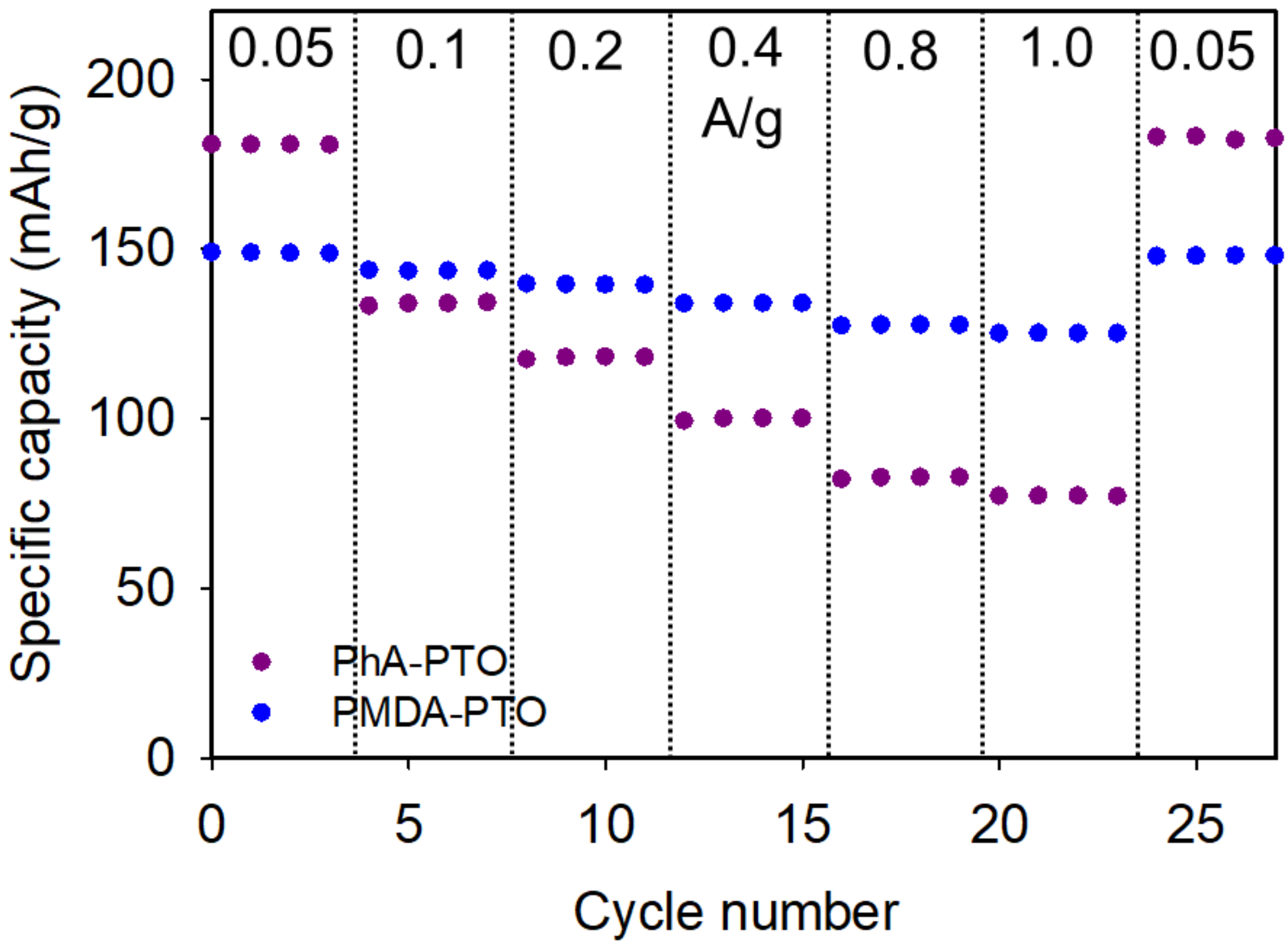

Figure S14. Rate capability of PhA-PTO (purble), and PMDA-PTO (blue) at different current density.

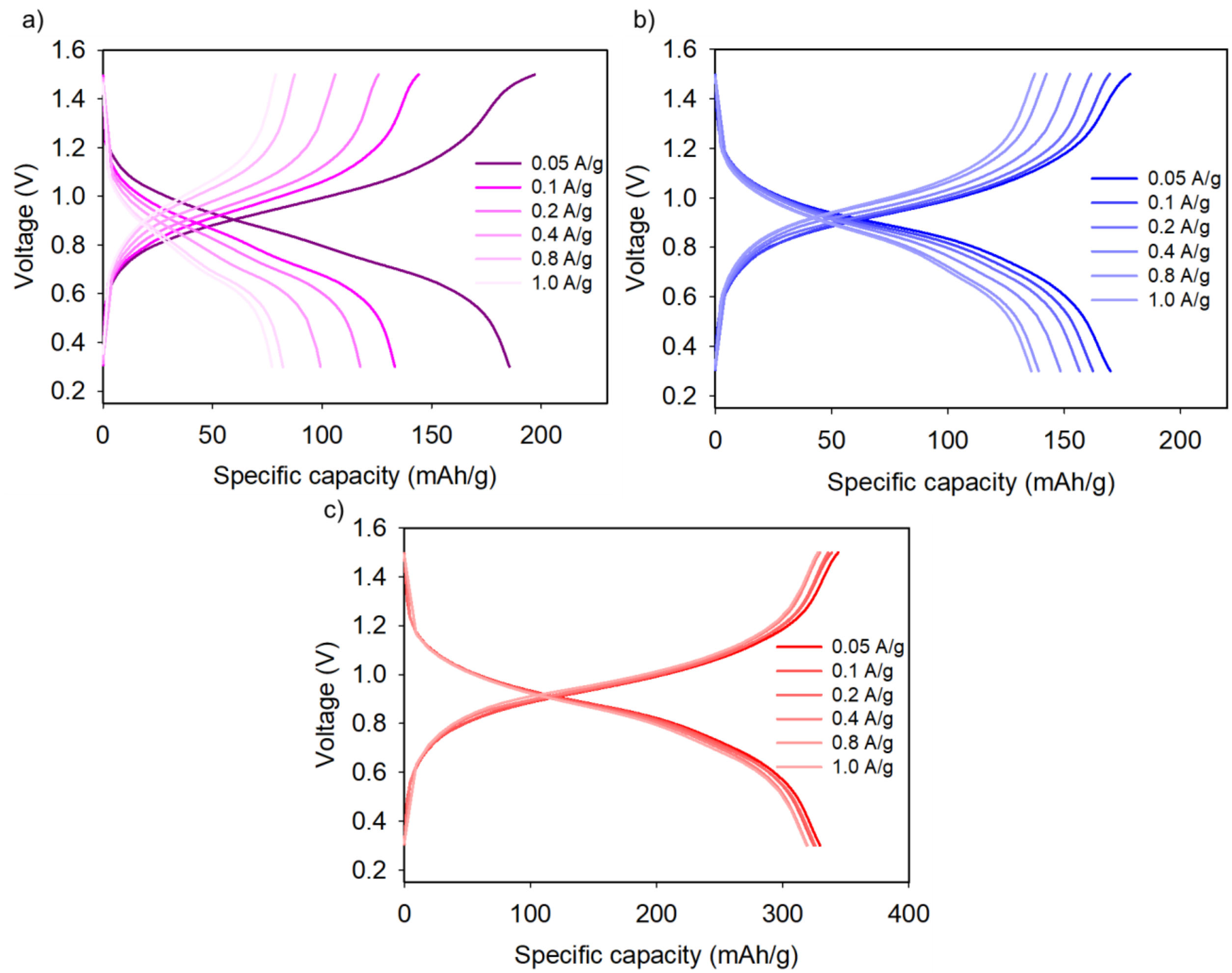

Figure S15: The Galvanostatic charge discharge curves of (a) PhA-PTO, (b) PMDA-PTO, and (c) PMDA–PTO–CNT.

Calculation of utilization efficiency for PMDA–PTO–CNT

The utilization efficiency of PMDA–PTO in the PMDA–PTO–CNT composite was calculated as follows:[S6,S11]

$$Utilization\ Efficiency\ = \frac{Q_{PMDA-PTO\ real}}{Q_{PMDA-PTO\ theoritical}} \qquad S2$$

Where Q_{real} is the experimental discharge capacity after subtracting the capacity contribution from CNT:

$$Q_{PMDA-PTO,real} = Q_{exp} - \left(\frac{w_{CNT}}{w_{PMDA-PTO}} Q_{CNT}\right) \qquad S3$$

Where Q_{exp} is the experimental capacity of PMDA–PTO–CNT obtained from the rate capability at 0.05 A g^{-1}. W_{CNT}, $W_{PMDA\text{-}PTO}$ are the weight % of CNT and PMDA–PTO in the electrode composition respectively. Q_{CNT} is the capacity of bare CNT.

The measured capacity at 0.05 A g^{-1} of PMDA–PTO–CNT was 328 mAh g^{-1}. The measured CNT capacitance under the same conditions was 20 mAh g^{-1}, resulting in an active material capacity of 308 mAh g^{-1}. The theoretical capacity of PMDA-PTO is 315 mAh g^{-1}, which results in a capacity utilization of 97.7%.

The capacity contribution of CNT was estimated from a separately measured CNT electrode under identical electrochemical conditions, following previously reported approaches for polymer–carbon composite electrodes.[S6,S11] While interactions between PMDA–PTO and CNTs may influence the exact electrochemical contribution of CNTs within the composite, the independently measured CNT capacity provides a practical experimental estimate for evaluating the utilization efficiency of the polymer component.

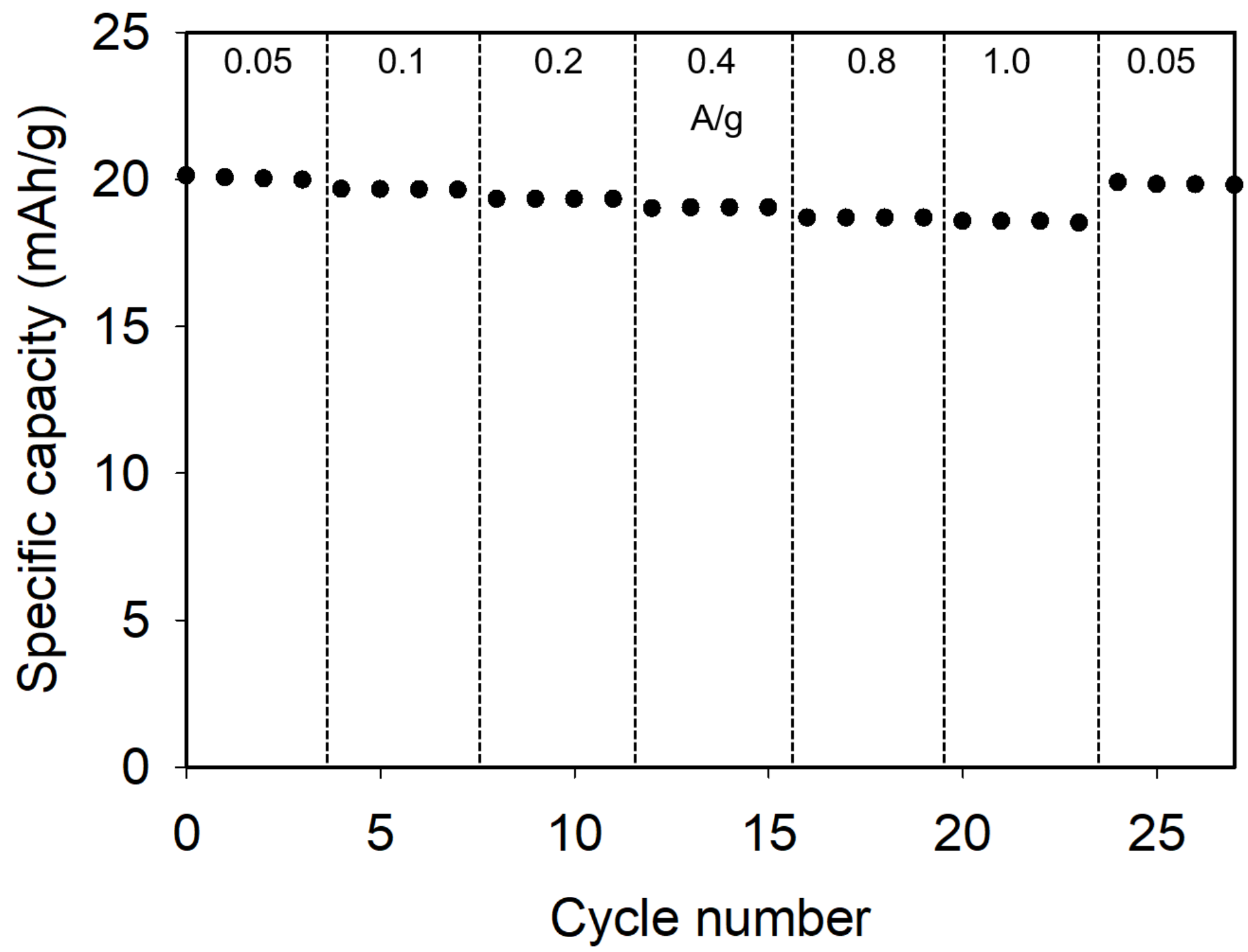

Figure S16. Rate capability of the carbon-control electrode composed of PVDF/CNT/Super P in a mass ratio of 10:40:50.

Table S1. Rate capability data of PhA–PTO, PMDA–PTO, and PMDA–PTO–CNT electrodes measured at different current densities.

Current density (A g^{-1})	PhA-PTO	PMDA-PTO	PMDA-PTO-CNT
**Specific capacity mAh g^{-1}**			
**50**	181.3	150.6	328.2
**100**	135.5	144.7	325.8
**200**	119.2	141.4	324.6
**400**	101.5	135.5	322.3
**800**	83.8	128.9	320.8
**1000**	78.5	127.0	319.9
**2000**	------	119.6	318.5
**5000**	------	107.4	317.2
**10000**	------	101.3	316.8
**20000**	------	92.7	316.0

Table S2. Comparison of the electrochemical performance of PMDA–PTO–CNT with representative organic cathode materials for aqueous zinc-ion batteries.

Active electrode material	Theoretical capacity (mAh g^{-1})	Voltage range (V vs Zn/Zn^{2+})	Low-rate capacity (mAh g^{-1})	High-rate capacity (mAh g^{-1})	Long cycling performance	Ref.
PMDA-PTO-CNT (60%)	315	0.3-1.5	273 at 0.05 A g^{-1}	245 at 20 A g^{-1}	235 after 1600 cycles at 5 A g^{-1}	This work
PMDA-PTO-CNT (40%)	315	0.3-1.5	328 at 0.05 A g^{-1}	316 at 20 A g^{-1}	300 after 10000 cycles at 5 A g^{-1}	This work

NQ@CNT	339	0.1-1.7	333.5 at 1C	------	137.2 after 1500 cycles at 1C	[S12]
TABQ	302	0.4-1.3	303 at 0.1 A g^{-1}	213 at 5 A g^{-1}	200 after 1000 cycles at 5 A g^{-1}	[S13]
PTO	387	0.3-1.5	336 at 0.04 A g^{-1}	113 at 20 A g^{-1}	145 after 1000 cycles at 3 A g^{-1}	[S14]
π-PMC	136.3	0-1.0	122.9 at 0.2 A g^{-1}	76.9 at 32 A g^{-1}	83.8 after 1000 cycles at 8 A g^{-1}	[S15]
$PoPD_H$	301	0.3-1.3	318 at 0.05 A g^{-1}	95 at 5 A g^{-1}	148.5 after 3000 cycles at 1 A g^{-1}	[S16]
DNPTO	304	0.3-1.5	300 at 0.05 A g^{-1}	------	270 after 200 cycles at 0.05 A g^{-1}	[S5]
PPPA	-----	0.2-1.8	210.2 at 0.05 A g^{-1}	139 at 5 A g^{-1}	92 after 20000 cycles at 5 A g^{-1}	[S17]
PDpBQ	187.5	0.9-1.6	120 at 0.1 A g^{-1}	63 at 5 A g^{-1}	81 after 200 cycles at 2 A g^{-1}	[S18]
PQAH	213.9	0-1.5	203.4 at 0.1 A g^{-1}	106 at 5 A g^{-1}	83.4 after 10000 cycles at 5 A g^{-1}	[S19]
rPOP	-----	0.1-1.6		-----	40 after 30000	[S20]

			120 at 0.1 A g^{-1}		cycles at 2 A g^{-1}	

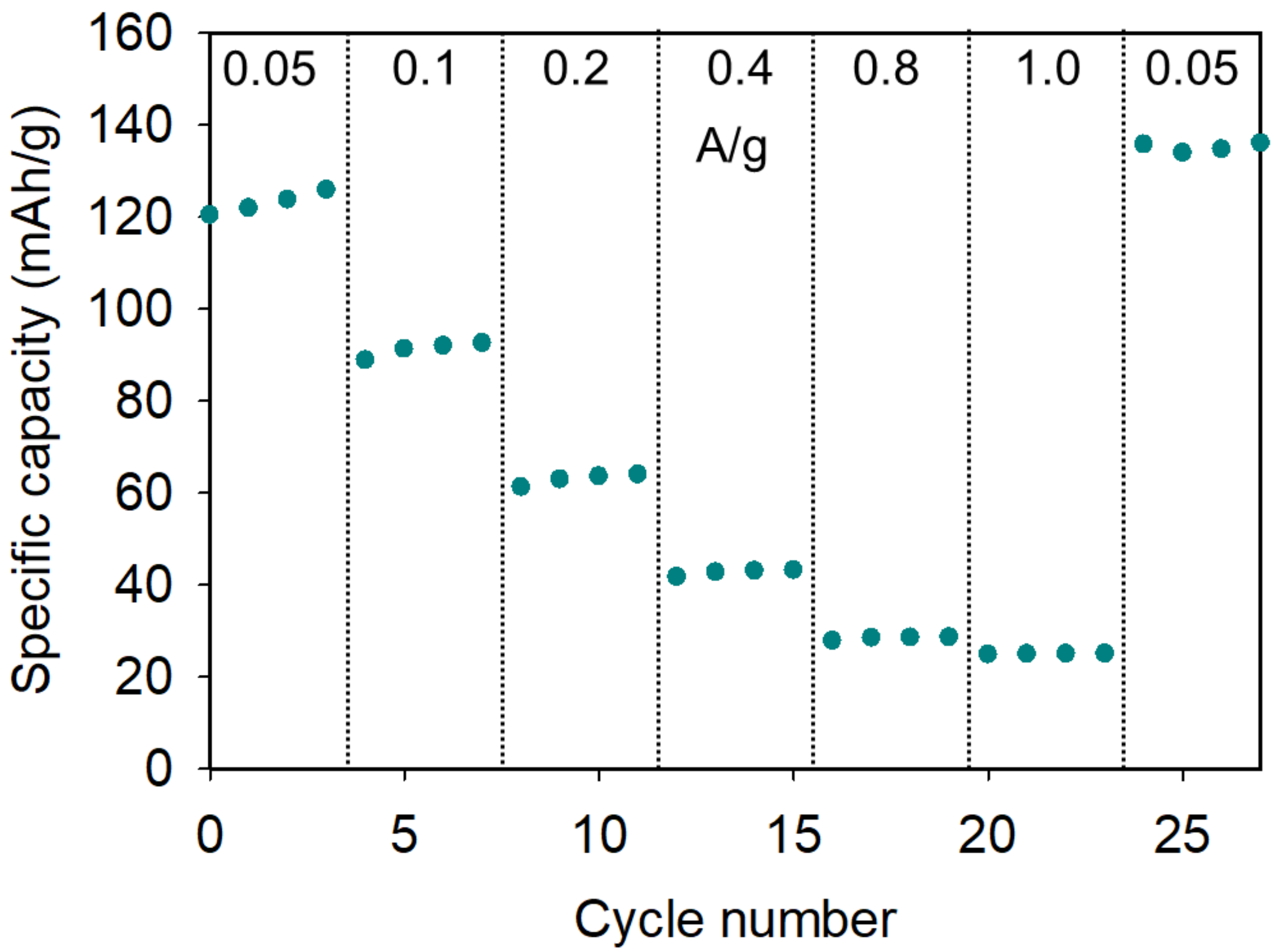

Figure S17. Rate capability of the PMDA–PTO–CNT electrode fabricated without Super P, using a PVDF/PMDA–PTO–CNT mass ratio of 10:90.

5. Post-cycling characterization

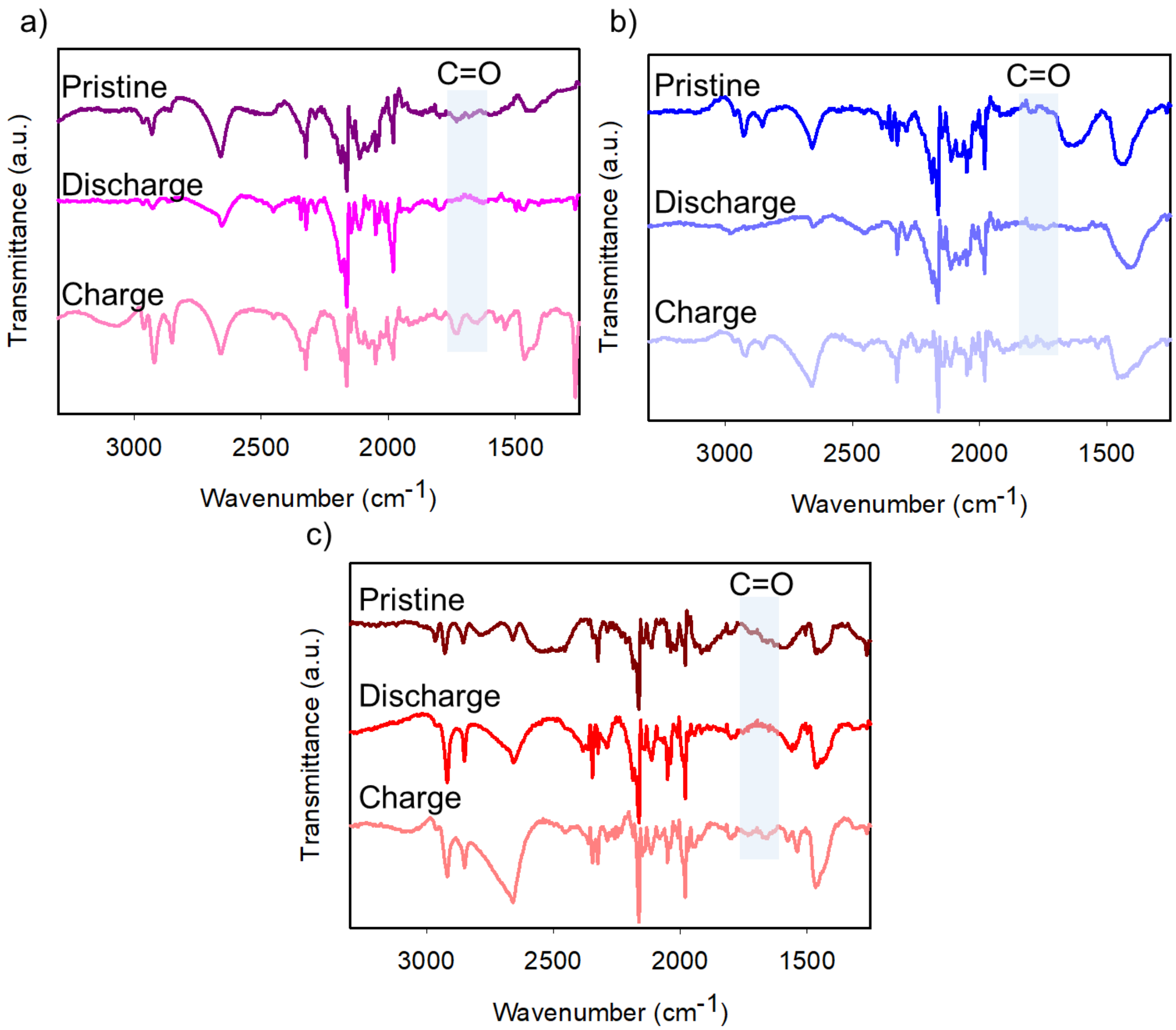

Figure S18. Ex-situ FT-IR spectra of (a) PhA-PTO, (b) PMDA-PTO, and (c) PMDA–PTO–CNT at different electrochemical states.

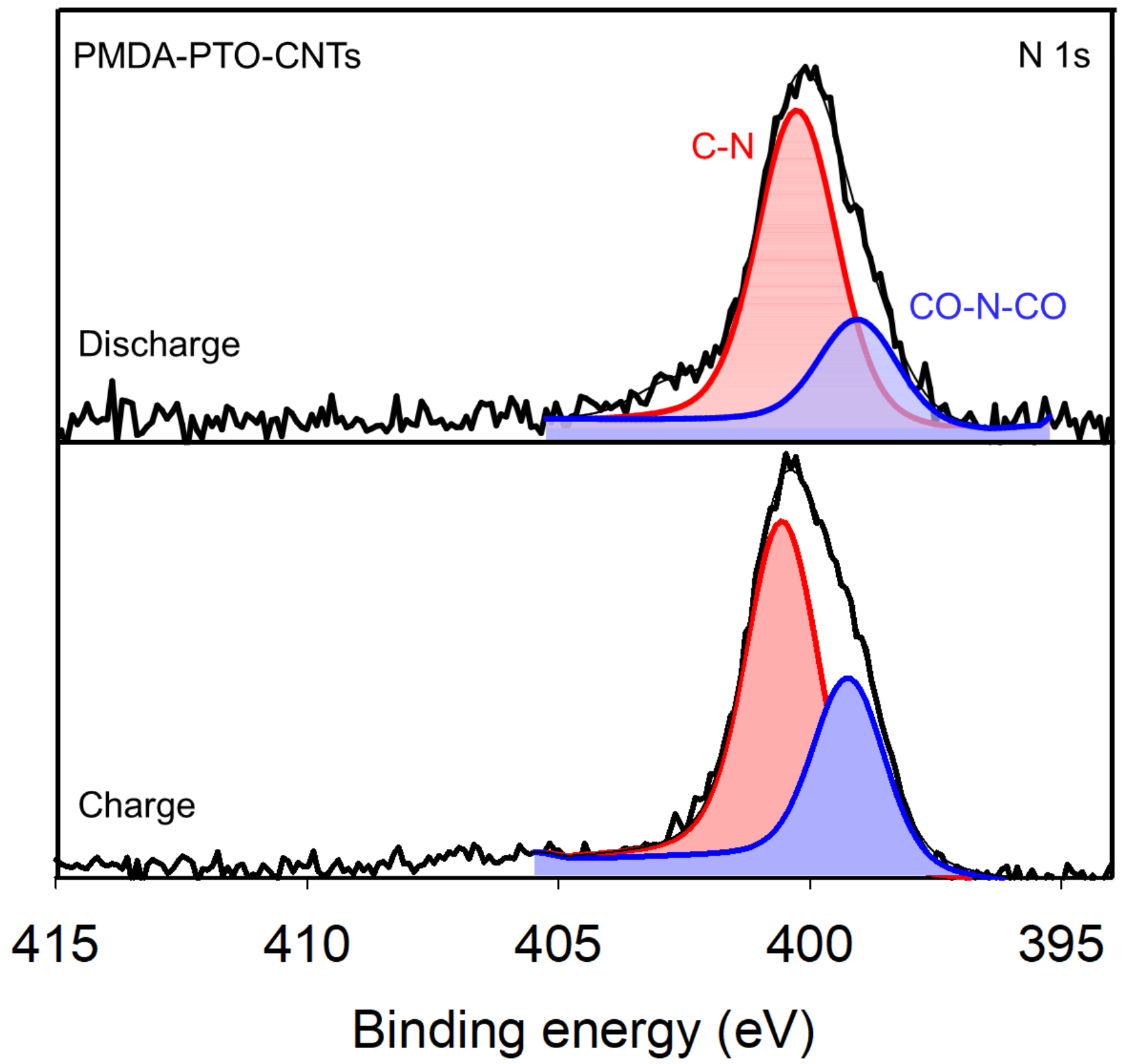

Figure S19. *Ex situ* N 1s XPS spectra of PMDA–PTO–CNT at different electrochemical states.

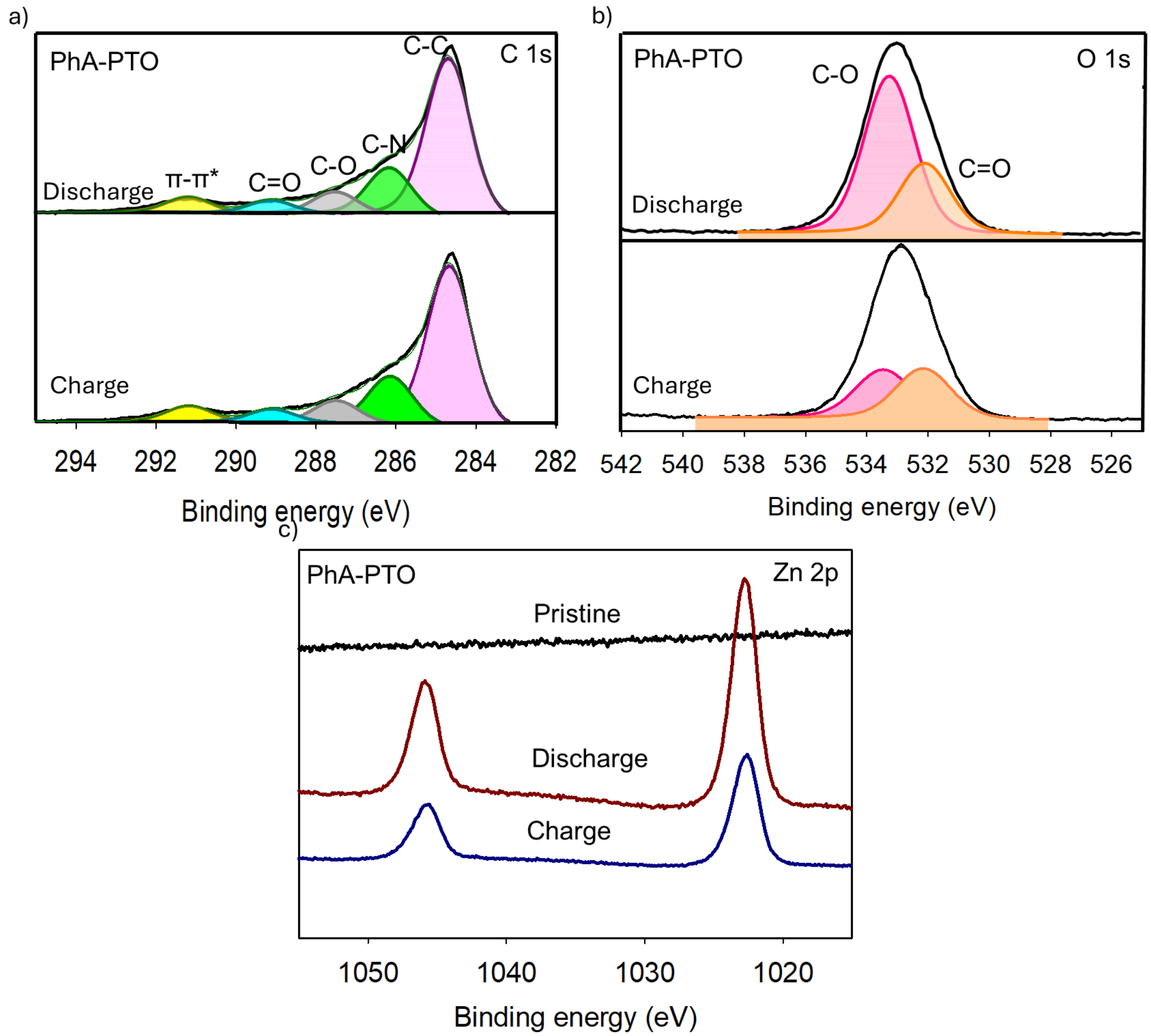

Figure S20. *Ex situ* XPS spectra of PhA–PTO: (a) C 1s, (b) O 1s, (c) Zn 2p, and (d) N 1s at different electrochemical states.

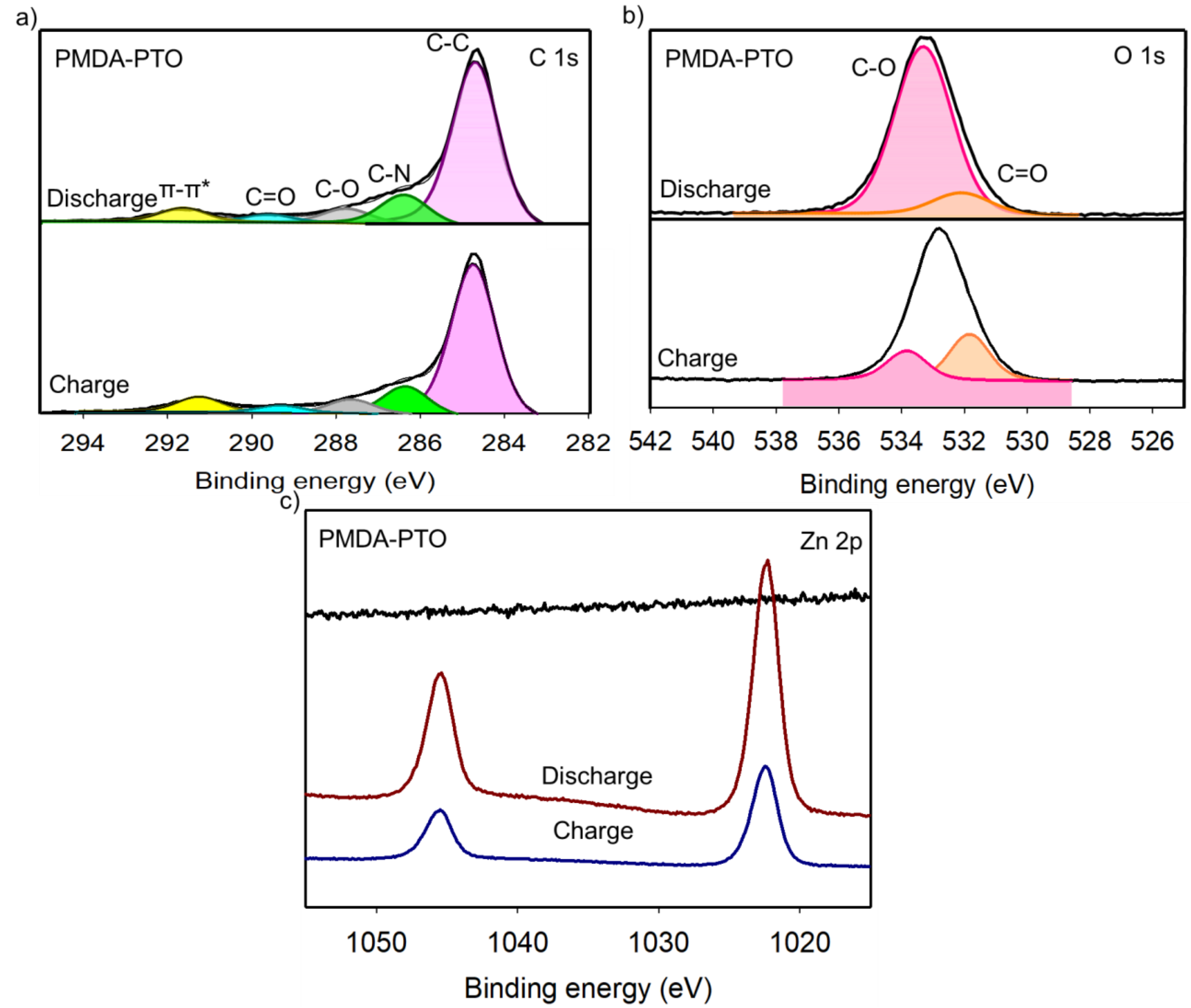

Figure S21. *Ex situ* XPS spectra of PMDA–PTO: (a) C 1s, (b) O 1s, (c) Zn 2p, and (d) N 1s at different electrochemical states.

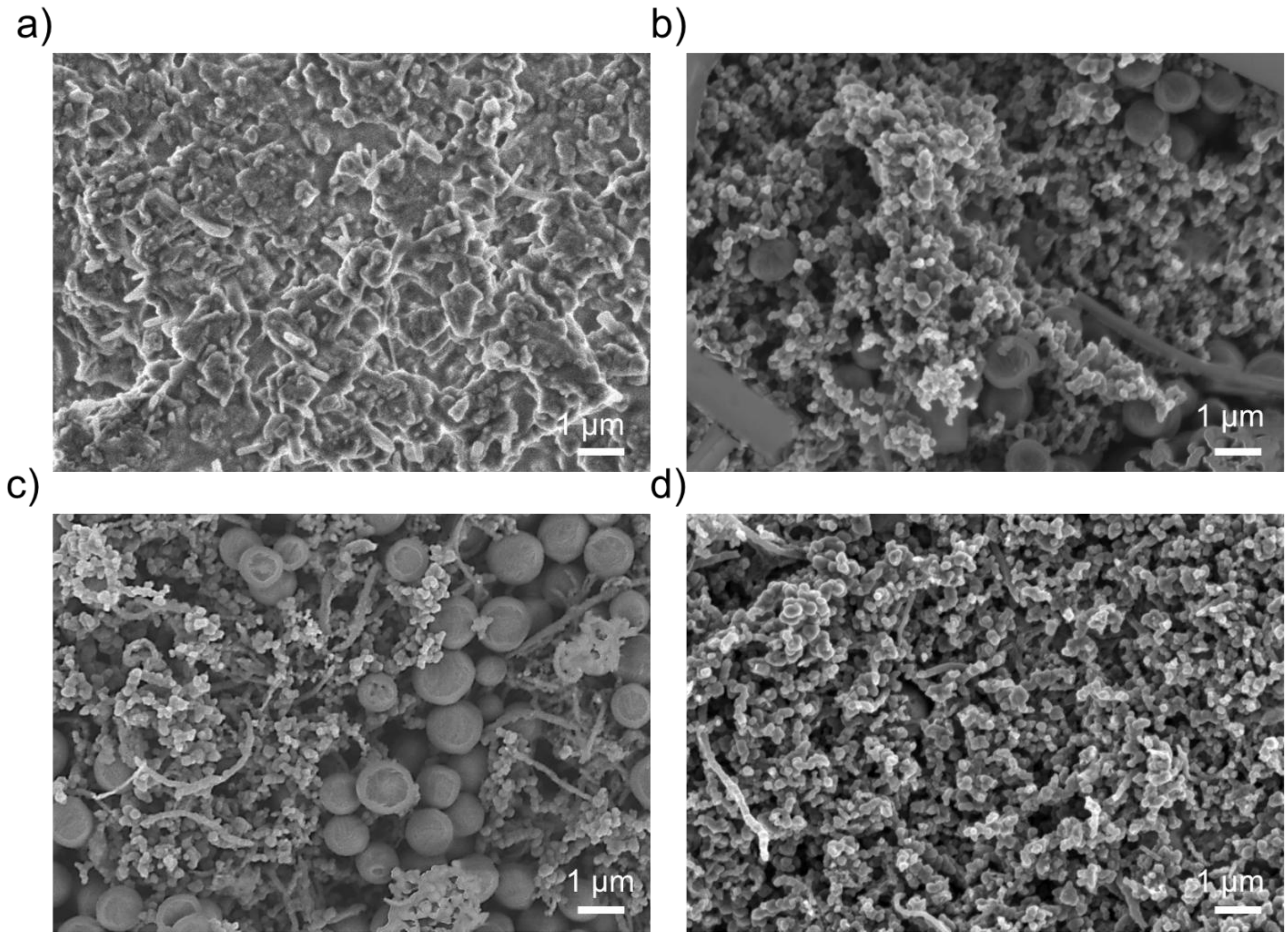

Figure S22. Higher-magnification SEM images of PMDA–PTO and PMDA–PTO–CNT electrodes before and after prolonged electrochemical cycling. (a,b) PMDA–PTO before and after cycling, respectively. (c,d) PMDA–PTO–CNT before and after cycling, respectively.

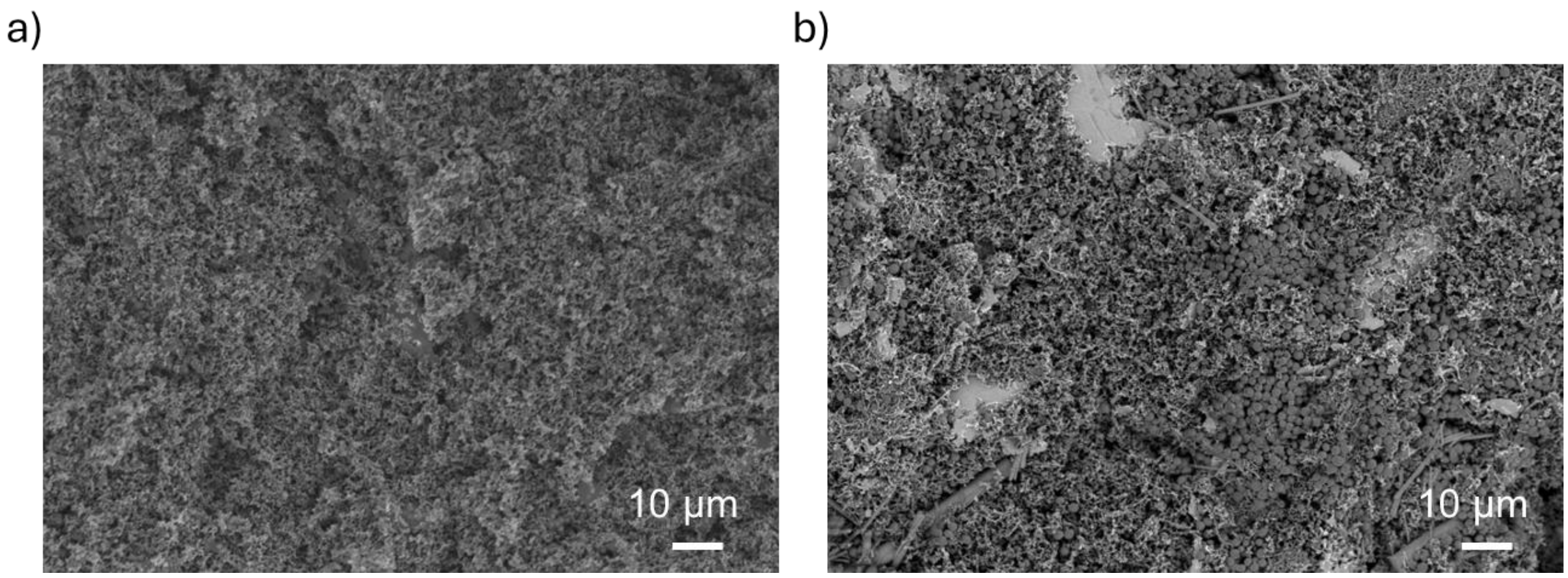

Figure S23. SEM images of (a) PMDA–PTO and (b) PMDA–PTO–CNT electrodes after the rate capability test at a high current density of 20 A g^{-1}.

6. Mechanistic studies

The Zn^{2+} storage mechanism was investigated by cyclic voltammetry (CV) at various scan rates for both PMDA–PTO and PMDA–PTO–CNT electrodes. The analysis was based on the power-law relationship between peak current and scan rate (Equation S4):

$$i = av^{b} \qquad S4$$

where *i* is the peak current, *v* is the scan rate, and *a* and *b* are fitting parameters. To extract the *b* value, the relationship was linearized by taking the logarithm of both sides, as shown in Equation S5:

$$Log\ i = b\ Log\ v + Log\ a \qquad S5$$

The exponent *b* provides mechanistic insight into the charge-storage process: values close to 0.5 indicate diffusion-controlled behavior, whereas values approaching 1.0 correspond to surface-controlled (pseudocapacitive) processes. The *b* values were obtained from linear fitting of the log–log plots of peak current versus scan rate.[S4] As shown in Figures S23a,b, PMDA–PTO exhibits lower *b* values, indicating a greater diffusion-limited contribution to charge storage. In contrast, the PMDA–PTO–CNT hybrid displays *b* values closer to unity, highlighting a dominant capacitive contribution enabled by enhanced electronic conductivity.

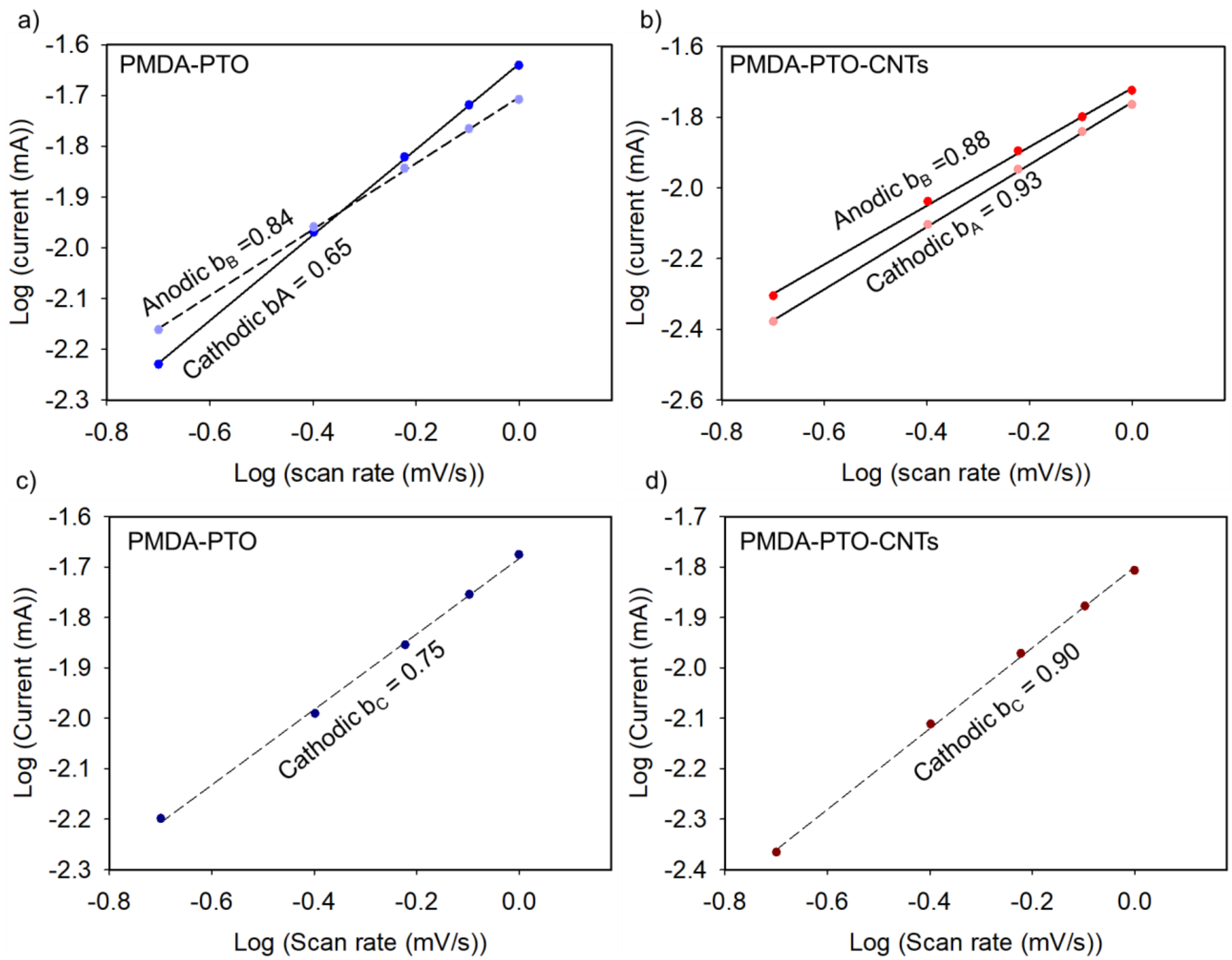

Figure S24. Log–log plots of peak current (i) versus scan rate (ν) with linear fitting for the anodic and cathodic peaks of (a) PMDA–PTO and (b) PMDA–PTO–CNT.

To complement the qualitative kinetic analysis, a quantitative evaluation based on Dunn's method was performed to distinguish capacitive and diffusion-controlled contributions in PMDA–PTO and PMDA–PTO–CNT electrodes.[S21] In this approach, the current response is analyzed at fixed potentials, where the capacitive contribution scales linearly with the scan rate (ν), whereas the diffusion-controlled contribution scales with the square root of the scan rate ($\nu^{1/2}$). The total current response can be expressed as the sum of these two components (Equation S6):

$$i_{total} = i_{capacitive} + i_{diffusive} \qquad S6$$

This relationship can be rearranged as shown in Equation S7:

$$\frac{i\ (\nu)}{\nu^{1/2}} = k_1 \nu^{1/2} + k_2 \qquad S7$$

where $k_1$represents the surface-controlled (capacitive) contribution and $k_2$corresponds to the diffusion-controlled contribution.

To quantitatively evaluate Zn^{2+} diffusion kinetics, the Warburg coefficient (σ) was extracted from the linear fitting of the real impedance (Z') versus $\omega^{-1/2}$ in the low-frequency region (Figure S24). The Zn^{2+} diffusion coefficient (*D*) was calculated according to the standard Warburg relation:[S22,S23]

$$D = \frac{R^2T^2}{2A^2n^4F^4C^2\sigma^2} \qquad S8$$

where R is the gas constant, T is the absolute temperature, A is the electrode area, n is the number of electrons transferred, F is Faraday's constant, C is the concentration of Zn^{2+} ions, and σ is the Warburg coefficient.

For PMDA–PTO, a Warburg coefficient of σ ≈ 33 Ω $s^{1/2}$ was obtained, yielding a Zn^{2+} diffusion coefficient of approximately 4.9 × 10^{-15} cm^2 s^{-1}. In contrast, PMDA–PTO–CNT shows a significantly lower σ value of ≈10 Ω $s^{1/2}$, corresponding to a higher diffusion coefficient of ~5.4 × 10^{-14} cm^2 s^{-1}. This represents an ~11-fold enhancement in Zn^{2+} diffusion upon CNT incorporation, highlighting the critical role of the conductive network in facilitating rapid ion transport.

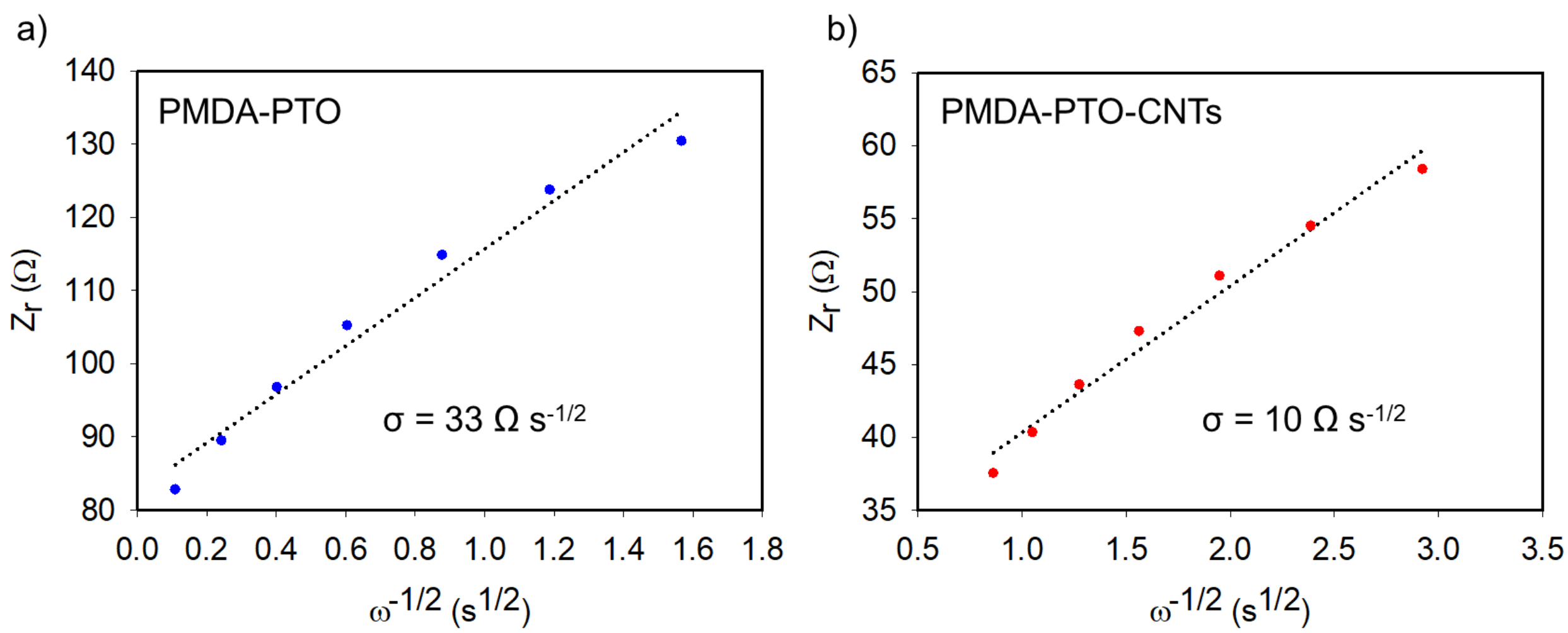

Figure S25. Linear fitting of Z' versus $\omega^{-1/2}$ derived from EIS measurements for (a) PMDA–PTO and (b) PMDA–PTO–CNT electrodes.

Table S3. Warburg coefficients (σ) and calculated Zn^{2+} diffusion coefficients (D) for PMDA–PTO and PMDA–PTO–CNT electrodes obtained from electrochemical impedance spectroscopy.

Material	**σ (Ω $s^{1/2}$)**	**D (cm^2 s^{-1})**
**PMDA-PTO**	33	4.9 x 10^{-15}
**PMDA-PTO-CNT**	10	5.4 x 10^{-14}

Diffusion coefficients were calculated from the Warburg region of the Nyquist plots measured at open-circuit voltage after 10 cycles using the standard Warburg relation.

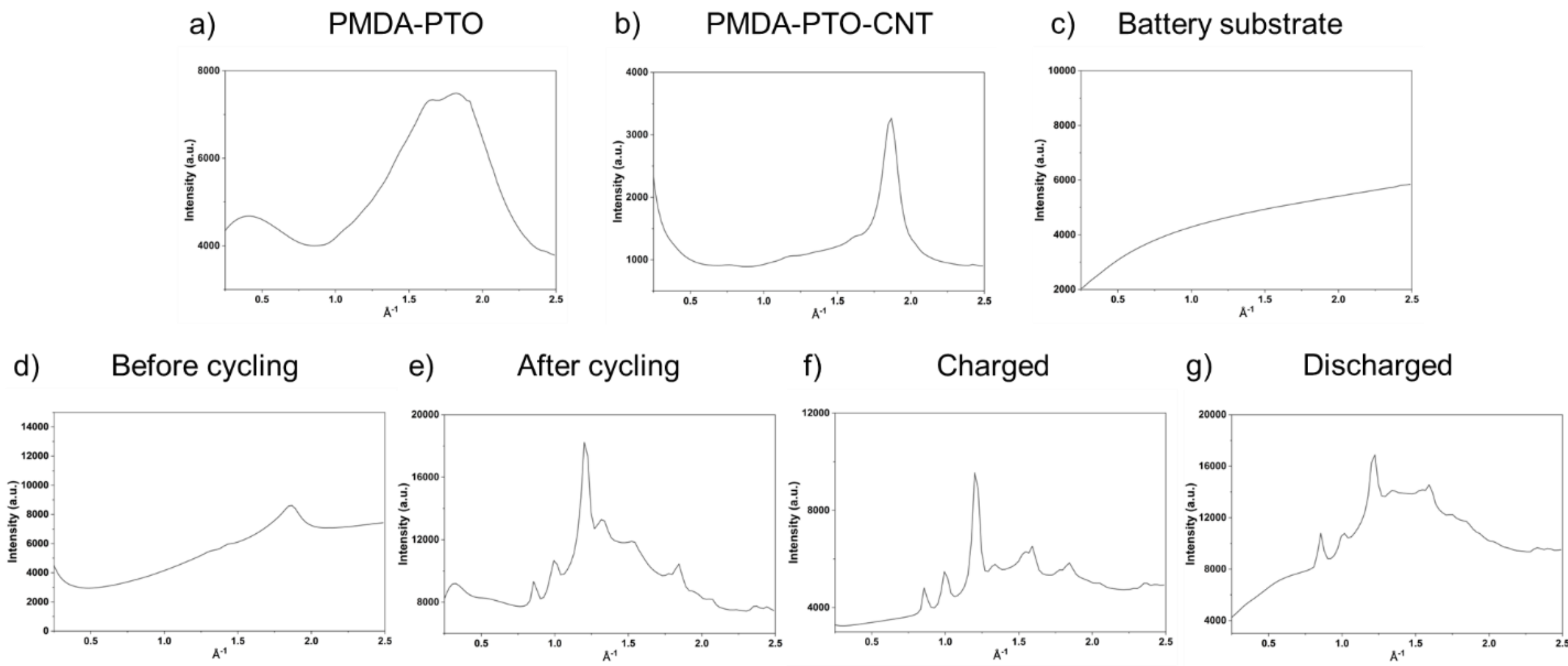

Figure S26. One-dimensional GIWAXS line cuts extracted from the corresponding 2D patterns for the (a) PMDA-PTO, (b) PMDA-PTO-CNT, (c) bare substrate (stainless steel foil), and assembled electrodes measured (d) before cycling, (e) after extended electrochemical cycling, (f) in the discharged, and (g) charged states.

One-dimensional intensity profiles were extracted from the corresponding two-dimensional GIWAXS patterns to aid visualization of the dominant scattering features (Figure S25). All profiles are characterized by broad maxima rather than sharp Bragg reflections, confirming the absence of long-range crystallinity across pristine, composite, and cycled electrodes. The evolution of the broad scattering features with electrode assembly and electrochemical cycling is consistent with the 2D GIWAXS analysis presented in the main text, supporting a Zn^{2+} storage mechanism governed by short-range molecular correlations and electrode-level organization rather than crystalline phase transformations.

7. Computational details

Computational Methods

The present calculations were intended to isolate the intrinsic electronic structure and reduction behavior of the conjugated PMDA–PTO backbone, enabling direct evaluation of charge delocalization and accessible redox states within the conjugated polymer framework. The experimentally observed Zn^{2+} storage mechanism is supported by ex situ FTIR and XPS analyses, while the DFT calculations provide complementary insight into the electronic evolution of the carbonyl-containing framework upon reduction. All quantum-chemical calculations were carried out using the Gaussian 16 software package. A three-repeat-unit oligomer was employed as a representative model of the PMDA–PTO polymer backbone, with terminal imide nitrogen atoms capped by hydrogen atoms to avoid artificial charge localization at the chain ends. Geometry optimizations were performed using the range-separated hybrid density functional ωB97X-D, which reliably accounts for long-range charge delocalization and dispersion interactions, in conjunction with the def2-TZVP basis set.[S24–S26] Solvent effects were incorporated using the SMD implicit solvation model with water as the solvent, consistent with the aqueous Zn^{2+} electrolyte used experimentally.[S27] For each charge state (0, 1−, 2−, 1+, and 2+), multiple spin multiplicities were examined, and the lowest-energy state was selected. Harmonic frequency calculations were conducted at the same level of theory to confirm that all optimized structures correspond to true minima on the potential energy surface (no imaginary frequencies) and to obtain zero-point energy and thermal corrections. Gibbs free energies were evaluated at 298 K.

Adiabatic redox free energies were calculated from differences in Gibbs free energies between the relevant oxidized and reduced states. Because absolute redox potentials depend sensitively on reference electrodes and solvation parameters, the calculations are used to assess relative redox energetics and qualitative agreement with experimental cyclic voltammetry rather than absolute potential alignment.[S28,S29]

DFT Analysis of Redox States

The calculated Gibbs free energies for the neutral, reduced, and oxidized states of the PMDA–PTO oligomer are summarized in Table S4. For the reduced species, both singlet and triplet spin configurations were considered for the dianion. The triplet dianion was found to be energetically

favored over the singlet by approximately 0.83 eV (≈80 kJ mol^{-1}), establishing a triplet ground state for the doubly reduced polymer. The computed adiabatic free-energy changes for the two sequential reduction steps are $\Delta G^o(0 \rightarrow 1-) = -333.3$ kJ mol^{-1} and $\Delta G^o(1- \rightarrow 2-) = -362.6$ kJ mol^{-1}. The relatively small difference between these values indicates that insertion of the second electron is thermodynamically favorable once the radical anion has formed, consistent with efficient multi-electron redox behavior. For oxidation, the free-energy changes $\Delta G^o(1+ \rightarrow 0) = -548.3$ kJ mol^{-1} and $\Delta G^o(2+ \rightarrow 1+) = -659.6$ kJ mol^{-1} indicate substantially higher energetic requirements compared to reduction, explaining why oxidation appears experimentally as a single broad anodic wave rather than two resolved processes.

Frontier molecular orbital analysis shows that the LUMO is delocalized across both the imide and pyrene-tetrone units, supporting carbonyl-centered reduction with extensive charge delocalization, whereas the HOMO lies deep in energy, consistent with the intrinsically low electronic conductivity of the pristine polymer (Figure S26). These features rationalize the experimentally observed two-electron reduction and the dramatic kinetic enhancement achieved upon hybridization with conductive CNT networks.

Table 4. Calculated Gibbs free energies and redox energetics of PMDA–PTO oligomer

Charge state	Spin multiplicity	Gibbs free energy
Neutral (0)	Singlet	−5187.592949
Anion (1-)	Doublet	−5187.719895
Dianion (2-)	Triplet	−5187.857999
Dianion (2-)	Singlet	−5187.8274 (higher energy)
Cation (1+)	Doublet	−5187.384121
Dication (2+)	Singlet	−5187.132883

Table S5. Calculated adiabatic redox free energies

Redox step	Δ G° (KJ mol^{-1})
0 → 1−	−333.3
1− → 2−	−362.6
1+ → 0	−548.3
2+ → 1+	−659.6

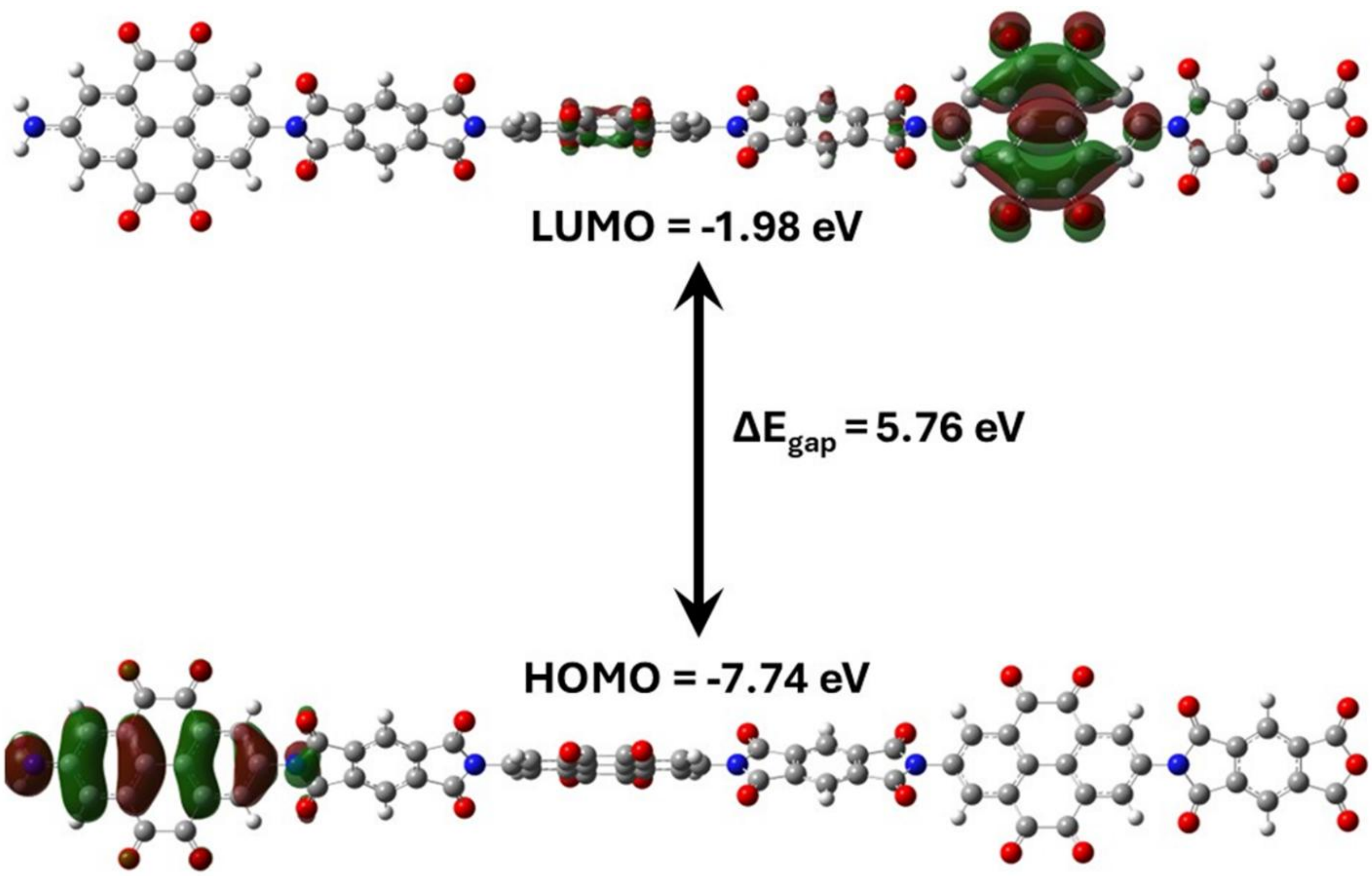

Figure S27. HOMO/LUMO isosurfaces and energy levels of PMDA–PTO oligomer.

References

[S1] Z. Jiang, *J Appl Crystallogr* **2015**, *48*, 917.

[S2] J. A. Letizia, S. Cronin, R. P. Ortiz, A. Facchetti, M. A. Ratner, T. J. Marks, *Chemistry A European J* **2010**, *16*, 1911.

[S3] T. Nokami, T. Matsuo, Y. Inatomi, N. Hojo, T. Tsukagoshi, H. Yoshizawa, A. Shimizu, H. Kuramoto, K. Komae, H. Tsuyama, J. Yoshida, *J. Am. Chem. Soc.* **2012**, *134*, 19694.

[S4] Q. Li, H. Wang, H. Wang, Z. Si, C. Li, J. Bai, *ChemSusChem* **2020**, *13*, 2449.

[S5] H. H. Farrag, J. Faleiro Berbigier, J. T. Liu, E. Grignon, D. S. Seferos, *ACS Appl. Mater. Interfaces* **2025**, *17*, 32329.

[S6] H. Gao, A. R. Neale, Q. Zhu, M. Bahri, X. Wang, H. Yang, Y. Xu, R. Clowes, N. D. Browning, M. A. Little, L. J. Hardwick, A. I. Cooper, *J. Am. Chem. Soc.* **2022**, *144*, 9434.

[S7] H. H. Farrag, E. Grignon, A. M. Battaglia, J. T. Liu, D. S. Seferos, *RSC Appl. Polym.* **2025**, *3*, 370.

[S8] L. Shao, C. Crockford, H. Geen, G. Grasso, J. J. Titman, *J. Magn. Reson.* **2004**, *167*, 75.

[S9] I. Hung, Z. Gan, *J. Magn. Reson.* **2011**, *213*, 196.

[S10] R. M. Orr, M. J. Duer, S. E. Ashbrook, *J. Magn. Reson.* **2005**, *174*, 301.

[S11] A. Mal, J. Caroni, A. Patriarchi, O. Luzanin, R. Ramos, J. Bitenc, M. Melle-Franco, M. Souto, *Adv. Mater.* **2025**, e12950.

[S12] J. Kumankuma-Sarpong, S. Tang, W. Guo, Y. Fu, *ACS Appl. Mater. Interfaces* **2021**, *13*, 4084.

[S13] Z. Lin, H.-Y. Shi, L. Lin, X. Yang, W. Wu, X. Sun, *Nat Commun* **2021**, *12*, 4424.

[S14] Z. Guo, Y. Ma, X. Dong, J. Huang, Y. Wang, Y. Xia, *Angewandte Chemie* **2018**, *130*, 11911.

[S15] H. Zhang, Y. Fang, F. Yang, X. Liu, X. Lu, *Energy Environ. Sci.* **2020**, *13*, 2515.

[S16] S. Zhang, S. Long, H. Li, Q. Xu, *Chemical Engineering Journal* **2020**, *400*, 125898.

[S17] F. Ye, Q. Liu, H. Dong, K. Guan, Z. Chen, N. Ju, L. Hu, *Angewandte Chemie* **2022**, *134*, e202214244.

[S18] X. Wang, J. Xiao, W. Tang, *Adv Funct Materials* **2022**, *32*, 2108225.

[S19] J. Liang, Y. Hou, Y. You, L. Dong, B. Mei, H. Niu, *J. Mater. Chem. C* **2024**, *12*, 4655.

[S20] O. Buyukcakir, R. Yuksel, F. Begar, M. Erdogmus, M. Arsakay, S. H. Lee, S. O. Kim, R. S. Ruoff, *ACS Appl. Energy Mater.* **2023**, *6*, 7672.

[S21] B. E. Conway, *Electrochemical Supercapacitors*, Springer US, Boston, MA, **1999**.

[S22] G. Gao, X. Wang, L. Chen, T. Baumgartner, X. He, *Chem. Mater.* **2021**, *33*, 4596.

[S23] E. Grignon, A. M. Battaglia, J. T. Liu, B. T. McAllister, D. S. Seferos, *ACS Appl. Mater. Interfaces* **2023**, *15*, 45345.

[S24] J. Aragó, J. C. Sancho-García, E. Ortí, D. Beljonne, *J. Chem. Theory Comput.* **2011**, *7*, 2068.

[S25] M. Gray, P. E. Bowling, J. M. Herbert, *J. Phys. Chem. A* **2024**, *128*, 7739.

[S26] F. Weigend, *Phys. Chem. Chem. Phys.* **2006**, *8*, 1057.

[S27] Z. Gong, D. Kong, N. Chen, F. Zhang, D. Wang, Z. Qian, J. Yang, *Adv Funct Mater* **2025**, e13415.

[S28] M.-H. Baik, R. A. Friesner, *J. Phys. Chem. A* **2002**, *106*, 7407.

[S29] Q. He, B. Yu, Z. Li, Y. Zhao, *Energy & Environ Mater* **2019**, *2*, 264.
